# QM-HiFSA-Aided Structure Determination of Succinilenes A–D, New Triene Polyols from a Marine-Derived *Streptomyces* sp.

**DOI:** 10.3390/md15020038

**Published:** 2017-02-14

**Authors:** Munhyung Bae, So Hyun Park, Yun Kwon, Sang Kook Lee, Jongheon Shin, Joo-Won Nam, Dong-Chan Oh

**Affiliations:** 1 Natural Products Research Institute, College of Pharmacy, Seoul National University, Seoul 08826, Korea; baemoon89@snu.ac.kr (M.B.); hanirela@snu.ac.kr (S.H.P.); kisi2016@snu.ac.kr (Y.K.); sklee61@snu.ac.kr (S.K.L.); shinj@snu.ac.kr (J.S.); 2 College of Pharmacy, Yeungnam University, Gyeongsan, Gyeongbuk 38541, Korea; 3 Department of Medicinal Chemistry & Pharmacognosy, College of Pharmacy, University of Illinois at Chicago, Chicago, IL 60612, USA

**Keywords:** marine actinomycete, QM-HiFSA, NMR, anti-inflammatory

## Abstract

Based on profiles of secondary metabolites produced by marine bacteria obtained using LC/MS, succinilenes A–D (**1**–**4**), new triene polyols, were discovered from a culture of a *Streptomyces* strain SAK1, which was collected in the southern area of Jeju Island, Republic of Korea. The gross structures of **1**–**4** were primarily determined through analysis of NMR spectra. The double bond geometries of the succinilenes, which could not be established from conventional ^1^H NMR spectra because of the highly overlapped olefinic signals, were successfully deciphered using the recently developed quantum-mechanics-driven ^1^H iterative full spin analysis (QM-HiFSA). Succinilenes A–C (**1**–**3**) displayed inhibitory effects against lipopolysaccharide (LPS)-induced nitric oxide (NO) production, indicating their anti-inflammatory significance. These three compounds (**1**–**3**) commonly bear a succinic acid moiety, although succinilene D (**4**), which did not inhibit NO production, does not have this moiety in its structure. The absolute configurations of succinilenes A–D (**1**–**4**) were established through *J*-based configuration analysis, the modified Mosher’s method following methanolysis, and CD spectral analysis.

## 1. Introduction

Precise interpretation of the spectroscopic data of molecules is the critical step in natural product research for drug discovery. Nuclear magnetic resonance (NMR) spectroscopy is a predominant method used to elucidate the structures of natural products. However, NMR spectra, even when acquired at a high magnetic field, are sometimes misinterpreted or hardly interpretable due to a lack of signal dispersion or the occurrence of higher order effects. Quantum-mechanics-driven ^1^H iterative full spin analysis (QM-HiFSA) [1] is an emerging NMR interpretation tool that can reduce errors and ambiguities in the analysis of highly overlapped ^1^H NMR spectra. HiFSA allows for the complete and precise interpretation of ^1^H NMR parameters such as coupling constants (*J*_HH_), chemical shifts (*δ*_H_), and line widths (Δ*ν*_1/2_), on the basis of both structural and spectral line shape considerations [2,3]. 

**Figure 1:**
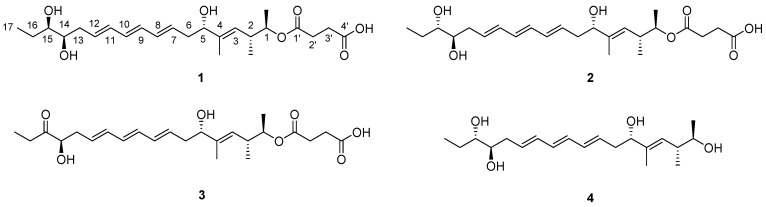
Structures of succinilenes A–D (**1**–**4**).

Similar to the importance of using combinations of chemical and computational analyses, it is critical to focus on prolific sources of structurally and biologically novel compounds because of the constant clinical need for new chemical entities with pharmaceutical potential [4]. As part of our efforts to discover new bioactive natural products, we have been investigating the chemistry of actinobacteria derived from marine and saline environments including salterns [5,6,7], intertidal zones [8,9,10,11], and deep-sea sediments [12]. In our previous reports, novel bioactive secondary metabolites were mainly identified through the chemical profiling of bacterial secondary metabolites by LC/MS analysis. Based on this approach and focusing on actinobacterial chemical profiles, we have continuously isolated actinomycete strains from the southern area of Jeju Island, Republic of Korea. Our comprehensive time-course chemical screening of the marine actinomycete strain collection resulted in the discovery of *Streptomyces* strain SAK1, which was revealed to produce a series of previously-unreported secondary metabolites, each bearing a triene moiety (UV *λ*_max_ at 270 nm), in the exponential phase of the culture (in 2~3 days after inoculation). Because the strain SAK1 interestingly produced tripartilactam [13] and sceliphrolactam [14], macrocyclic lactams originally reported from insect-associated *Streptomyces* strains, and the triene-bearing compounds disappeared as reaching the stationary phase of the culture on day 4, a precise time-course study of the culture was performed to maximize the production of these new metabolites. Through a large-scale fermentation of this strain and the isolation of these compounds using High Performance Liquid Chromatography (HPLC), four new triene polyol metabolites, succinilenes A–D (**1**–**4**), with inhibitory activities against lipopolysaccharide (LPS)-induced nitric oxide (NO) production were obtained. The gross structures of these new compounds were elucidated by conventional 1D and 2D NMR spectroscopy. However, their overlapped ^1^H NMR signals, especially in the olefinic region, required the application of QM-HiFSA to determine the geometries of their triene moieties. Herein, we report the isolation, structure elucidation, and biological activities of succinilenes A–D (**1**–**4**) (Figure 1), emphasizing a successful application of the QM-HiFSA technique for the complete assignment of ^1^H-^1^H coupling constants, even from complicated high-order ^1^H peaks.

## 2. Results

### 2.1. Structural Elucidation

Succinilene A (**1**) was isolated as a yellow gum, and its molecular formula was deduced as C_24_H_38_O_7_ based on HR-FAB mass spectrometry (obsd. [M − H]^−^ at *m*/*z* 437.2550, calcd. [M − H]^−^ 437.2539) in combination with ^1^H and ^13^C NMR data (Table 1). The ^1^H NMR spectrum of **1** in pyridine-*d*_5_ exhibited typical signatures of polyenes and polyols, with seven olefinic protons (*δ*_H_ 6.3664, 6.3158, 6.2929, 6.2885, 6.1280, 5.9506, and 5.5426) and four protons attached to oxygen-bearing carbons (*δ*_H_ 5.0898, 4.3745, 3.9176, and 3.7579). Further analysis of the ^1^H NMR spectrum of **1** revealed the existence of eleven aliphatic protons between *δ*_H_ 2.9046 and 1.8105, a singlet methyl group bound to an olefinic carbon (*δ*_H_ 1.8616), two doublet methyl groups (*δ*_H_ 1.1907 and 0.9973), and a triplet methyl group (*δ*_H_ 1.1619) bound to an *sp*^3^ aliphatic carbon. The ^13^C and gradient heteronuclear single quantum coherence spectroscopy (gHSQC) NMR data displayed two carbonyl carbons (*δ*_C_ 175.7 and 172.7), seven olefinic *sp*^2^ carbons (*δ*_C_ 132.8, 132.6, 132.5, 132.0, 131.7, 131.6, and 127.2), one quaternary olefinic carbon (*δ*_C_ 140.1), four oxygen-bound methine carbons (*δ*_C_ 76.8, 75.5, 74.2, and 74.1), six aliphatic carbons (*δ*_C_ 39.8, 38.0, 37.1, 30.4, 30.3, and 27.5), and four methyl group carbons (*δ*_C_ 17.2, 16.6, 12.2, and 10.9). Based on the UV absorption maximum at 270 nm and the eight olefinic carbon signals, succinilene A (**1**) was expected to bear three conjugated double bonds and an isolated double bond. These four double bonds along with two carbonyl functional groups accounted for all of the six double bond equivalents inherently calculated from the molecular formula, thus indicating that succinilene A (**1**) is a linear compound.

**Figure 2:**
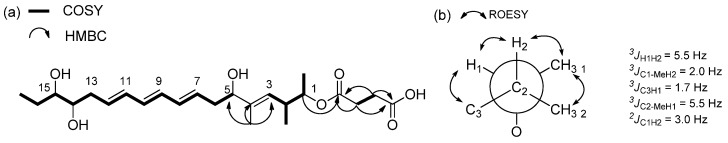
(**a**) Determination of the planar structure of succinilene A based on the analysis of key COSY and HMBC correlations; (**b**) *J*-based configuration analysis of succinilene A (**1**) at C-1 and C-2.

Given this information, all ^13^C-^1^H one-bond correlations were assigned by analysis of the gHSQC NMR spectrum (Table 1). Interpretation of COSY and HMBC NMR spectroscopic data readily led to the elucidation of the gross structure of succinilene A (**1**). First, the COSY signals from a terminal triplet methyl group (H_3_-17 at *δ*_H_ 1.1619) to aliphatic methylene protons (H_2_-16 at *δ*_H_ 1.8578 and 1.8105) connected C-17 (*δ*_C_ 10.9) to C-16 (*δ*_C_ 27.5). The aliphatic carbon C-16 was elucidated to be directly connected to the oxygenated carbon C-15 (*δ*_C_ 75.5) based on the COSY correlations between H-15 (*δ*_H_ 3.7579) and H-16. The homonuclear correlation between H-15 and H-14 (*δ*_H_ 3.9176) revealed that an oxygen-bound methine carbon (C-14 at *δ*_C_ 74.2) was connected to C-15, indicating the existence of a diol moiety. Further COSY analysis verified the connectivity between H-14 and H_2_-13 (*δ*_H_ 2.7253 and 2.6745), identifying the C-14–C-13 bond. The aliphatic carbon C-13 was connected to the *sp*^2^ carbon C-12 (*δ*_C_ 132.8) based on a 3-bond ^1^H-^1^H coupling signal between H-12 (*δ*_H_ 6.1280) and H-13. Additional COSY correlations among the six olefinic protons from H-12 to H-7 (*δ*_H_ 5.9506) indicated the construction of a triene moiety from C-12 to C-7 (*δ*_C_ 132.8 to 132.0), which is consistent with the UV absorption maximum at 270 nm. The triene moiety was located next to C-6 based on the ^3^*J*_HH_ couplings between H_2_-6 (*δ*_H_ 2.6519 and 2.5631) and H-7. The homonuclear correlations of H_2_-6 with the oxygenated proton (H-5 at *δ*_H_ 4.3745) showed C-6–C-5 (*δ*_C_ 76.8) connectivity. The HMBC correlations from a singlet methyl proton (H_3_-4-Me; *δ*_H_ 1.8616) to C-5, C-4 (*δ*_C_ 140.1), and C-3 (*δ*_C_ 127.2) indicated expansion of the chain from C-5 to C-3 through C-4. The olefinic methine C-3 was assigned next to C-2 (*δ*_C_ 37.1), with a branched methyl group (H_3_-2-Me; *δ*_H_ 0.9973/*δ*_C_ 16.6) attached to C-2. The COSY correlation from H-1 (*δ*_H_ 5.0898) to H-2 and H_3_-1-Me identified the connectivity of C-1 (*δ*_C_ 74.1) and C-2, establishing the first partial structure from C-17 to C-1, a chain composed of 20 carbons bearing three hydroxyl groups and a treiene moiety. Based on additional 2D NMR analysis, the aliphatic H_2_-2′ (*δ*_H_ 2.8516 and 2.8436) displayed ^3^*J*_HH_ and ^3^*J*_CH_ correlations to adjacent aliphatic H_2_-3′ peaks (*δ*_H_ 2.9015, and 2.8990) and two carbonyl carbons C-1′ and C-4′ (*δ*_C_ 172.7 and 175.7), completing the second partial structure as a succinic acid. These two partial structures were connected based on a key HMBC correlation from H-1, of which the deshielded chemical shift (*δ*_H_ 5.0898) was indicative for the existence of an ester linkage, to C-1′, resulting in the gross structure of **1** (Figure 2a). 

Succinilene B (**2**) was obtained as a yellow gum. The molecular formula was determined as C_24_H_38_O_7_ on the basis of HR-FABMS data (obsd. [M − H]^−^ at *m*/*z* 437.2550, calcd. [M − H]^−^ 437.2539) along with ^1^H and ^13^C NMR spectroscopic data (Table 1). The molecular formula and the UV spectrum of **2** were identical to those of **1**, indicating that succinilene B (**2**) is structurally analogous to succinilene A (**1**). The 1D and 2D NMR data of **2** also displayed very similar patterns to those of **1**, as expected. Further analysis of NMR spectroscopic data resulted in the construction of the gross structure of **2**, which was identical to that of **1**. Actually, most of the ^13^C chemical shifts of **2** were very similar to those of **1**, within 0.2 ppm. However, the ^13^C peaks of C-13 to C-16 around the oxymethine group located adjacent to the terminal ethyl group differed by 0.5~1 ppm compared to the same peaks in **1**, indicating that succinilene B (**2**) is a diastereomer of **1** that possesses different stereochemistry at C-13 or/and C-14.

Succinilene C (**3**) was purified as a yellow gum and determined to possess a molecular formula of C_24_H_36_O_7_ on the basis of HR-FABMS data (obsd. [M + H]^+^ at *m*/*z* 437.2562, calcd. [M + H]^+^ 437.2545) combined with ^1^H and ^13^C NMR spectral data (Table 2). Based on the molecular weight and UV spectrum of **3**, it was expected that one of the hydroxyl groups in succinilene C (**3**) had been oxidized to a ketone group. According to the comparison of the 1D and 2D NMR spectroscopic data of **3** with those of **1**, the oxygen-bound proton (H-15, *δ*_H_ 3.7579) in **1** was absent in **3**, and a ketone carbon signal (*δ*_C_ 214.0) was detected instead, clarifying that the hydroxyl group at the C-15 position in **1** had been oxidized to the ketone group in **3**. This assignment was also supported by the 3-bond HMBC correlations from H_3_-17 (*δ*_H_ 1.0890) and H_2_-13 (*δ*_H_ 2.7803 and 2.6591) to C-15 and H_3_-17/H-16 and H_2_-13/H-14 COSY correlations.

Succinilene D (**4**) was isolated as a yellow gum, and its molecular formula was deduced to be C_20_H_34_O_4_ based on the corresponding HR-FABMS data (obsd. [M + Na]^+^ at *m*/*z* 361.2347, calcd. [M + Na]^+^ 361.2355) and ^1^H and ^13^C NMR spectroscopic data (Table 2). The molecular weight of **4** was 100 Da lower than those of **1** and **2**, possibly indicating that the terminal succinic acid moiety in **1** and **2** might have been cleaved. Through the careful analysis of the 1D and 2D NMR spectral data, the gross structure of succinilene D (**4**) was elucidated to be the left part of **1** or **2**, which is the methanolysis product of **1** or **2** that lacks the succinic acid moiety.

The relative configurations of **1** were established by analyzing the ^1^H-^1^H coupling constants and hetero half-filtered total correlation spectroscopy (HETLOC) NMR data [15]. To determine the relationship between C-1 and C-2, *J*-based configuration analysis was performed with ^3^*J*_HH_, ^3^*J*_CH_, and ^2^*J*_CH_ values and ROESY NMR data [16], selecting the most suitable rotamer between C-1 and C-2 (Figure 2b). The relationship between H-1 and 2-Me was elucidated as *anti* based on the high ^3^*J*_CH_ value (5.5 Hz). The ^3^*J*_H1H2_ (5.5 Hz), ^3^*J*_C1-MeH2_ (2.0 Hz), and ^3^*J*_C3H1_ (1.7 Hz) values indicated *gauche* relationships for H-1/H-2, 1-Me/H-2, and C-3/H-1. Moreover, the small ^2^*J*_C1H2_ (3.0 Hz) value and the ROESY NMR signals between the H-1/H-2, H-1/H-3, H-2/1-Me, and 1-Me/2-Me led to select the rotamer depicted in Figure 2b, thus proposing 1R* and 2R* configuration.

**Figure 3:**
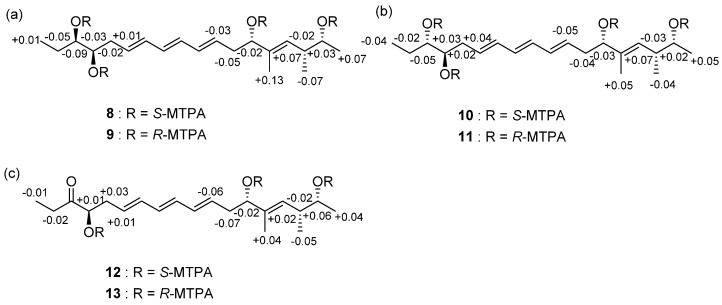
(**a**) Δδ*_S-R_* values of the MTPA esters (**8** and **9**) in pyridine-*d*_5_; (**b**) Δδ*_S-R_* values of the MTPA esters (**10** and **11**) in pyridine-*d*_5_; (**c**) Δδ*_S-R_* values of the MTPA esters (**12** and **13**) in pyridine-*d*_5_.

To determine the absolute configurations of the stereogenic centers at C-1, C-2, C-5, C-14, and C-15 in succinilene A (**1**), the modified Mosher’s method was applied [17]. Methanolysis was performed to detach the succinic acid moiety, which enabled derivatization of the hydroxyl group at C-1 with α-methoxy trifluoromethyl-phenylacetic acid (MTPA) chloride. After purifying the methanolysis product (**5**) of succinilene A, the secondary alcohols in **5** were derivatized using *R*- and *S*-MTPA-Cl. Analysis of the ^1^H and COSY NMR spectroscopic data for these *S*- and *R*-MTPA esters (**8** and **9**) led to the assignment of the Δδ*_S_*_-*R*_ values, establishing 1*R*, 5*S*, 14*R*, and 15*R* configurations (Figure 3a). Based on the established relative configuration, the absolute configuration of C-2 was determined to be 2*R*. The relative configurations and absolute configurations of succinilene B (**2**) were determined to be 1*R*, 2*R*, 5*S*, 14*R*, and 15*S* using the same procedure applied for **1** (Figure 3b). The relative and absolute configurations of succinilene C (**3**) were analogously established as 1*R*, 2*R*, 5*S*, and 15*R* through application of the modified Mosher’s method to the methanolysis product (**7**) of **3** (Figure 3c). Because the gross structure of succinilene D (**4**) was identical to the methanolysis products (**5** and **6**) of **1** and **2**, the ^1^H NMR and CD spectra of **4** were compared with those of **5** and **6**. The ^1^H NMR data for **6** was identical to those of **4** but was different from the ^1^H NMR spectrum of **5**, indicating that the relative configuration of **4** is identical to that of **6**. In addition, the CD spectra of **4** and **6** were identical, thus leading to the assignment of 1*R*, 2*R*, 5*S*, 14*R*, and 15*S* configurations for **4** (Figure S43).

Even though the absolute configurations of the stereogenic centers in **1**–**4** were completely assigned, the full assignments of the double bond geometries were not straightforward. The geometries of the double bonds of C-7/C-8 and C-11/C-12 in the succinilenes (**1**–**4**) were determined as 7*E* and 11*E* based on the H-7/H-8 and H-11/H-12 *trans*-coupling constant values. However, it was virtually impossible to clarify the double bond geometry of C-9/C-10 in **1**–**4** because the H-9 and H-10 olefinic protons were greatly overlapped, displaying second-order peaks in the ^1^H NMR spectra and, thus, hampering the accurate deconvolution of the ^1^H-^1^H coupling constants between H-9 and H-10, even at higher magnetic field (900 MHz). 

**Figure 4:**
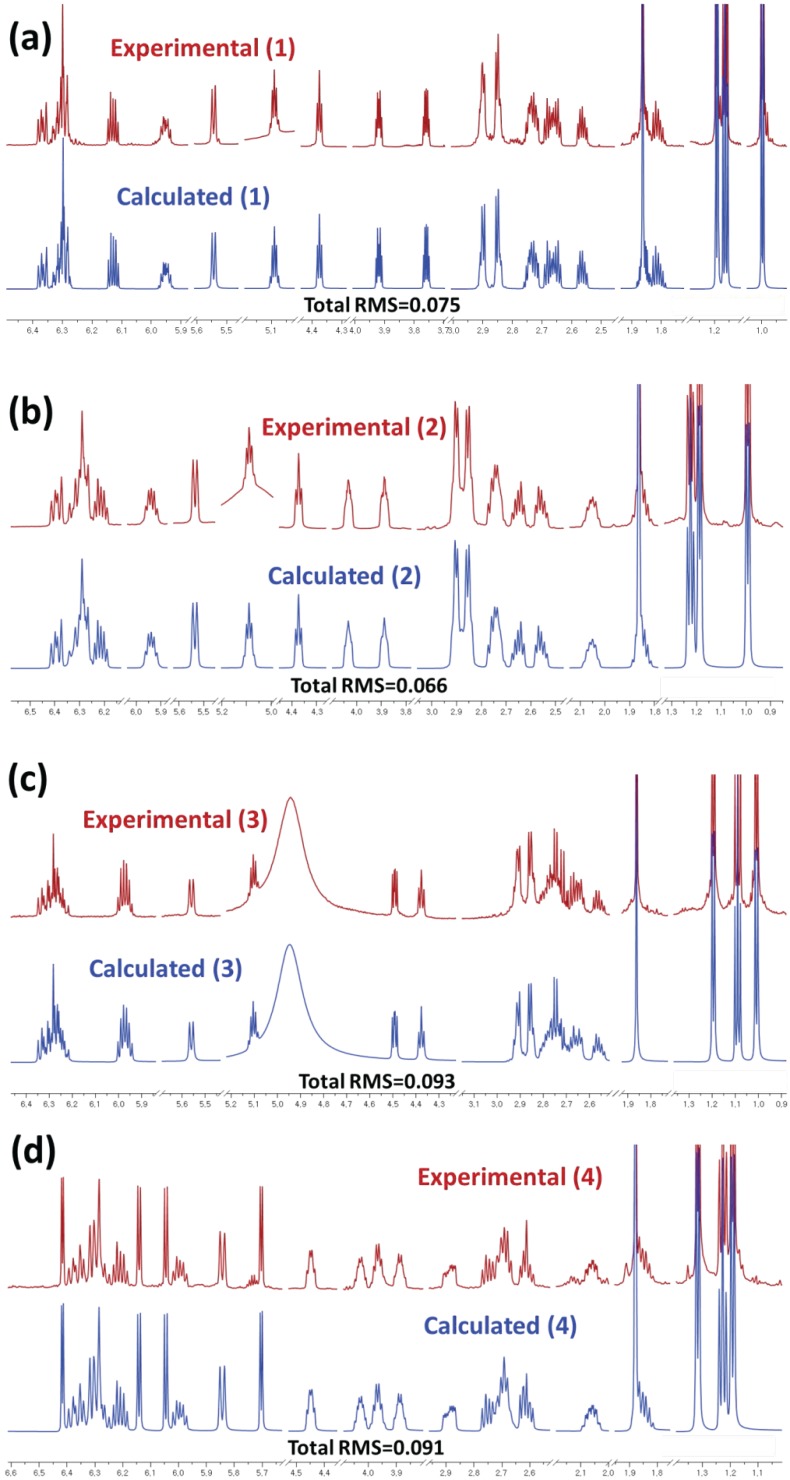
^1^H NMR fingerprints of compounds **1** (**a**)–**4** (**d**) generated by HiFSA. Comparison of the observed (red, obtained in pyridine-*d*_5_) and calculated (blue) ^1^H spectra.

Therefore, a recently developed computational analysis system, QM-HiFSA [1], was utilized to extract the H-9/H-10 coupling constants of the succinilenes using PERCH software. After geometry optimization and dynamic simulation of molecules using the MMS module, the basic ^1^H NMR parameters were predicted based on the 3D structural information. Then, the obtained initial parameters were optimized by iterative quantum mechanical simulation against experimental spectra. The iteration was repeated until both the simulated and experimental spectra were in excellent agreement with each other, with root mean square (RMS) values below 0.1 (RMS = 0.075 for **1**; RMS = 0.066 for **2**; RMS = 0.093 for **3**; RMS = 0.091 for **4**) (Figures S44–S47). Thus, the precise ^1^H NMR parameters were determined with high precision (chemical shifts: δ_H_, 0.1 ppb; coupling constants: *J*, 10 mHz) (Table 1 and Table 2) [18]. The *J* couplings of the highly overlapped olefinic proton signals (H-9 and H-10) of **1**–**4** were calculated to be 14.03 Hz, 14.79 Hz, 15.05 Hz, and 14.86 Hz (*cis J* couplings ca. 10–12 Hz; *trans J* couplings ca. 14–18 Hz) [18,19], respectively. These results confirmed the 9*E* geometry of the *trans*-olefinic protons (H-9 and H-10) in **1**–**4** (Figure 4).

### 2.2. Bioactivities of Succinilenes A–D

**Figure 5:**
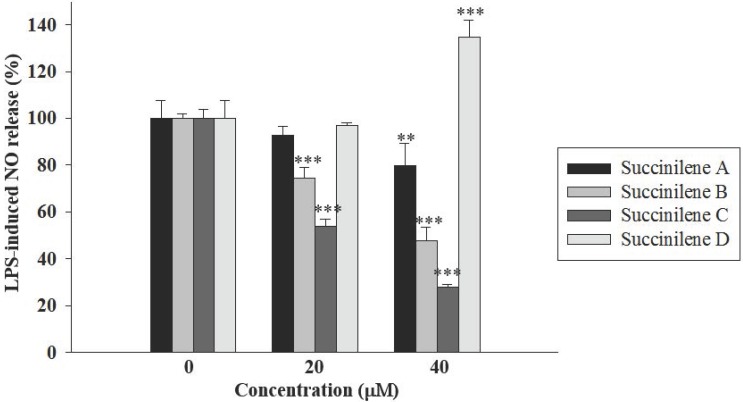
Inhibitory effects of succinilenes A–D (**1**–**4**) on LPS-induced NO production. RAW 264.7 cells (3 × 10^5^ cells/mL) were stimulated with LPS (1 µg/mL) in the presence or absence of **1**–**4**. After 20 h, the cultured media were collected, and the nitrite concentrations were measured using the Griess reaction. ** *p* < 0.01, *** *p* < 0.001 are considered statistically significant compared to the control group.

The biological activities of the succinilenes (**1**–**4**) were primarily evaluated for cytotoxicity against human cancer cells. In this assay, succinilene A showed moderate cytotoxicity against the gastric carcinoma cell line SNU638, with an IC_50_ value of 12.1 μg/mL (27.6 μM), whereas succinilenes B–D did not display significant inhibitory activities against the tested cancer cell lines. As part of the efforts to search for biological activities, a nitrate assay was performed for **1**–**4**. Overproduction of nitric oxide (NO) is highly associated with inflammation, and the pathway that regulates NO production is considered a useful target for anti-inflammatory agents [20]. RAW 264.7 cells were treated with LPS (1 µg/mL), which induces inflammatory responses [21,22]. When the cells were pretreated with various concentrations of the succinilenes (0, 20, or 40 µM) 30 min prior to LPS stimulation, NO production was gradually inhibited in a concentration-dependent manner, except for treatment with succinilene D (**4**). No significant effect on cell viability, as determined by the MTT assay (>90% cell survival), was observed at the tested concentrations, indicating that the inhibition of NO production by the tested compounds was not mediated by a cytotoxic effect. As a result, among the four compounds, succinilene C (**3**) exhibited the most remarkable inhibitory activity against NO production. By contrast, succinilene D, without succinic acid connected through an ester linkage, resulted in the overproduction of NO (Figure 5).

## 3. Experimental Section

### 3.1. General Experimental Procedures

Optical rotations were measured using a Jasco P-1020 polarimeter (sodium light source, JASCO, Easton, PA, USA) with a 1-cm cell. IR spectra were acquired on a Thermo Nicolet iS10 spectrometer (Thermo, Madison, CT, USA). UV spectra were obtained with a Perkin Elmer Lambda 35 UV-VIS spectrometer (Perkin Elmer, Waltham, MA, USA). Electrospray ionization (ESI) low-resolution LC/MS data were recorded on an Agilent Technologies 6130 quadrupole mass spectrometer coupled to an Agilent Technologies 1200 series HPLC (Agilent Technologies, Santa Clara, CA, USA). High-resolution fast-atom bombardment (HR-FAB) mass spectra were collected with a Jeol JMS-600W high-resolution mass spectrometer (Jeol, Tokyo, Japan) at the National Center for Inter-University Research Facilities (Gwanak-gu, Seoul, Korea). ^1^H, ^13^C, and 2D NMR spectra were obtained on a Bruker Avance 600 MHz spectrometer (Bruker, Billerica, MA, USA) at the NCIRF and a Bruker Avance 900 MHz NMR spectrometer at the Korea Basic Science Institute (Ochang, Chungcheongbuk-do, Korea).

### 3.2. Isolation of Bacteria, Cultivation, and Extraction

A sediment sample was obtained from the southern area of Jeju Island in a 40-mL sterilized plastic tube. The sample (1 g) was dried at room temperature (rt) for 3 h and diluted in 4 mL of sterilized distilled water. The mixture was spread on actinomycete isolation agar, chitin-based agar, SC medium (1 L of distilled water, 10 g of starch, 0.3 g of casein, 2.0 g of KNO_3_, 2.0 g of K_2_HPO_4_, 0.05 g of MgSO_4_·7H_2_O, 0.02 g of CaCO_3_, 0.01 g of FeSO_4_·7H_2_O, 16 g of agar, and 100 mg/L cycloheximide), and A4 medium (1 L of distilled water, 18 g of agar, and 100 mg/L cycloheximide). The strain SAK1 was isolated on SC medium. Based on the analysis of the 16S rDNA sequence, SAK1 was clarified to be most closely related to *Streptomyces flavogriseus* (99% identity, GenBank accession number: KY484916). For the production of the succinilenes, the strain SAK1 was initially cultivated in 50 mL of YEME media (4 g of yeast extract, 10 g of malt extract, and 4 g of glucose in 1 L of distilled water) in a 125 mL Erlenmeyer flask. This seed culture was cultivated for three days on a rotary shaker at 200 rpm at 30 °C. Ten milliliters of the seed culture was inoculated into 1 L of YEME medium in 2.8-L Fernbach flasks (12 ea × 1 L, total volume of 12 L). After two days of incubation, the whole culture (12 L) was extracted twice with 18 L of ethyl acetate. The ethyl acetate layer was separated by a fractionating funnel and dried over anhydrous sodium sulfate. The ethyl acetate extract was concentrated *in vacuo* to yield 2 g of dried material. This procedure was repeated six times (72 L of culture; total amount of extract: 12 g) to obtain a sufficient amount of the succinilenes for the structure elucidation and bioassays.

### 3.3. Isolation of Succinilenes A–D

The organic extract of the bacterial strain SAK1 culture was adsorbed on Celite, and the extract-Celite mixture was loaded onto a 2-g Sep-Pak C_18_ cartridge for flash column chromatography. Then, the mixture was fractionated with 20 mL each of 20%, 40%, 60%, 80%, and 100% MeOH in water and 1:1 MeOH/dichloromethane. Succinilenes A–D (**1**–**4**) were detected in the 80% and 100% MeOH/water fractions by LC/MS analysis. To obtain pure succinilenes A–D (**1**–**4**), the fractions bearing **1**–**4** were subjected to reversed-phase HPLC (Kromasil C_18_ : 250 × 10 mm, 5 µm) with gradient elution (30% acetonitrile/water to 65% acetonitrile/water over 40 min; UV detection at 280 nm; flow rate: 2 mL/min). Four major HPLC peaks were collected at retention times of 20 min, 28 min, 29 min, and 35 min. These major compounds were further purified under isocratic solvent conditions (65% methanol/water; UV detection at 280 nm; flow rate: 2 mL/min) on a reversed-phase HPLC column (Kromasil C_18_ : 250 × 10 mm, 5 µm). Finally, succinilenes A–D (**1**–**4**) were isolated as pure compounds at retention times of 33 min (12 mg), 31 min (11 mg), 36 min (5 mg), and 14 min (2 mg), respectively.

#### 3.3.1. Succinilene A (**1**)

[α]_D_ −5, (*c* 0.05, MeOH); UV (MeOH) *λ*_max_ (log ε) 270 (3.92) nm; IR (neat) ν_max_ 3405, 2935, 1647, 1450 cm^−1^; for ^1^H and ^13^C NMR data, see Table 1; HRFABMS *m*/*z* 437.2550 [M − H]^−^ (calcd. for C_24_H_37_O_7_ 437.2539).

#### 3.3.2. Succinilene B (**2**)

[α]_D_ −8, (*c* 0.05, MeOH); UV (MeOH) *λ*_max_ (log ε) 270 (3.89) nm; IR (neat) ν_max_ 3410, 2930, 1652, 1452 cm^−1^; for ^1^H and ^13^C NMR data, see Table 1; HRFABMS *m*/*z* 437.2550 [M − H]^−^ (calcd. for C_24_H_37_O_7_ 437.2539).

#### 3.3.3. Succinilene C (**3**)

[α]_D_ −3, (*c* 0.05, MeOH); UV (MeOH) *λ*_max_ (log ε) 270 (3.94) nm; IR (neat) ν_max_ 3392, 2944, 1647, 1452 cm^−1^; for ^1^H and ^13^C NMR data, see Table 2; HRFABMS *m*/*z* 437.2545 [M + H]^+^ (calcd. for C_24_H_37_O_7_ 437.2562).

#### 3.3.4. Succinilene D (**4**)

[α]_D_ −6, (*c* 0.05, MeOH); UV (MeOH) *λ*_max_ (log ε) 270 (3.85) nm; IR (neat) ν_max_ 3407, 2924, 1650, 1454 cm^−1^; for ^1^H and ^13^C NMR data, see Table 2; HRFABMS *m*/*z* 361.2347 [M + Na]^+^ (calcd. for C_20_H_34_O_4_Na 361.2355).

### 3.4. Methanolysis of Succinilenes A–C

Succinilene A (**1**) (5 mg) was dissolved in 2 mL of MeOH, and 28 mg of NaOMe was added to the vial to prepare a 0.5 M NaOMe solution. The mixture was stirred at room temperature for 5 h. The reaction was quenched by the addition of 1 N HCl. The methanolysis product was extracted by water-ethyl acetate partitioning after evaporating the MeOH *in vacuo*. After removing the solvent, the methanolysis product (**5**) was purified by HPLC using gradient elution conditions (10%–100% acetonitrile/water over 40 min; UV detection at 280 nm; flow rate: 2 mL/min) and a reversed-phase HPLC column (Kromasil C_18_: 250 × 10 mm, 5 μm). The methanolysis product (**5**) eluted at a retention time of 25 min. The structure of **5** was confirmed by analysis of its ^1^H and 2D NMR spectral data (Figures S22–S24) and ESI mass spectra ([M + H]^+^ *m*/*z* at 339; molecular formula, C_20_H_35_O_4_). The same procedures were repeated for succinilenes B and C (**2** and **3**). The structures of the methanolysis products of **2** and **3** (**6** and **7**) were also assigned based on the analysis of ^1^H and 2D NMR spectral data (Figures S25–S30) and ESI mass spectra (for **6**, [M + H]^+^ *m*/*z* at 339 and molecular formula of C_20_H_33_O_4_; for **7**, [M + H]^+^ *m*/*z* at 337 and molecular formula of C_20_H_33_O_4_).

### 3.5. MTPA Esterification of Succinilenes A–C

The methanolysis products of succinilenes A–C (**5**–**7**) were prepared individually in 40-mL vials (two 1-mg samples for each compound) and dried completely under high vacuum overnight. After adding catalytic amounts of crystalline *N*,*N*-dimethylaminopyridine (DMAP) to each reaction vial, freshly distilled anhydrous pyridine (1 mL) was added under argon gas. The reaction mixtures were stirred at room temperature for 5 min. After 5 min, *R*- and *S*-α-methoxy trifluoromethyl-phenylacetic acid (MTPA) chloride (20 µL) were added, respectively. The reactions were carried out for 3 h at room temperature with stirring. The reactions were quenched by adding 50 µL of MeOH. The reaction products were purified by HPLC using gradient elution conditions of 40% to 100% aqueous acetonitrile over 20 min with a reversed-phase C_18_ column (Kromasil C_18_ : 250 × 10 mm, 5 μm; flow rate: 2 mL/min; UV detection at 280 nm). The tetra-*S*- and -*R*-MTPA esters (**8** and **9**) of **5** eluted at 34.3 and 35.2 min, respectively. The tetra-*S*- and -*R*-MTPA esters (**10** and **11**) of **6** eluted at 33.5 and 34.7 min. The tetra-*S*- and -*R*-MTPA esters (**12** and **13**) of **7** were isolated at 36.1 and 37.3 min. The Δδ*_S_*_-*R*_ values around the stereogenic centers of the MTPA esters were assigned based on the analysis of ^1^H and ^1^H-^1^H COSY NMR spectra.

#### 3.5.1. Tetra *S*-MTPA Ester (**8**) of Methanolysis Product (**5**) of Succinilene A (**1**)

^1^H NMR (600 MHz, pyridine-*d*_5_) δ 7.83–7.81 (m, 4H), 7.78–7.75 (m, 4H), 7.48–7.45 (m, 12H), 6.32–6.17 (m, 4H), 5.73 (m, 1H), 5.67–5.60 (m, 4H), 5.41 (d, *J* = 6.5, 1H), 5.26 (m, 1H), 3.67 (s, 3H), 3.62 (s, 9H), 2.75 (m, 1H), 2.63–2.54 (m, 2H), 2.51–2.46 (m, 2H), 1.76 (s, 3H), 1.72 (m, 1H), 1.58 (m, 1H), 1.32 (d, *J* = 6.5, 3H), 0.93 (d, *J* = 6.5, 3H), 0.88 (t, *J* = 6.5, 3H). The molecular formula of **8** was confirmed as C_60_H_62_F_12_O_12_Na ([M + Na]^+^ at *m*/*z* 1227).

#### 3.5.2. Tetra-*R*-MTPA Ester (**9**) of Methanolysis Product (**5**) of Succinilene A (**1**)

^1^H NMR (600 MHz, pyridine-*d*_5_) δ 7.82–7.81 (m, 4H), 7.79–7.76 (m, 4H), 7.49–7.44 (m, 12H), 6.30–6.22 (m, 4H), 5.75–5.71 (m, 2H), 5.67 (t, *J* = 7.0, 1H), 5.63 (d, *J* = 7.0, 1H), 5.56 (d, *J* = 9.5, 1H), 5.50 (t, *J* = 7.0, 1H ), 5.25 (m, 1H), 3.65 (s, 3H), 3.63 (s, 3H), 3.62 (s, 3H), 3.59 (s, 3H), 2.77 (m, 1H), 2.68 (m, 1H) 2.64–2.61 (m, 2H), 2.55 (m, 1H), 1.80–1.74 (m, 2H), 1.63 (s, 3H), 1.25 (d, *J* = 6.5, 3H), 1.00 (d, *J* = 6.5, 3H), 0.86 (t, *J* = 6.5, 3H). The molecular formula of **9** was confirmed as C_60_H_62_F_12_O_12_Na ([M + Na]^+^ at *m*/*z* 1227).

#### 3.5.3. Tetra-*S*-MTPA Ester (**10**) of Methanolysis Product (**6**) of Succinilene B (**2**)

^1^H NMR (600 MHz, pyridine-*d*_5_) δ 7.81–7.77 (m, 8H), 7.48–7.45 (m, 12H), 6.32–6.25 (m, 3H), 6.19 (dd, *J* = 14.5, 10.5, 1H), 5.80–5.75 (br, 3H), 5.65 (m, 1H), 5.64 (m, 1H), 5.61 (m, 1H), 5.58 (br, 1H), 3.67 (s, 6H), 3.61 (s, 6H), 2.74 (br, 1H), 2.68–2.66 (m, 2H), 2.58 (m, 1H), 2.49 (m, 1H), 1.73 (m, 3H), 1.67–1.62 (m, 2H), 0.94 (d, *J* = 6.5, 3H), 0.89 (d, *J* = 6.5, 3H), 0.86 (t, *J* = 7.5, 3H). The molecular formula of **10** was confirmed as C_60_H_62_F_12_O_12_Na ([M + Na]^+^ at *m*/*z* 1227).

#### 3.5.4. Tetra-*R*-MTPA Ester (**11**) of Methanolysis Product (**6**) of Succinilene B (**2**)

^1^H NMR (600 MHz, pyridine-*d*_5_) δ 7.75–7.73 (m, 8H), 7.46–7.44 (m, 12H), 6.30–6.29 (m, 2H), 6.24 (m, 1H), 6.17 (m, 1H), 5.76–5.72 (br, 3H), 5.67 (m, 1H), 5.63 (m, 1H), 5.58 (br, 1H), 5.56 (d, *J* = 9.5, 1H), 3.66 (s, 3H), 3.63 (s, 6H), 3.58 (s, 3H), 2.77 (br, 1H), 2.67 (m, 1H) 2.56–2.54 (m, 2H), 2.45 (m, 1H), 1.76 (m, 2H), 1.68 (s, 3H), 0.98 (d, *J* = 6.5, 3H), 0.90 (t, *J* = 7.5, 3H), 0.84 (d, *J* = 6.5, 3H). The molecular formula of **11** was confirmed as C_60_H_62_F_12_O_12_Na ([M + Na]^+^ at *m*/*z* 1227).

#### 3.5.5. Tetra-*S*-MTPA Ester (**12**) of Methanolysis Product (**7**) of Succinilene C (**3**)

^1^H NMR (600 MHz, pyridine-*d*_5_) δ 7.81–7.78 (m, 6H), 7.50–7.47 (m, 9H), 6.25–6.15 (m, 3H), 6.02 (dd, *J* = 15.0, 9.5, 1H), 5.73 (m, 1H), 5.67–5.62 (m, 2H), 5.57 (m, 1H), 5.55 (d, *J* = 9.5, 1H), 5.28 (m, 1H), 3.67 (s, 3H), 3.65 (s, 3H), 3.63 (s, 3H), 2.92 (m, 1H), 2.82–2.73 (m, 2H), 2.69 (m, 1H), 2.58–2.50 (m, 3H), 1.69 (s, 3H), 1.13 (d, *J* = 6.5, 3H), 1.05 (t, *J* = 7.5, 3H), 0.89 (d, *J* = 6.5, 3H). The molecular formula of **12** was confirmed as C_50_H_53_F_9_O_10_Na ([M + Na]^+^ at *m*/*z* 1007).

#### 3.5.6. Tetra-*R*-MTPA Ester (**13**) of Methanolysis Product (**7**) of Succinilene C (**3**)

^1^H NMR (600 MHz, pyridine-*d*_5_) δ 7.82–7.80 (m, 6H), 7.51–7.48 (m, 9H), 6.23–6.15 (m, 4H), 5.74 (m, 1H), 5.66–5.60 (m, 2H), 5.58 (t, *J* = 6.5, 1H), 5.42 (m, 1H), 5.26 (m, 1H), 3.69 (s, 3H), 3.65 (s, 3H), 3.63 (s, 3H), 2.91 (m, 1H), 2.84–2.75 (m, 2H), 2.62 (m, 1H), 2.57–2.51 (m, 3H), 1.65 (s, 3H), 1.18 (d, *J* = 6.5, 3H), 1.06 (t, *J* = 7.5, 3H), 0.85 (d, *J* = 6.5, 3H). The molecular formula of **13** was confirmed as C_50_H_53_F_9_O_10_Na ([M + Na]^+^ at *m*/*z* 1007).

### 3.6. Evaluation of Antiproliferative Activity

The effects of succinilenes A–D (**1**–**4**) on cell proliferation were evaluated by the sulforhodamine B (SRB) cellular protein-staining assay, with slight modifications. Briefly, A549 (lung cancer), SNU638 (gastric cancer), and HCT116 (colon cancer) cells (1 × 10^4^ cells in 190 µL of complete RPMI 1640 medium) were seeded in a 96-well plate with various concentrations of **1**–**4** and incubated at 37 °C in a humidified atmosphere containing 5% CO_2_. After 72 h of treatment with succinilenes A–D (**1**–**4**), the cells were fixed with a 10% TCA solution for 1 h, and cellular proteins were stained with 0.4% SRB in a 1% acetic acid solution. The stained cells were dissolved in 10 mM Tris buffer (pH 10.0). The effects of **1**–**4** on cell viability were calculated as percentages relative to the solvent-treated control. The IC_50_ values were calculated using nonlinear regression analysis (percent survival versus concentration).

### 3.7. Evaluation of i-NOS Assay

#### 3.7.1. Materials

Dulbecco’s modified Eagle’s medium (DMEM), fetal bovine serum (FBS), sodium pyruvate, l-glutamine, antibiotics-antimycotics solution, and trypsin-EDTA were purchased from Invitrogen Co. (Grand Island, NY, USA). Lipopolysaccharide (LPS, *Escherichia coli* 0111:B4), 3-(4,5-dimethylthiazol-2-yl)-2,5-diphenyltetrazolium bromide (MTT), and other chemicals were purchased from Sigma (St. Louis, MO, USA).

#### 3.7.2. Cell Culture

Mouse macrophage RAW 264.7 cells, provided from the American Type Culture Collection (ATCC, Rockville, MD, USA), were cultured in DMEM supplemented with 10% heat-inactivated fetal bovine serum (FBS) and antibiotics-antimycotics (PSF; 100 units/mL penicillin G sodium, 100 μg/mL streptomycin, and 250 ng/mL amphotericin B). Cells was incubated in humidified atmosphere containing 5% CO_2_ at 37 °C.

#### 3.7.3. Nitrite Assay

To evaluate the inhibitory activity of succinilenes A–D (**1**–**4**) on LPS-induced NO production, RAW 264.7 cells were cultured in 10% FBS-DMEM without phenol red, plated in 24-well plates (3 × 10^5^ cells/mL), and incubated for 24 h. Cells were washed with PBS, fresh medium was added, and the cells were incubated with 1 μg/mL LPS in the presence or absence of test compounds. After additional 20 h of incubation, the media were collected and analyzed for nitrite accumulation as an indicator of NO production by the Griess reaction. Briefly, 180 μL of Griess reagents (0.1% *N*-(1-naphthyl)ethylenediamine dihydrochloride in H_2_O and 1% sulfanilamide in 5% H_3_PO_4_) was added to 100 μL of each supernatant from LPS or sample-treated cells in 96-well plates. The absorbance was measured at 540 nm, and nitrite concentration was determined by comparison with a sodium nitrite standard curve. Percent inhibition was expressed as [1 − (NO level of test samples/NO levels of vehicle-treated control)] × 100.

#### 3.7.4. Statistical Analysis

Data are presented as the means ± SD for the indicated number of independently performed experiments. Statistical significance (*p* < 0.01) was assessed by a one-way analysis of variation (ANOVA) coupled with the Dunnett’s *t*-test.

### 3.8. Computer-Aided NMR Spectral Analysis

The PERCH NMR software package (ver. 2013.1, PERCH Solutions Ltd., Kuopio, Finland) was used for HiFSA, as previously described [2,23,24,25]. The final optimized NMR parameters of compounds **1**–**4** are provided as PERCH parameter text files (*.pms) (Figures S44–S47).

## 4. Conclusions

Succinilenes A–D (**1**–**4**) were discovered from a marine-derived *Streptomyces* sp. through chemical profiling of a marine actinomycete strain collection. Spectroscopic analysis of **1**–**4** was used to assign their structures as new polyene polyol compounds, each bearing a conjugated triene moiety. These compounds were biologically evaluated for inhibitory effects on LPS-induced NO production, indicating their anti-inflammatory significance. Succinilenes A–C, commonly incorporating a succinic acid moiety, displayed significant inhibitory effects on NO production whereas succinilene D without succinic acid did not inhibit NO production, thus proposing that the succinic moiety is important for the inhibitory effects on LPS-induced NO production. Microbial metabolites having succinic acid are not common, but a few microbial polyketide compounds are known to bear succinic acid in their structures, such as macrolactin T from the marine bacterium *Bacillus marinus* [26], reveromycin A from *Streptomyces* sp. [27], and macrocyclic lactone A26771B from the fungus *Penicillium turbatum* [28]. These succinic acid-bearing compounds have not been tested for the inhibitory activities on LPS-induced NO production.

Since the chemical shifts of the olefinic protons belonging to the triene moieties and, thus, coupling with each other, are close enough to generate second-order peaks, the triene geometries of the succinilenes could not be determined using conventional methods. Our application of the spin simulation of QM-HiFSA enabled us to calculate the ^1^H-^1^H coupling constants of these double bond protons and successfully determine the triene geometries. These results suggest that the QM-HiFSA technique is a useful tool for the detailed structural elucidation of complicated natural products displaying highly overlapped ^1^H NMR spectra.

## Figures and Tables

**Table 1:** NMR data for **1** and **2** in pyridine-*d*_5_.

Position	1			2		
	δ_H_^a^	Mult (*J* in Hz)	δ_C_^b^	δ_H_^c^	Mult (*J* in Hz)	δ_C_^d^
**1**	5.0898	dq (6.35, 5.50)	74.1, d	5.0882	dq (6.35, 5.75)	74.1, d
**1-Me**	1.1907	d (6.35)	17.2, q	1.1862	d (6.35)	17.2, q
**2**	2.7397	ddq (9.74, 6.82, 5.50)	37.1, d	2.7356	ddq (9.60, 6.76, 5.75)	37.2, d
**2-Me**	0.9973	d (6.82)	16.6, q	0.9916	d (6.76)	16.7, q
**3**	5.5426	d (9.74)	127.2, d	5.5359	d (9.60)	127.2, d
**4**	–	–	140.1, s	–	–	140.1, s
**4-Me**	1.8616	s	12.2, q	1.8618	s	12.1, q
**5**	4.3745	dd (7.04, 6.04)	76.8, d	4.3721	dd (6.69, 6.34)	76.9, d
**6**	2.6519	ddd (−14.00, 7.30, 7.04)	39.8, t	2.6498	ddd (−14.11, 7.47, 6.69)	39.8, t
	2.5631	ddd (−14.00, 7.50, 6.04)		2.5587	ddd (−14.11, 7.10, 6.34)	
**7**	5.9506	ddd (14.65, 7.50, 7.30)	132.0, d	5.9395	ddd (14.81, 7.47, 7.10)	131.9, d
**8**	6.2885	dd (14.65, 10.77)	132.5, d	6.2814	dd (14.81, 10.73)	132.6, d
**9**	6.2929	dd (14.03, 10.77)	131.6, d	6.2773	dd (14.79, 10.73)	131.6, d
**10**	6.3158	dd (14.03, 10.49)	131.7, d	6.3179	dd (14.79, 10.53)	131.7, d
**11**	6.3664	dd (15.19, 10.49)	132.6, d	6.3913	dd (15.19, 10.53)	132.7, d
**12**	6.1280	ddd (15.19, 7.50, 7.12)	132.8, d	6.2145	ddd (15.19, 7.45, 7.24)	133.0, d
**13**	2.7253	ddd (−14.36, 7.50, 4.66)	38.0, t	2.8829	ddd (−14.13, 7.24, 3.36)	37.5, t
	2.6745	ddd (−14.36, 8.02, 7.12)		2.7481	ddd (−14.13, 8.12, 7.45)	
**14**	3.9176	ddd (8.02, 4.66, 4.33)	74.2, d	4.0324	ddd (8.12, 5.84, 3.36)	74.9, d
**15**	3.7579	ddd (8.68, 4.33, 3.88)	75.5, d	3.8871	ddd (8.72, 5.84, 2.69)	76.1, d
**16**	1.8578	ddq (−13.87, 7.38, 3.88)	27.5, t	2.0541	ddq (−13.61, 7.36, 2.69)	26.5, t
	1.8105	ddq (−13.87, 8.68, 7.49)		1.8504	ddq (−13.61, 8.72, 7.42)	
**17**	1.1619	dd (7.49, 7.38)	10.9, q	1.2234	dd (7.42, 7.36)	10.9, q
**1′**	–	–	172.7, s	–	–	173.0, s
**2′**	2.8516	ddd (−11.48, 7.29, 6.04)	30.3, t	2.8496	ddd (−18.10, 7.13, 6.33)	30.6, t
	2.8436	ddd (−11.48, 7.54, 5.82)		2.8496	ddd (−18.10, 8.56, 4.83)	
**3′**	2.9046	ddd (−16.39, 7.29, 5.82)	30.4, t	2.9078	ddd (−15.38, 8.56, 7.13)	30.8, t
	2.8954	ddd (−16.39, 7.54, 6.04)		2.9022	ddd (−15.38, 6.33, 4.83)	
**4′**	–	–	175.7, s	–	–	176.2, s

^a^ 900 MHz; ^b^ 150 MHz; ^c^ 600 MHz; ^d^ 150 MHz. The *δ*_H_ (in ppm) and *J* (in Hz) values were determined by HiFSA.

**Table 2:** NMR data for **3** and **4** in pyridine-*d*_5_.

Position	3			4		
	δ_H_^a^	Mult (*J* in Hz)	δ_C_^b^	δ_H_^a^	Mult (*J* in Hz)	δ_C_^b^
**1**	5.1041	dq (6.33, 5.27)	74.2, d	3.9666	ddq (6.23, 5.00, 4.60)	70.8, d
**1-Me**	1.1944	d (6.33)	17.3, q	1.3158	d (6.23)	21.0, q
**2**	2.7403	ddq (9.97, 6.86, 5.27)	37.2, d	2.6988	ddq (9.62, 6.85, 5.00)	39.6, d
**2-Me**	1.0069	d (6.86)	16.6, q	1.1905	d (6.85)	17.0, q
**3**	5.5580	d (9.97)	127.4, d	5.8422	d (9.62)	128.6, d
**4**	–	–	140.3, s	–	–	138.8, s
**4-Me**	1.8616	s	12.4, q	1.8782	s	12.1, q
**5**	4.3767	dd (7.10, 6.06)	76.9, d	4.4482	ddd (7.07, 5.51, 3.83)	77.2, d
**6**	2.6443	ddd (−13.79, 6.93, 6.06)	39.8, t	2.6890	ddd (−13.68, 7.07, 7.00)	39.9, t
	2.5606	ddd (−13.79, 7.12, 7.10)		2.6130	ddd (−13.68, 7.99, 5.51)	
**7**	5.9652	ddd (14.66, 7.12, 6.93)	132.6, d	5.9948	ddd (14.80, 7.99, 7.00)	132.1, d
**8**	6.2708	dd (14.66, 10.63)	132.5, d	6.2974	dd (14.80, 10.62)	132.7, d
**9**	6.2902	dd (15.05, 10.63)	132.4, d	6.2738	dd (14.86, 10.62)	131.8, d
**10**	6.2414	dd (15.05, 10.72)	131.2, d	6.3186	dd (14.86, 10.55)	132.1, d
**11**	6.3255	dd (15.14, 10.72)	133.7, d	6.3706	dd (15.21, 10.55)	133.0, d
**12**	5.9778	ddd (15.14, 7.41, 7.00)	129.8, d	6.2091	ddd (15.21, 7.38, 7.16)	133.2, d
**13**	2.7803	ddd (−13.49, 7.27, 7.00)	38.2, t	2.8870	ddd (−14.32, 7.38, 3.45)	37.6, t
	2.6591	ddd (−13.49, 7.41, 4.88)		2.7460	ddd (−14.32, 8.46, 7.16)	
**14**	4.4922	dd (7.27, 4.88)	77.2, d	4.0282	dddd (8.46, 5.85, 5.74, 3.45)	75.0, d
**15**	–	–	214.0, s	3.8872	dddd (8.87, 5.85, 5.90, 3.10)	76.2, d
**16**	2.7549	dq (−14.89, 7.05)	31.7, t	2.0620	ddq (−13.56, 7.35, 3.10)	26.5, t
	2.7206	dq (−14.89, 7.45)		1.8533	ddq (−13.56, 8.87, 7.37)	
**17**	1.0890	dd (7.45, 7.05)	7.8, q	1.2266	dd (7.37, 7.35)	10.8, q
**1′**	–	–	172.7, s			
**2′**	2.8525	ddd (−16.88, 7.01, 6.26)	30.3, t			
	2.8525	ddd (−16.88, 8.79, 4.47)				
**3′**	2.9172	ddd (−16.89, 8.79, 7.01)	30.0, t			
	2.9060	ddd (−16.89, 6.26, 4.47)				
**4′**	–	–	175.3, s			
OH-1				5.7021	d (4.60)	
OH-5				6.4159	d (3.83)	
OH-14				6.1408	d (5.74)	
OH-15				6.0448	d (5.90)	

^a^ 600 MHz; ^b^ 150 MHz. The *δ*_H_ (in ppm) and *J* (in Hz) values were determined by HiFSA.

## Supplementary Materials

The following are available online at www.mdpi.com/1660-3397/15/2/38/s1. Figures S1–S6: ^1^H, ^13^C, COSY, HSQC, HMBC, and HETLOC NMR data of **1** in pyridine-*d*_5_, Figures S7–S11: ^1^H, ^13^C, COSY, HSQC, and HMBC NMR data of **2** in pyridine-*d*_5_, Figures S12–S16: ^1^H, ^13^C, COSY, HSQC, and HMBC NMR data of **3** in pyridine-*d*_5_, Figures S17–S21: ^1^H, ^13^C, COSY, HSQC, and HMBC NMR data of **4** in pyridine-*d*_5_, Figures S22–S24: ^1^H, COSY, and HSQC NMR data of methanolysis product of succinilene A (**5**) in pyridine-*d*_5_, Figures S25–S27: ^1^H, ^13^C, COSY, and HSQC NMR data of methanolysis product of succinilene B (**6**) in pyridine-*d*_5_, Figures S28–S30: ^1^H, ^13^C, COSY, and HSQC NMR data of methanolysis product of succinilene C (**7**) in pyridine-*d*_5_, Figures S31 and S32: ^1^H and COSY NMR data of *S*-MTPA ester (**8**) of **5** in pyridine-*d*_5_, Figures S33 and S34: ^1^H and COSY NMR data of *R*-MTPA ester (**9**) of **5** in pyridine-*d*_5_, Figures S35 and S36: ^1^H and COSY NMR data of *S*-MTPA ester (**10**) of **6** in pyridine-*d*_5_, Figures S37 and S38: ^1^H and COSY NMR data of *R*-MTPA ester (**11**) of **6** in pyridine-*d*_5_, Figures S39 and S40: ^1^H and COSY NMR data of *S*-MTPA ester (**12**) of **7** in pyridine-*d*_5_, Figures S41 and S42: ^1^H and COSY NMR data of *R*-MTPA ester (**13**) of **7** in pyridine-*d*_5_, Figure S43: ECD spectra of **5**, **6**, and succinilene D (**4**), Figures S44–S47: The ^1^H NMR fingerprint of **1**–**4** (PERCH .pms file format).
