# Supplementary material for: QM-HiFSA-Aided Structure Determination of Succinilenes A–D, New Triene Polyols from a Marine-Derived Streptomyces sp
Source: Mar Drugs. 2017 Feb 14;15(2):38. doi: 10.3390/md15020038 (PMC5334618; doi:10.3390/md15020038)
Supplement: Supplementary file 1 [file marinedrugs-15-00038-s001.docx]

Supplementary Materials: QM-HiFSA-Aided Structure Determination of Succinilenes A–D,
New Triene Polyols from a Marine-Derived *Streptomyces* sp.

Munhyung Bae, So Hyun Park, Yun Kwon, Sang Kook Lee, Jongheon Shin, Joo-Won Nam
and Dong-Chan Oh


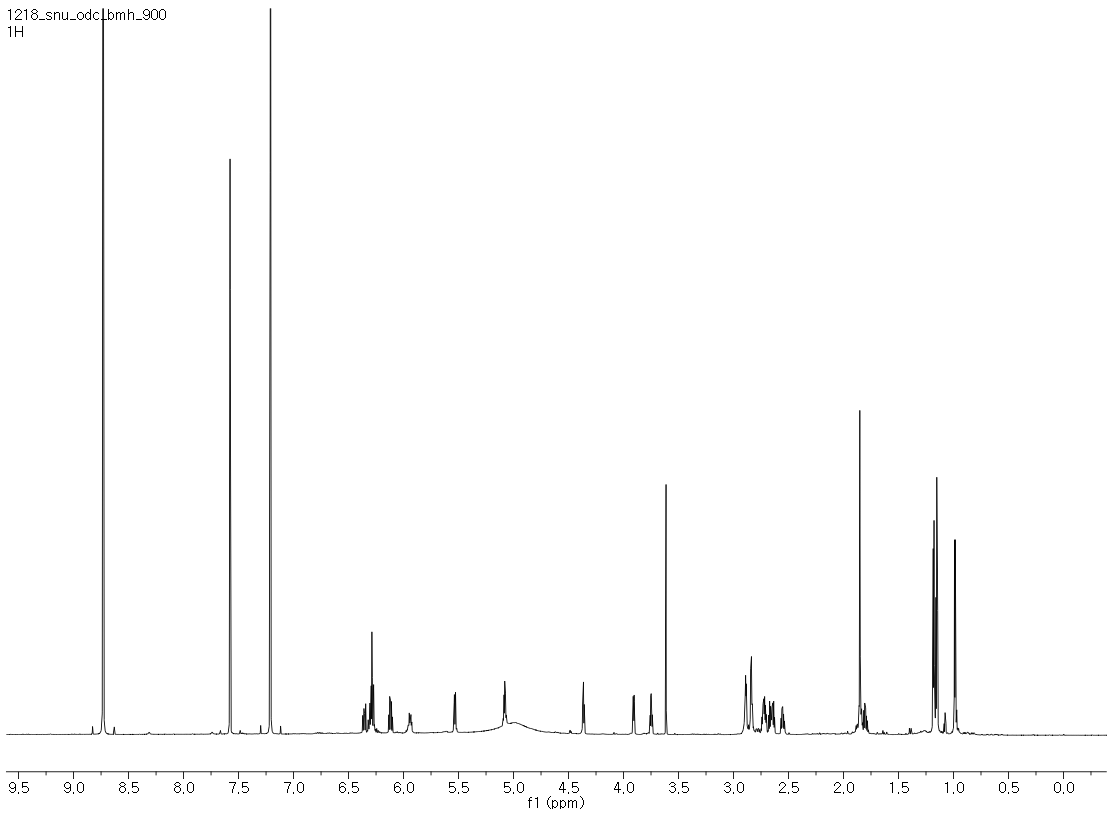


**Figure S1.** ^1^H NMR spectrum (900 MHz) of succinilene A (**1**) in pyridine-*d*_5_.


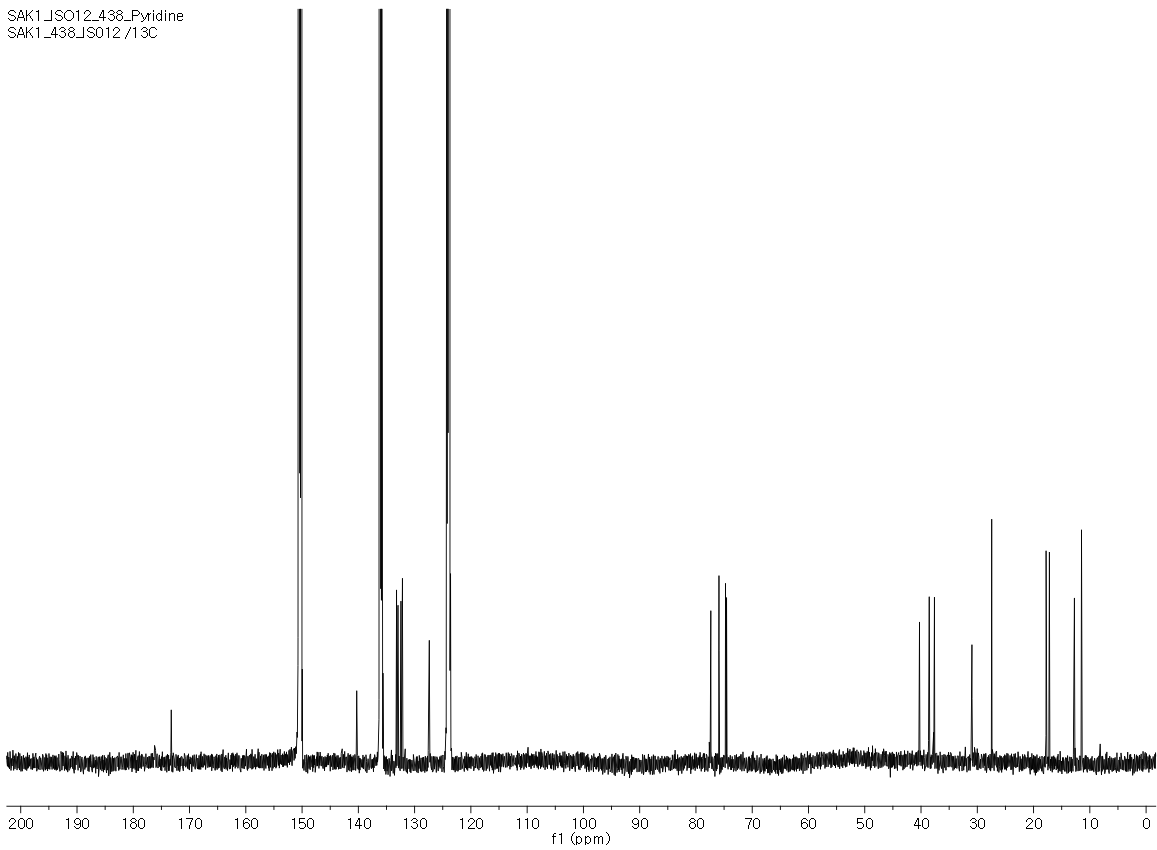


**Figure S2.** ^13^C NMR spectrum (150 MHz) of succinilene A (**1**) in pyridine-*d*_5_.


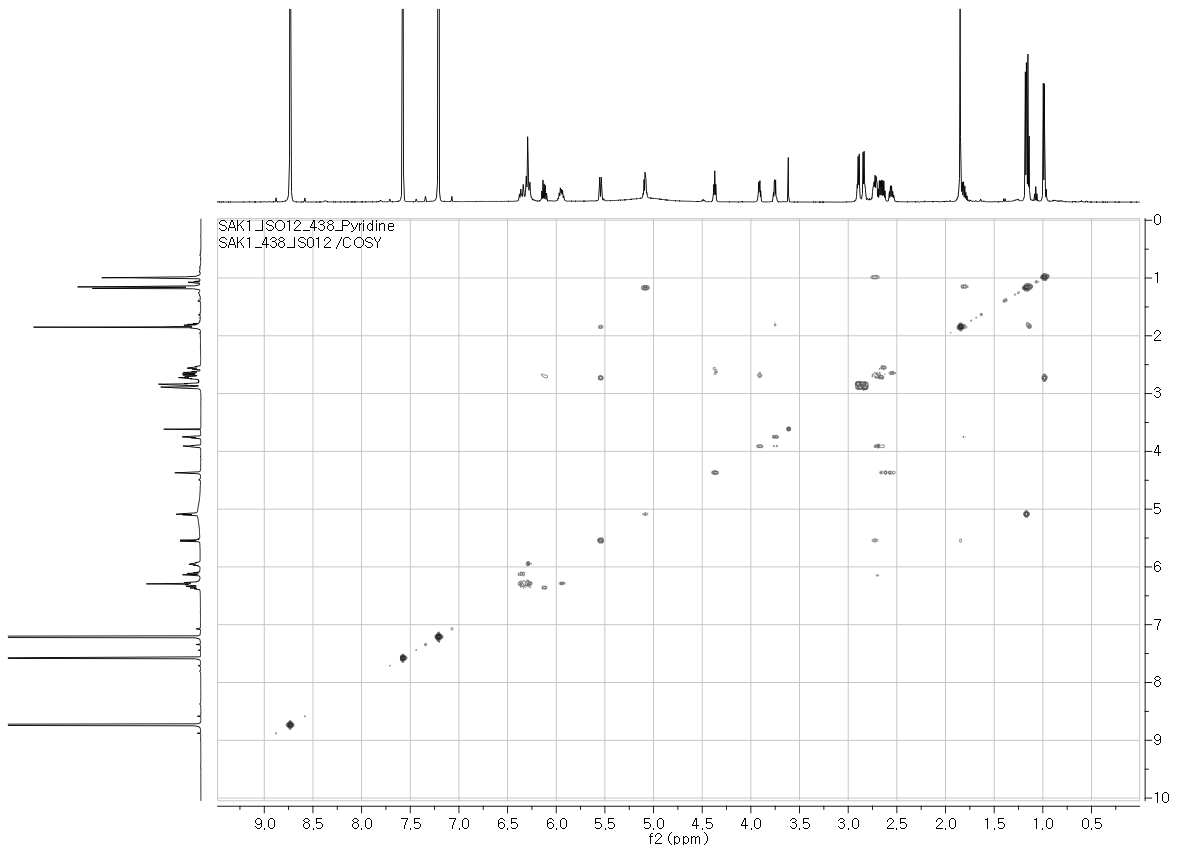


**Figure S3.** COSY spectrum (600 MHz) of succinilene A (**1**) in pyridine-*d*_5_.


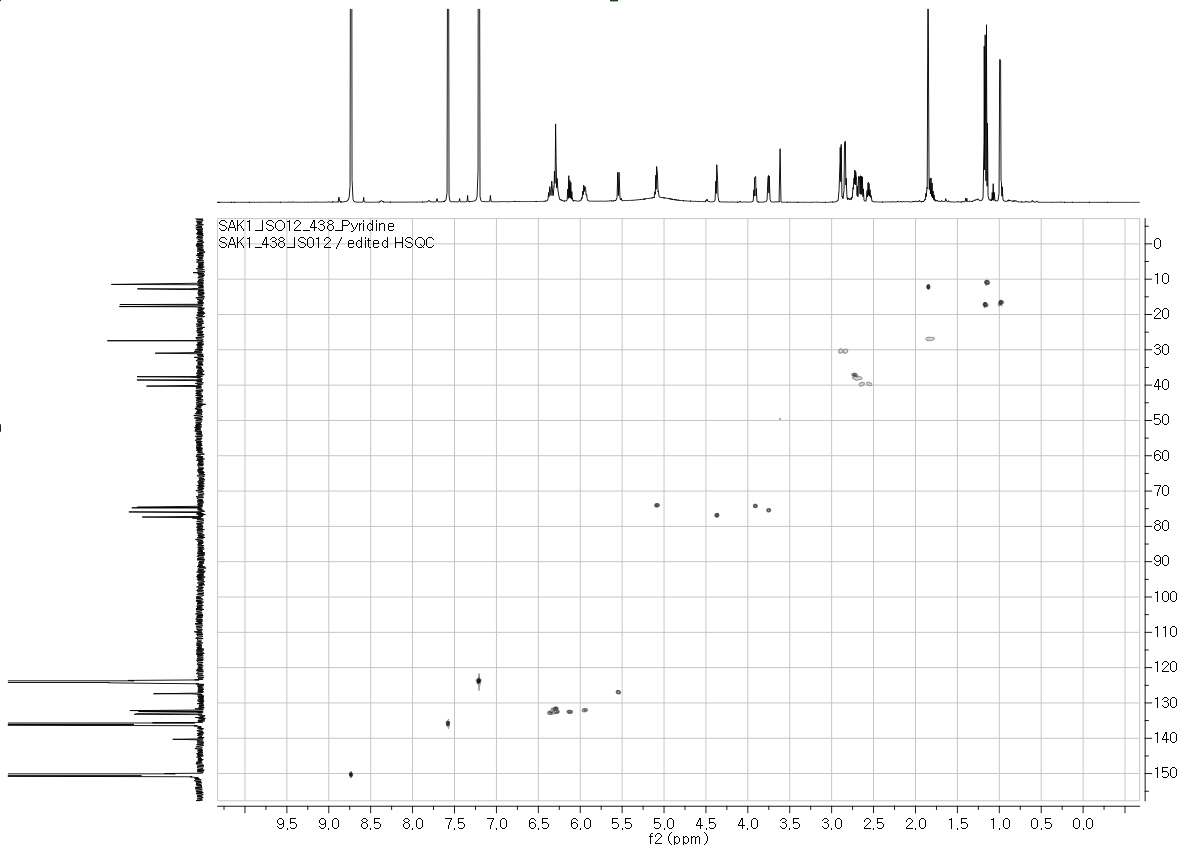


**Figure S4.** HSQC spectrum (600 MHz) of succinilene A (**1**) in pyridine-*d*_5_.


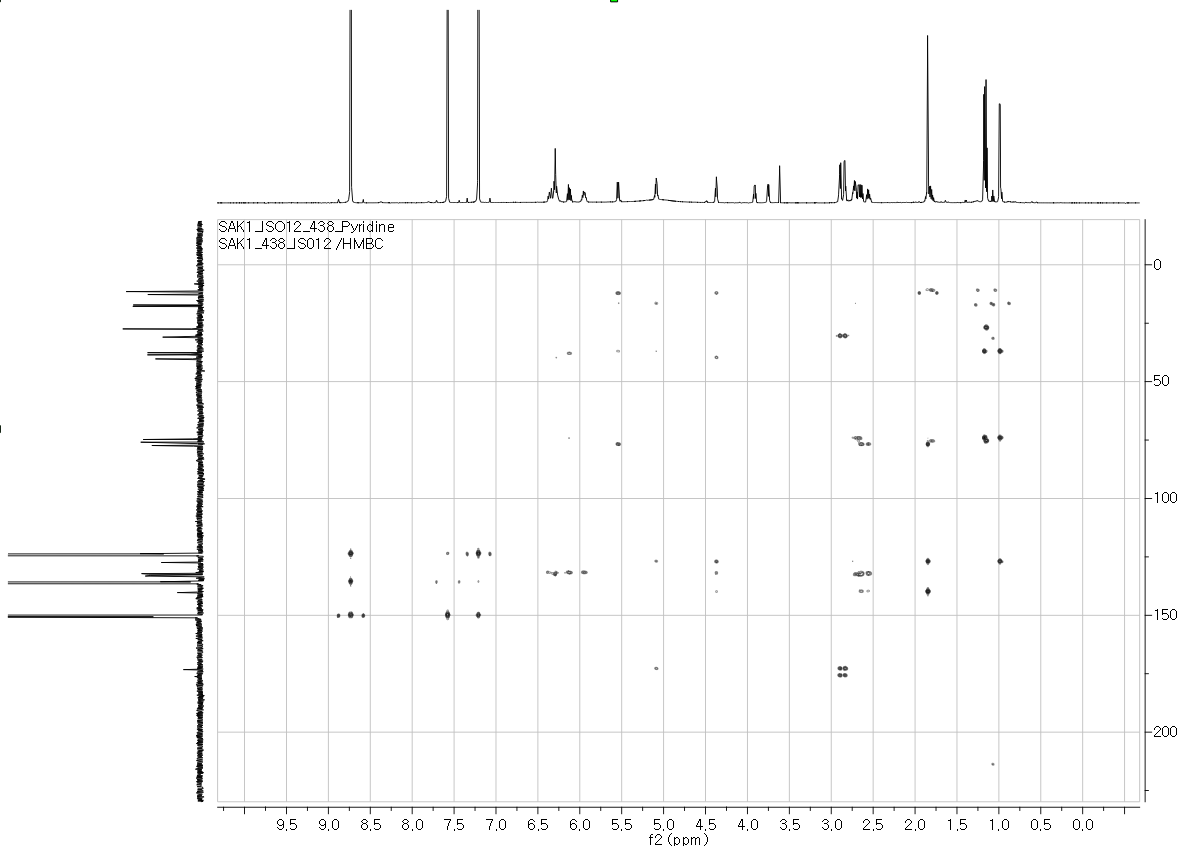


**Figure S5.** HMBC spectrum (600 MHz) of succinilene A (**1**) in pyridine-*d*_5_.


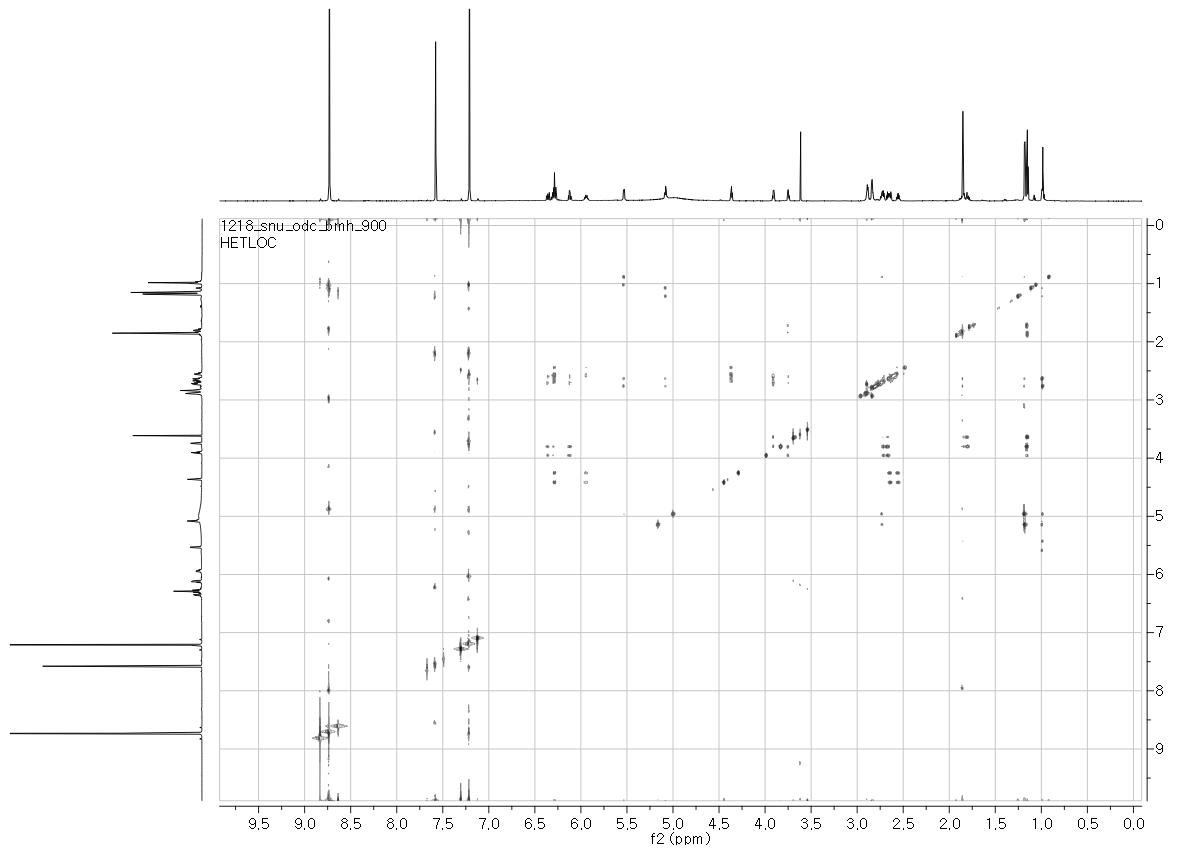


**Figure S6.** HETLOC spectrum (600 MHz) of succinilene A (**1**) in pyridine-*d*_5_.


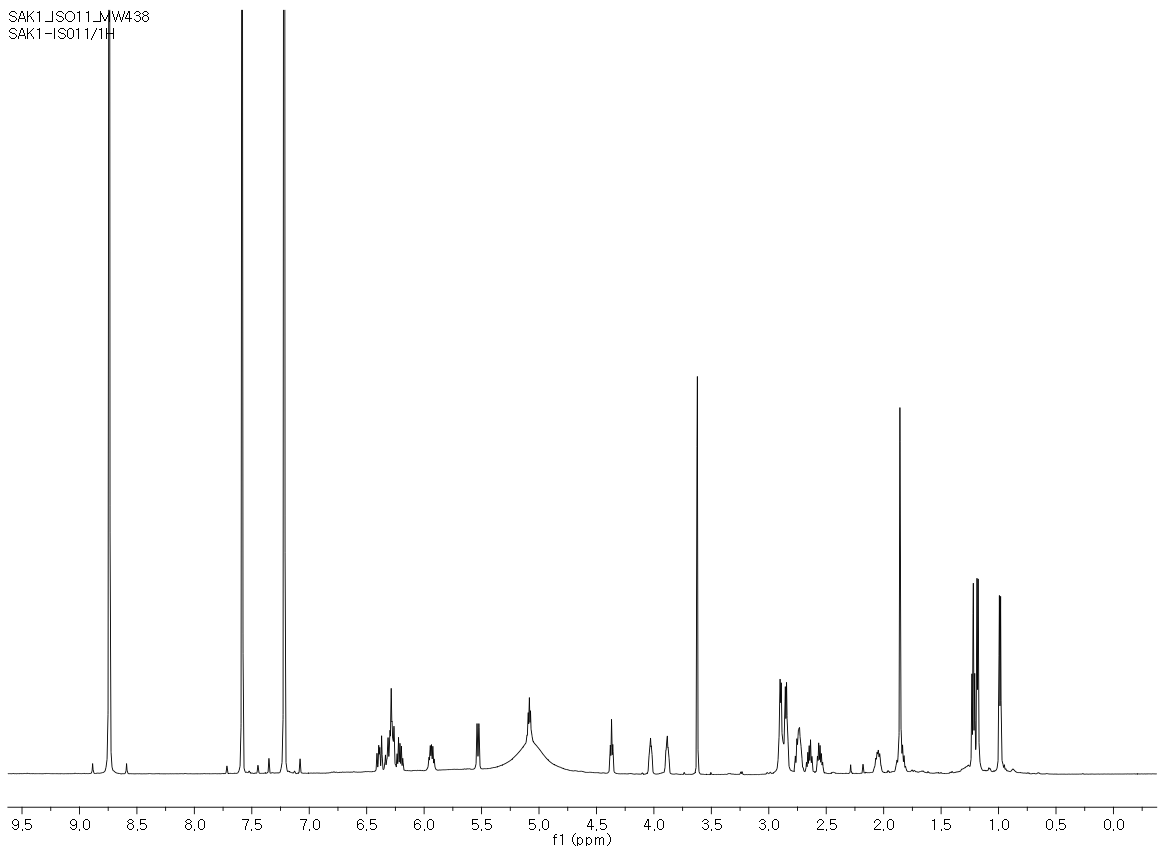


**Figure S7.** ^1^H NMR spectrum (600 MHz) of succinilene B (**2**) in pyridine-*d*_5_.


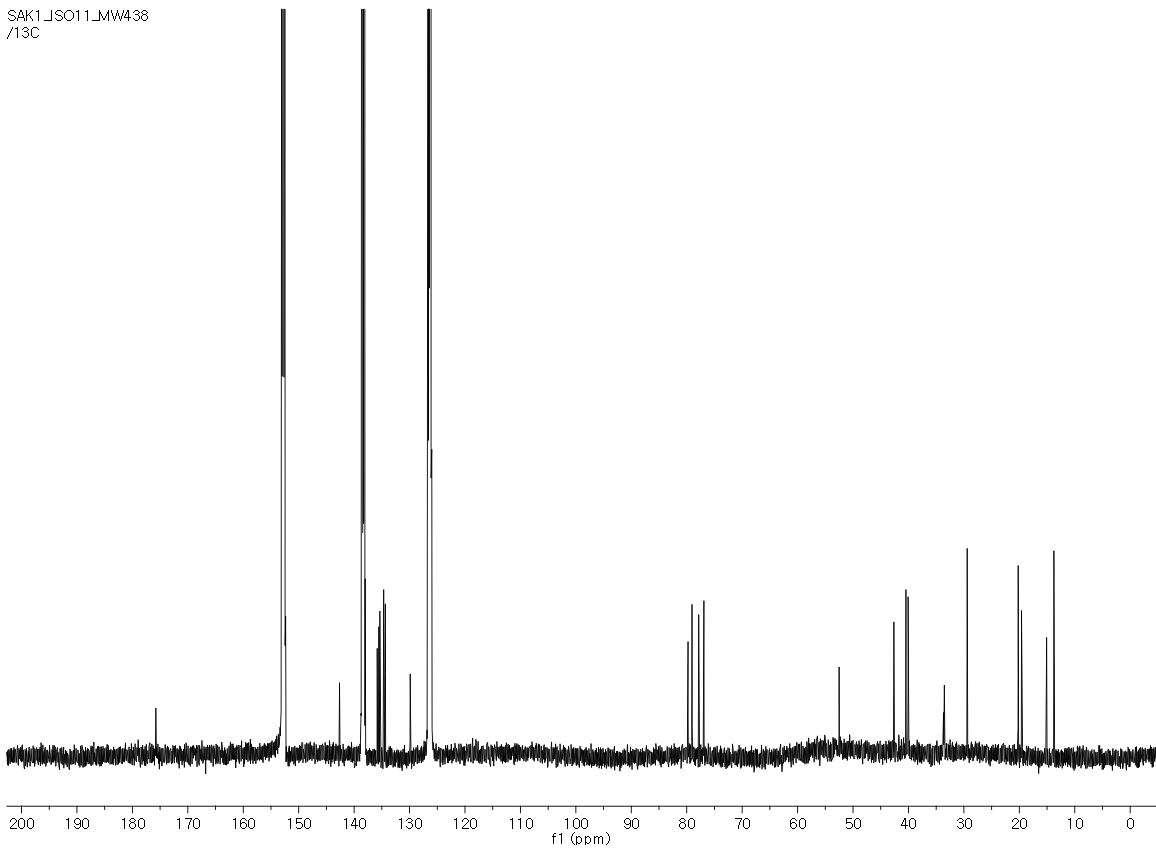


**Figure S8.** ^13^C NMR spectrum (150 MHz) of succinilene B (**2**) in pyridine-*d*_5_.


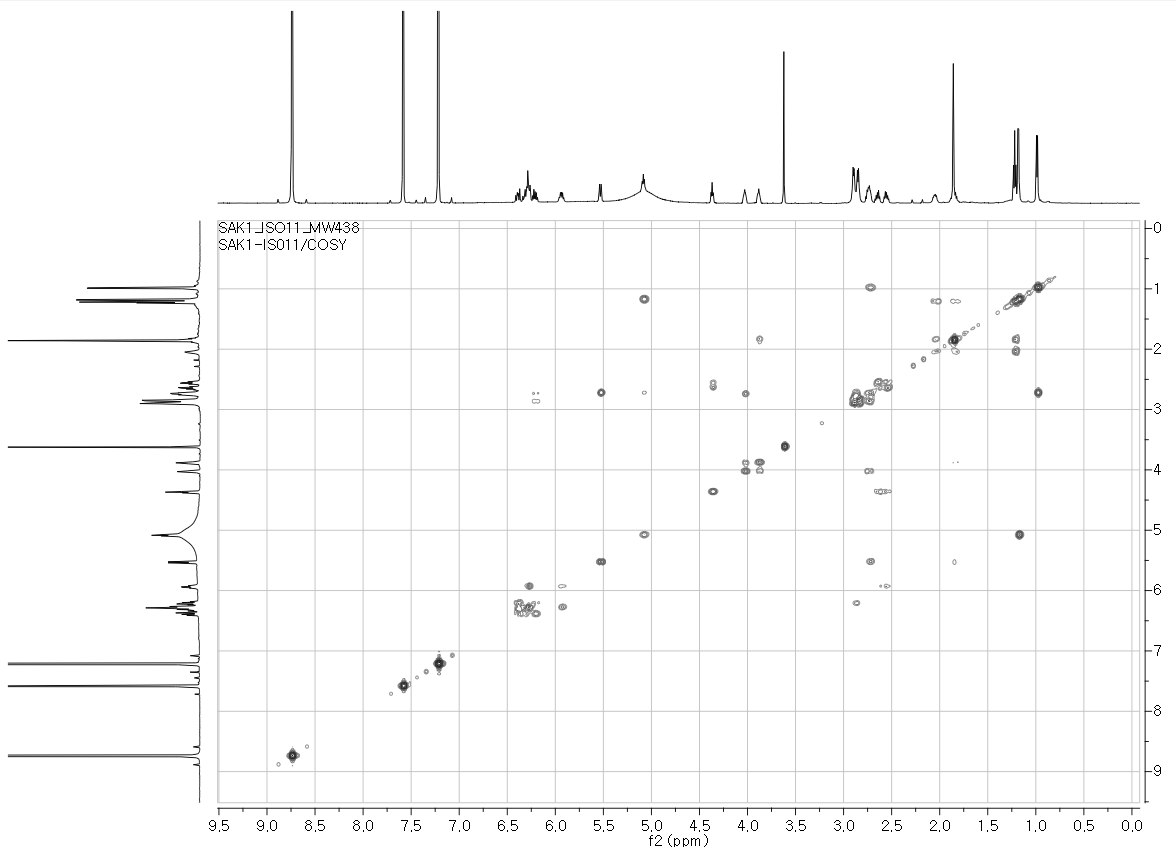


**Figure S9.** COSY spectrum (600 MHz) of succinilene B (**2**) in pyridine-*d*_5_.


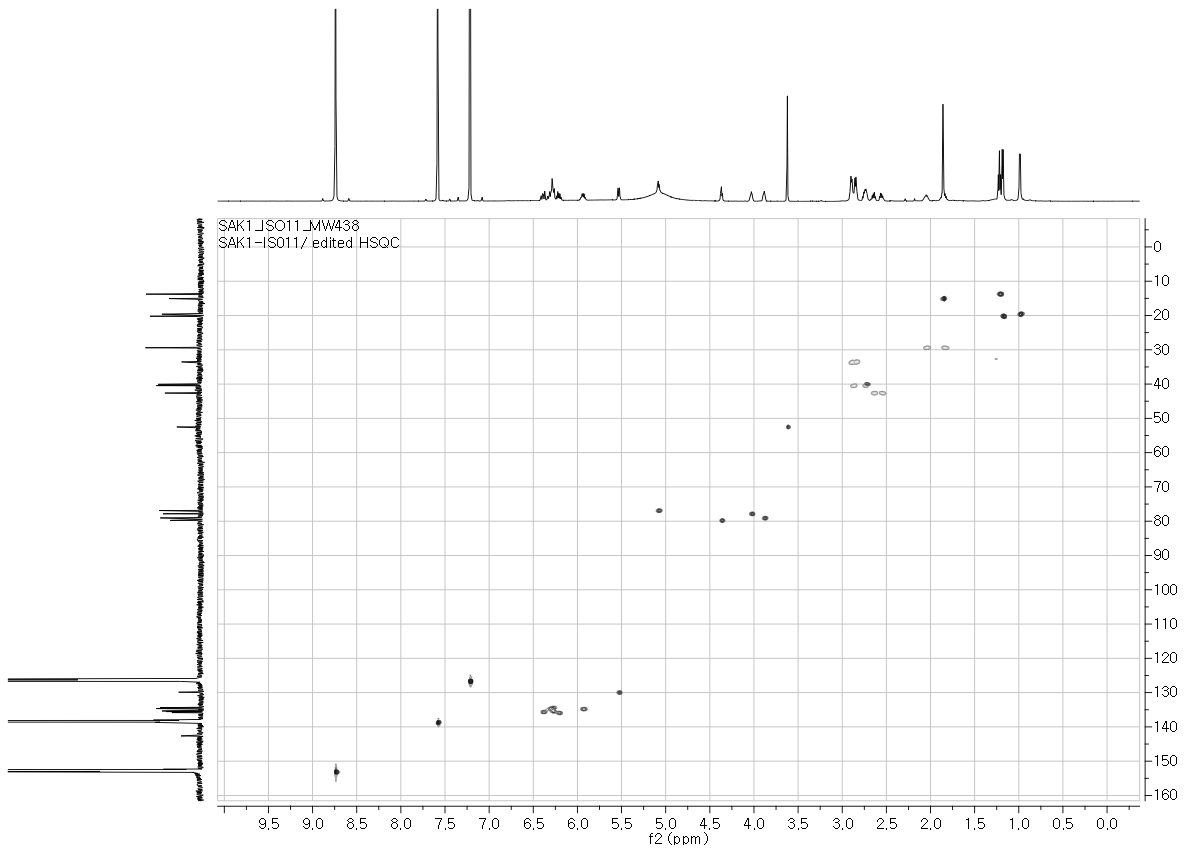


**Figure S10.** HSQC spectrum (600 MHz) of succinilene B (**2**) in pyridine-*d*_5_.


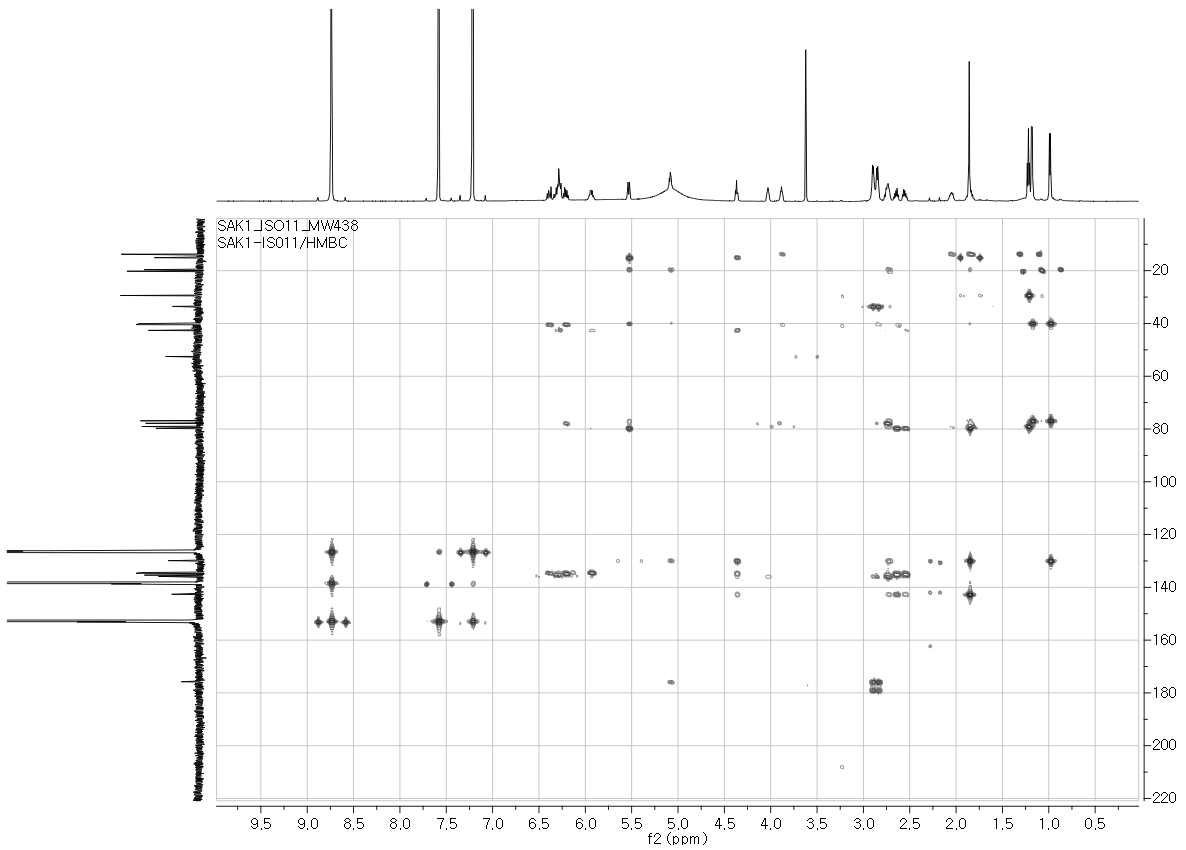


**Figure S11.** HMBC spectrum (600 MHz) of succinilene B (**2**) in pyridine-*d*_5_.


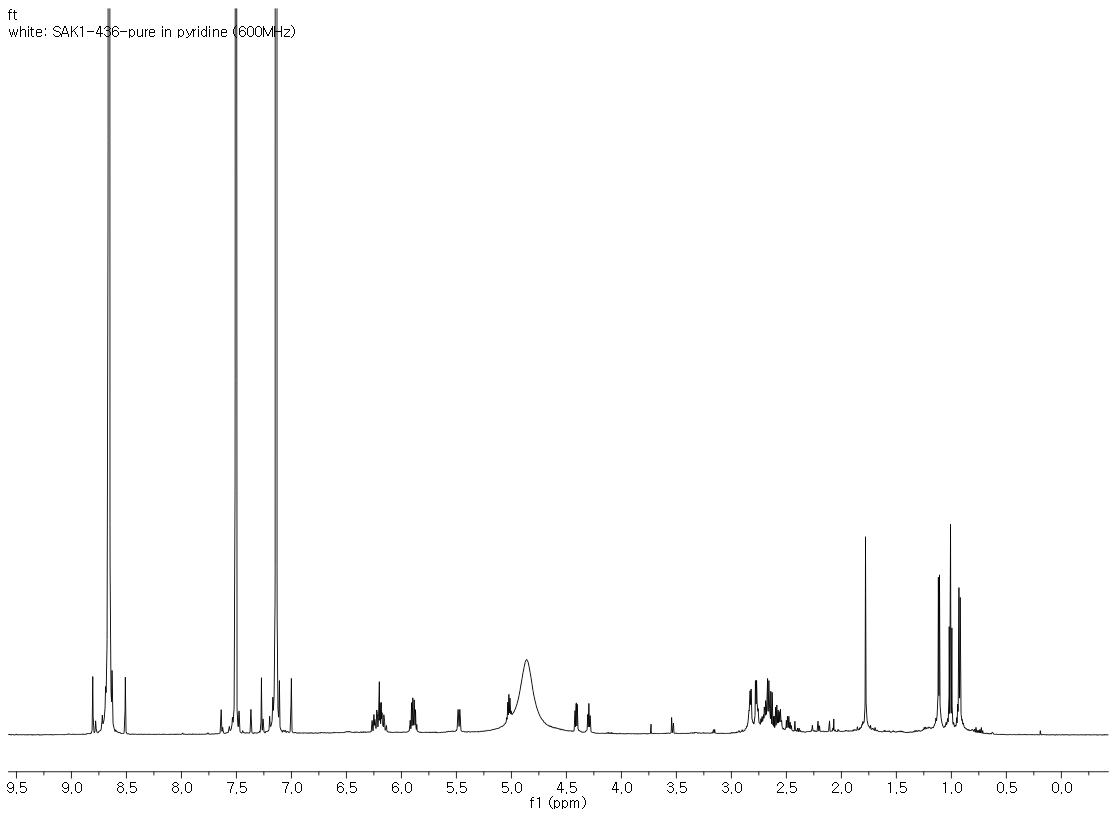


**Figure S12.** ^1^H NMR spectrum (600 MHz) of succinilene C (**3**) in pyridine-*d*_5_.


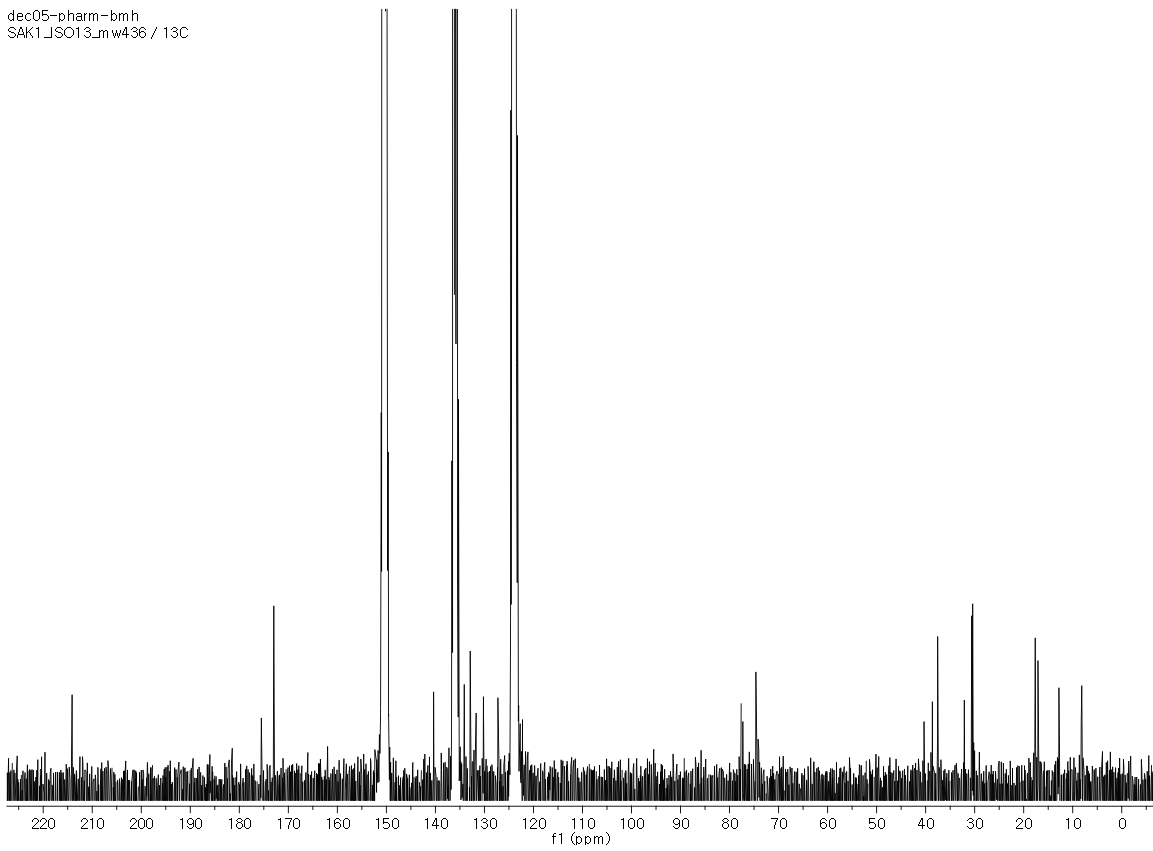


**Figure S13.** ^13^C NMR spectrum (150 MHz) of succinilene C (**3**) in pyridine-*d*_5_.


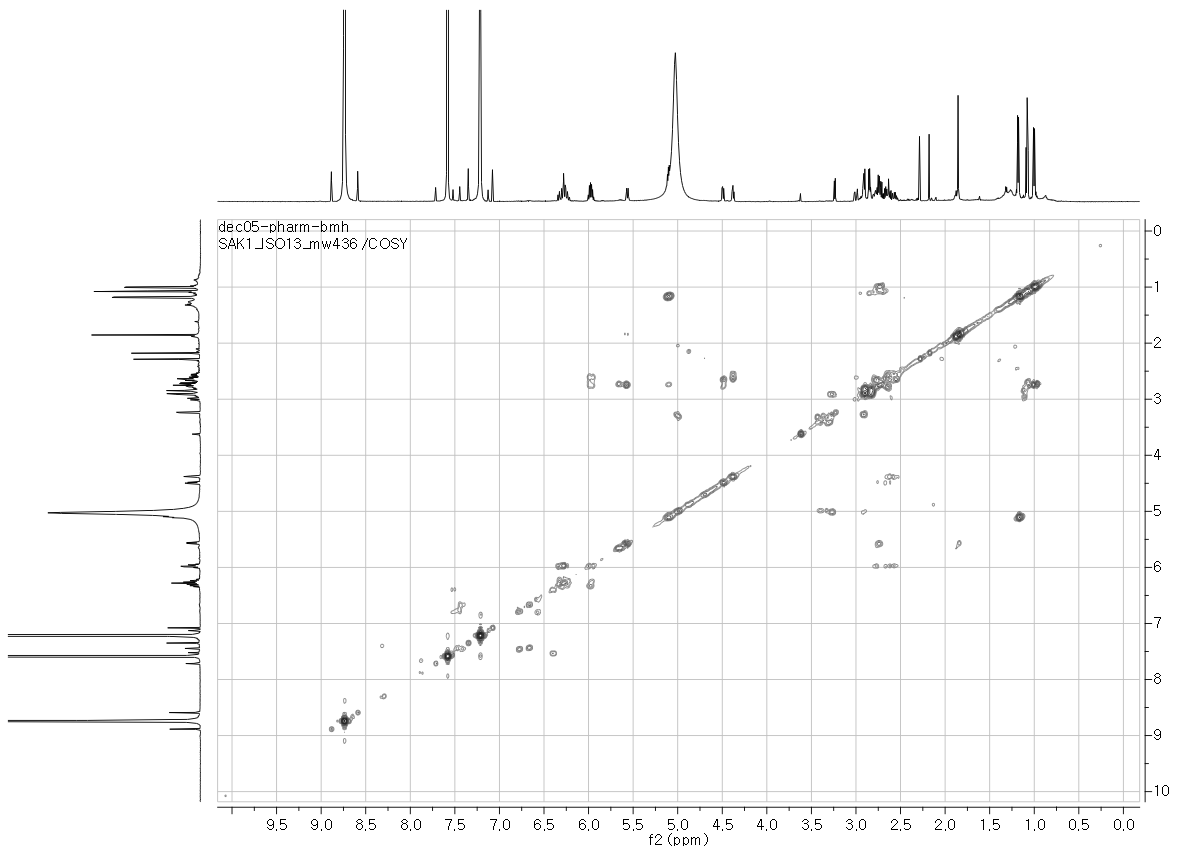


**Figure S14.** COSY spectrum (600 MHz) of succinilene C (**3**) in pyridine-*d*_5_.


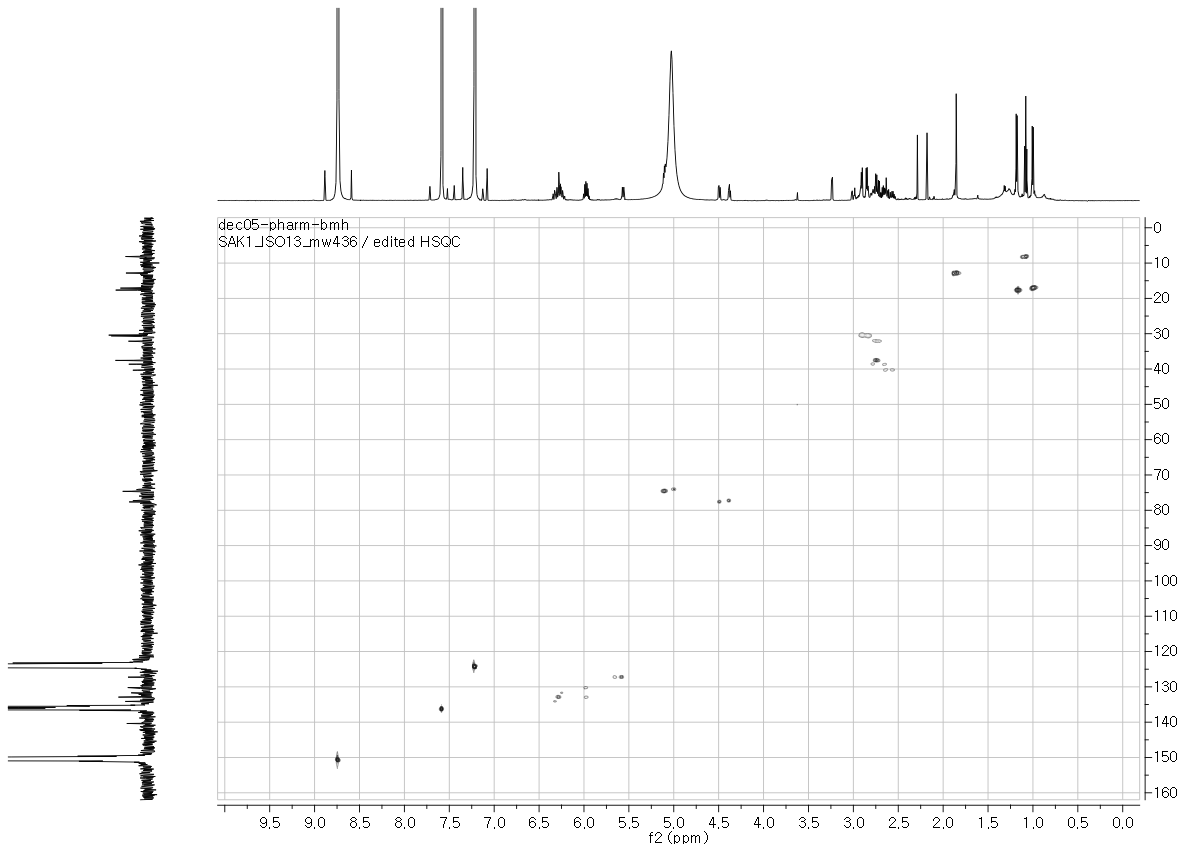


**Figure S15.** HSQC spectrum (600 MHz) of succinilene C (**3**) in pyridine-*d*_5_.


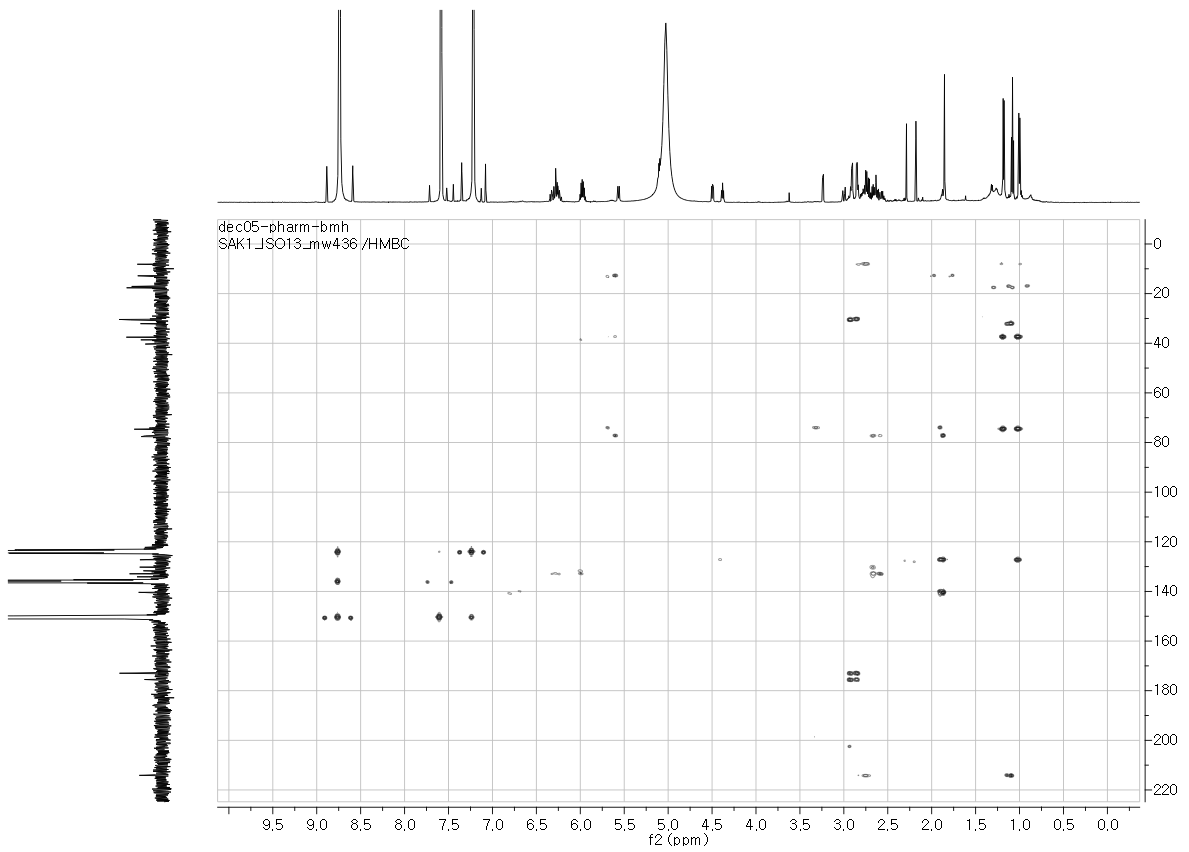


**Figure S16.** HMBC spectrum (600 MHz) of succinilene C (**3**) in pyridine-*d*_5_.


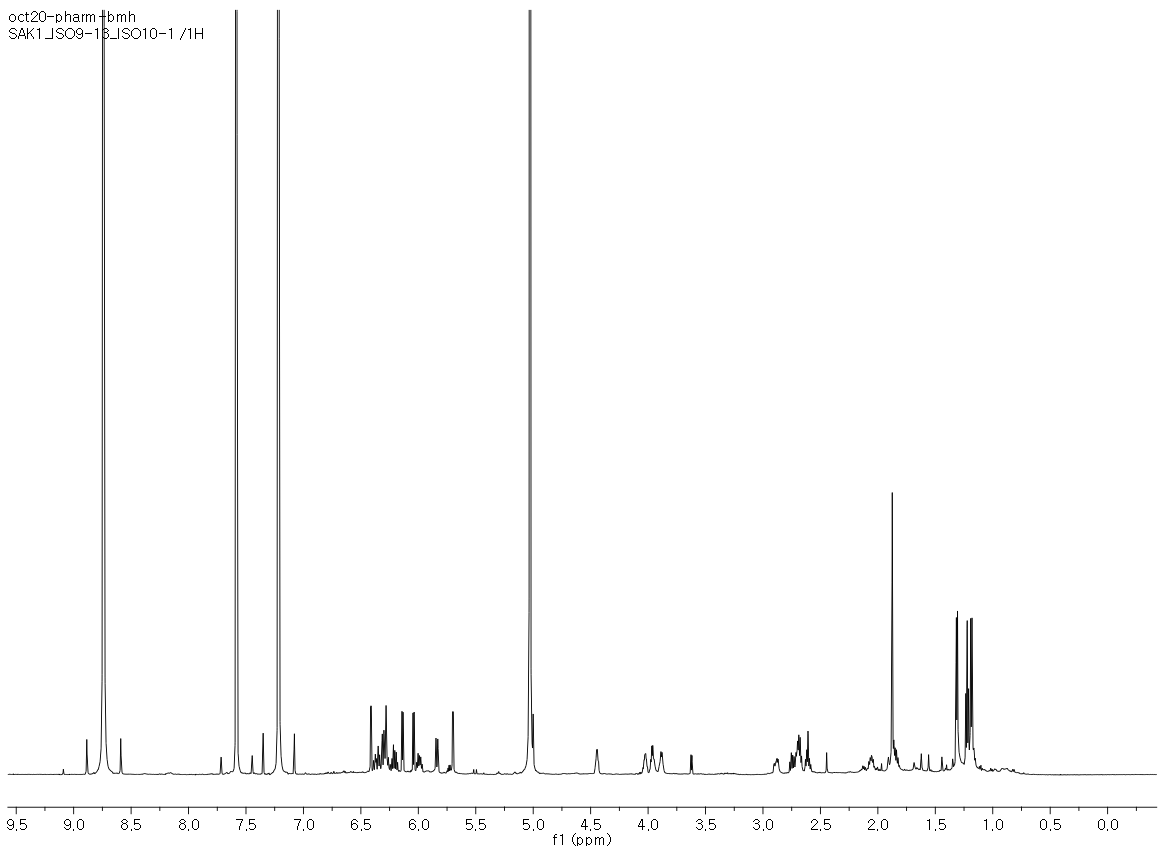


**Figure S17.** ^1^H NMR spectrum (600 MHz) of succinilene D (**4**) in pyridine-*d*_5_.


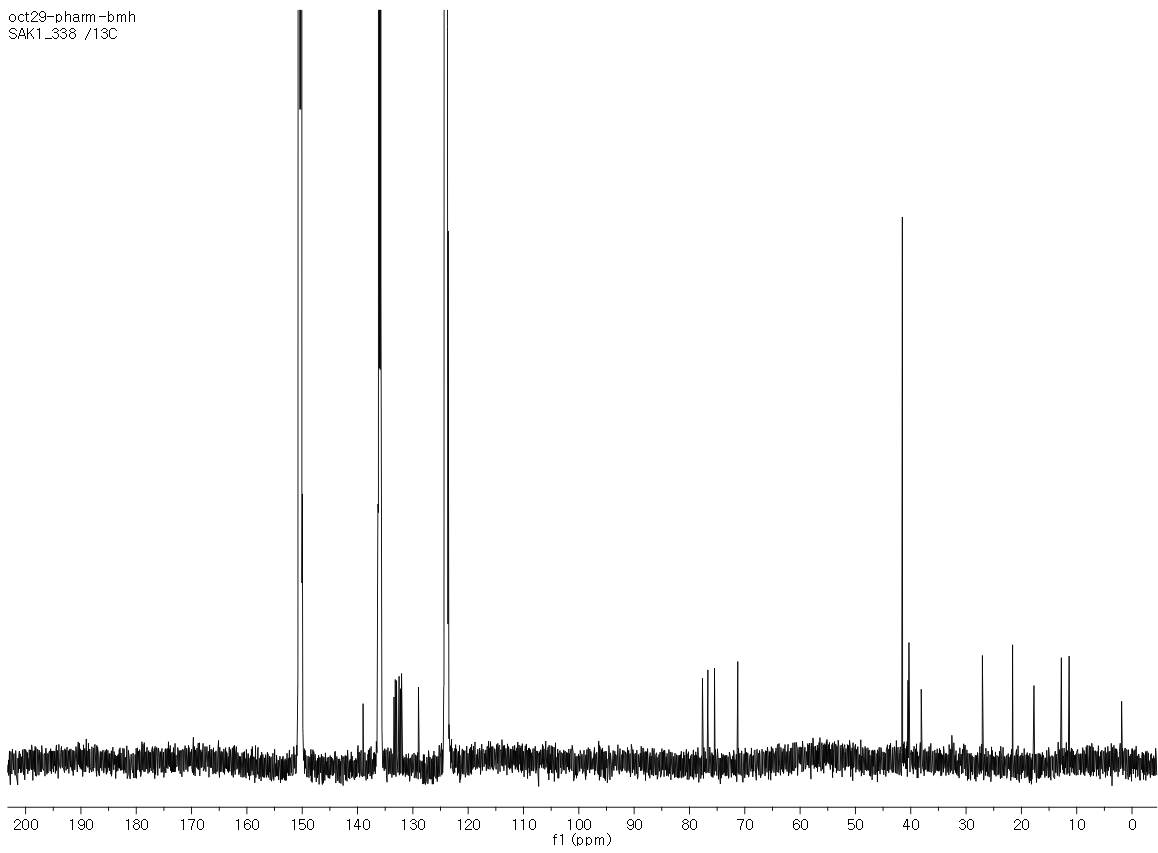


**Figure S18.** ^13^C NMR spectrum (150 MHz) of succinilene D (**4**) in pyridine-*d*_5_.


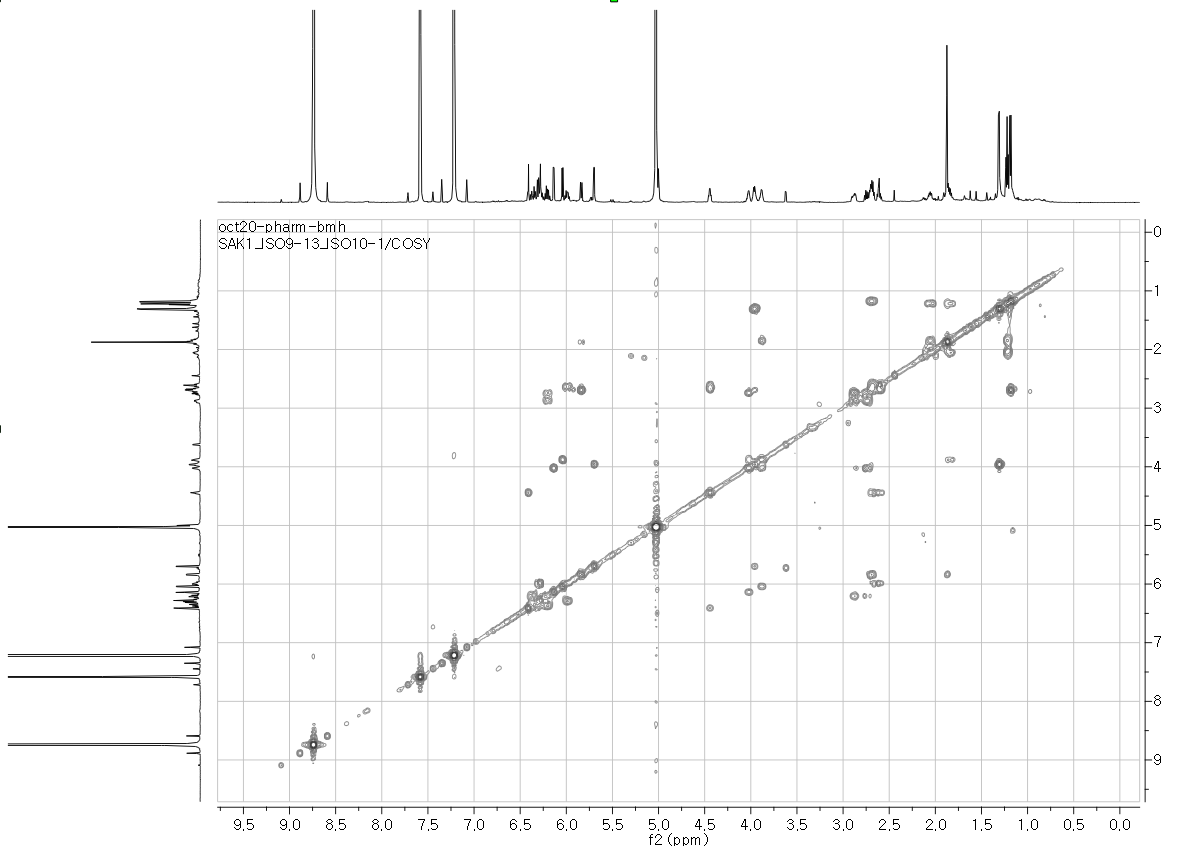


**Figure S19.** COSY spectrum (600 MHz) of succinilene D (**4**) in pyridine-*d*_5_.


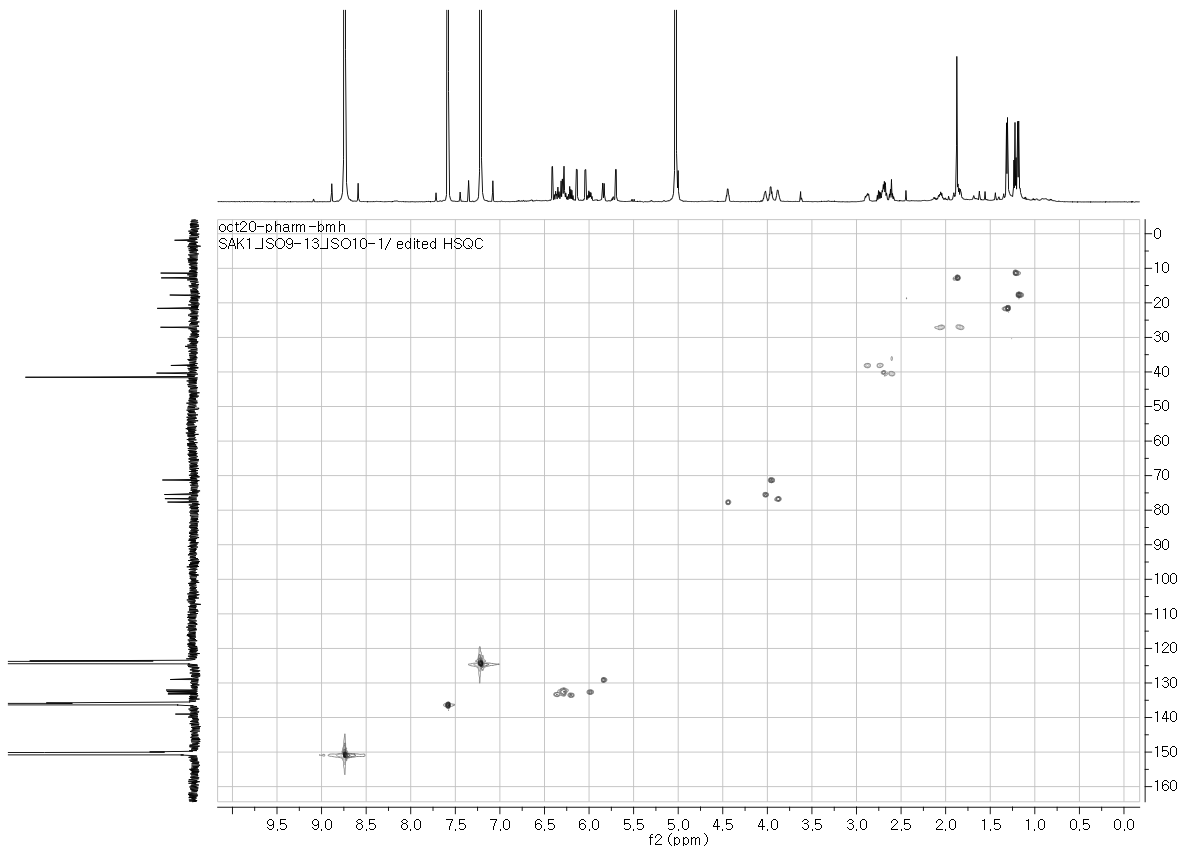


**Figure S20.** HSQC spectrum (600 MHz) of succinilene D (**4**) in pyridine-*d*_5_.


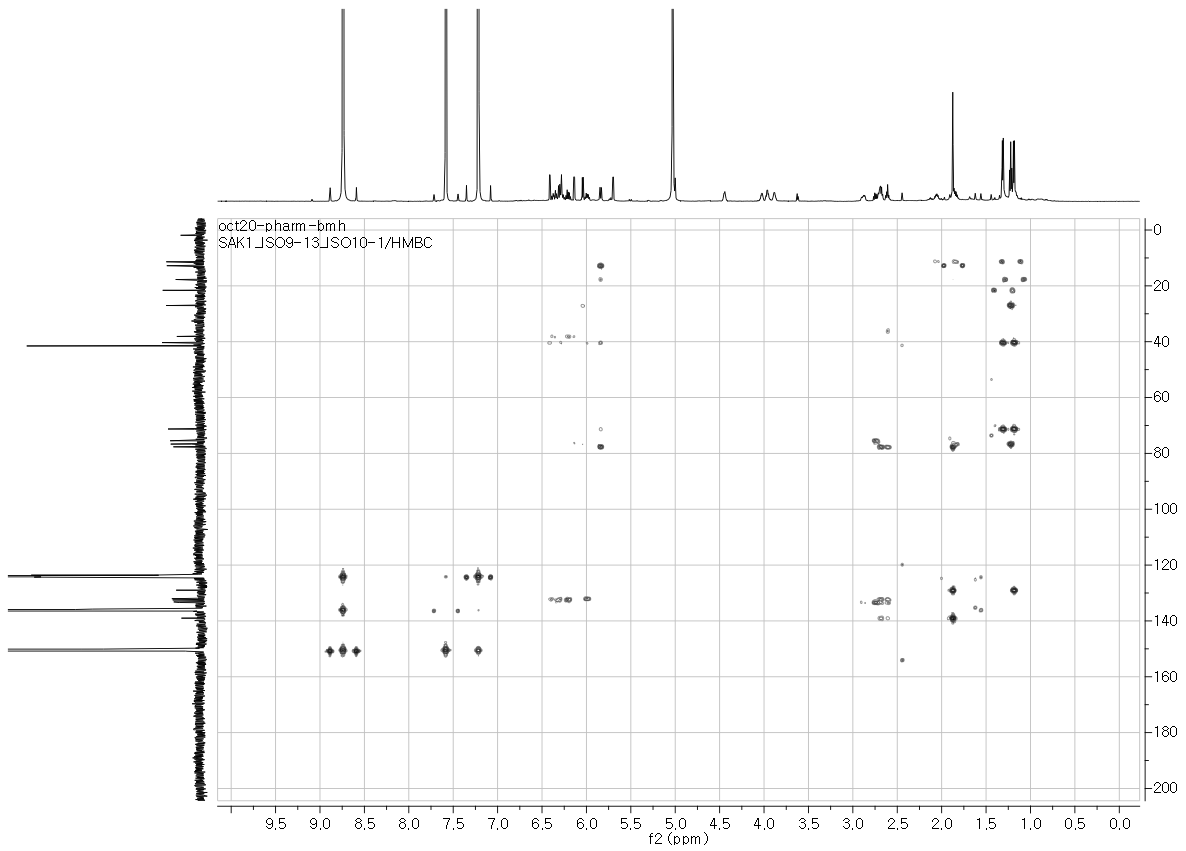


**Figure S21.** HMBC spectrum (600 MHz) of succinilene D (**4**) in pyridine-*d*_5_.


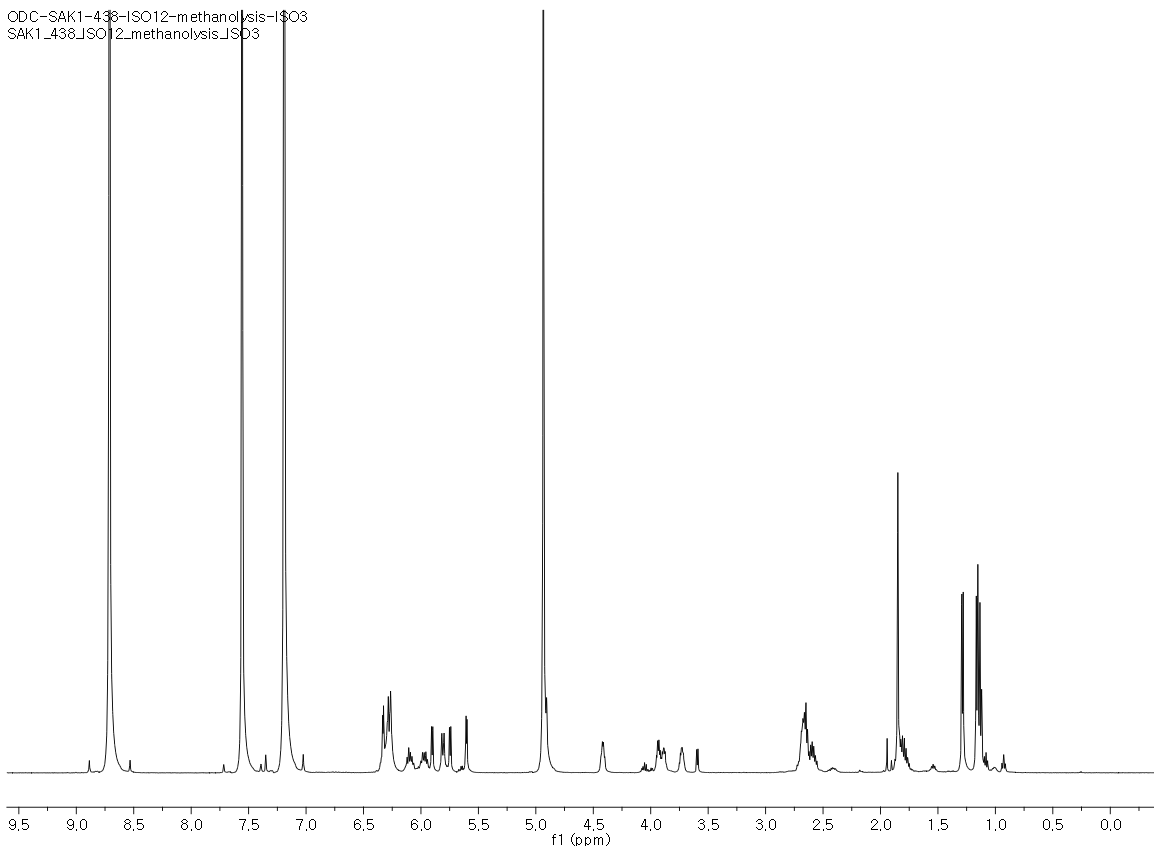


**Figure S22.** ^1^H NMR spectrum (600 MHz) of methanolysis product of succinilene A (**5**) in pyridine-*d*_5_.


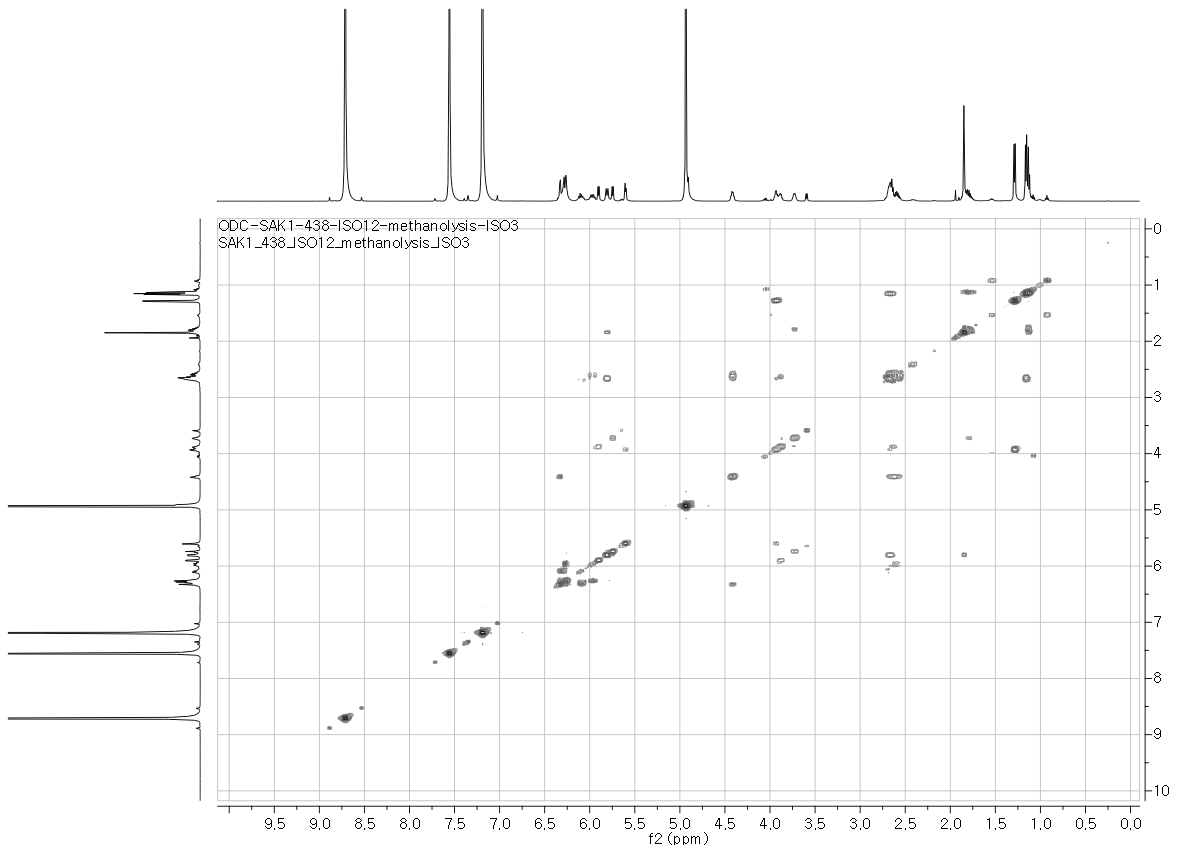


**Figure S23.** COSY NMR spectrum (600 MHz) of methanolysis product of succinilene A (**5**) in pyridine-*d*_5_.


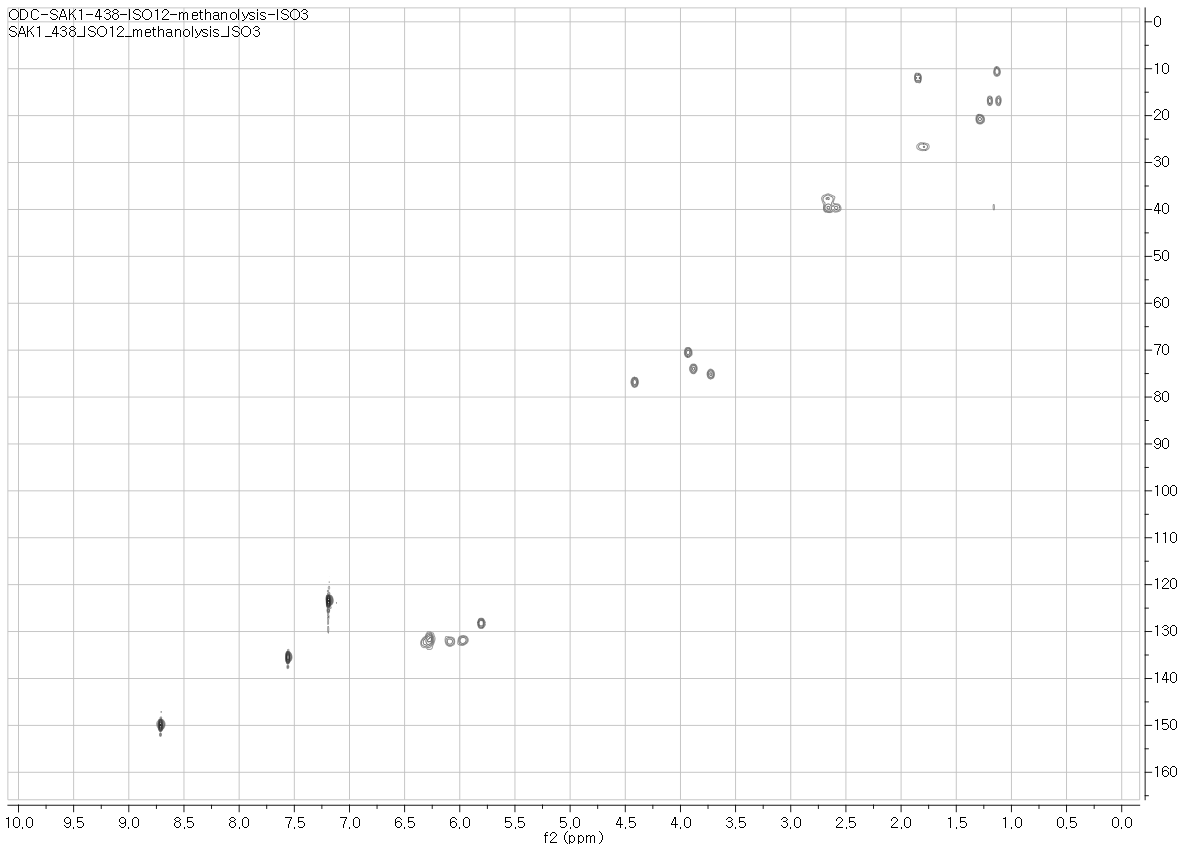


**Figure S24.** HSQC NMR spectrum (600 MHz) of methanolysis product of succinilene A (**5**) in pyridine-*d*_5_.


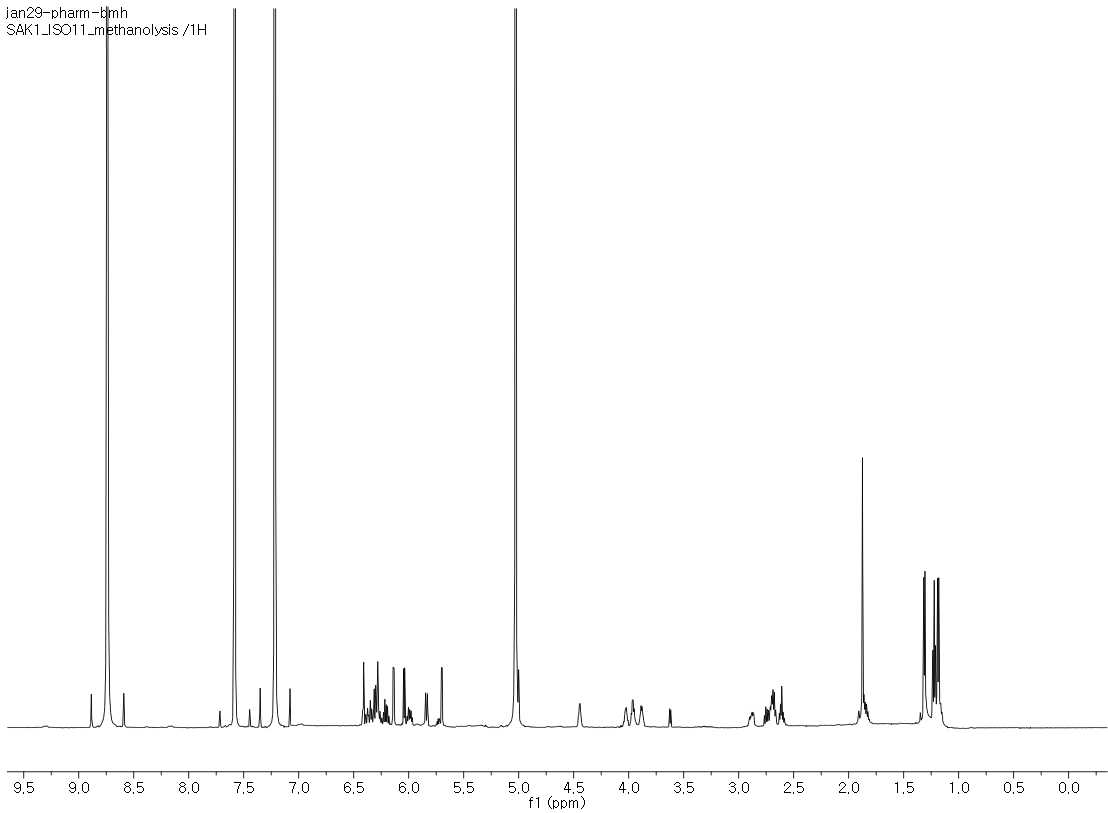


**Figure S25.** ^1^H NMR spectrum (600 MHz) of methanolysis product of succinilene B (**6**) in pyridine-*d*_5_.


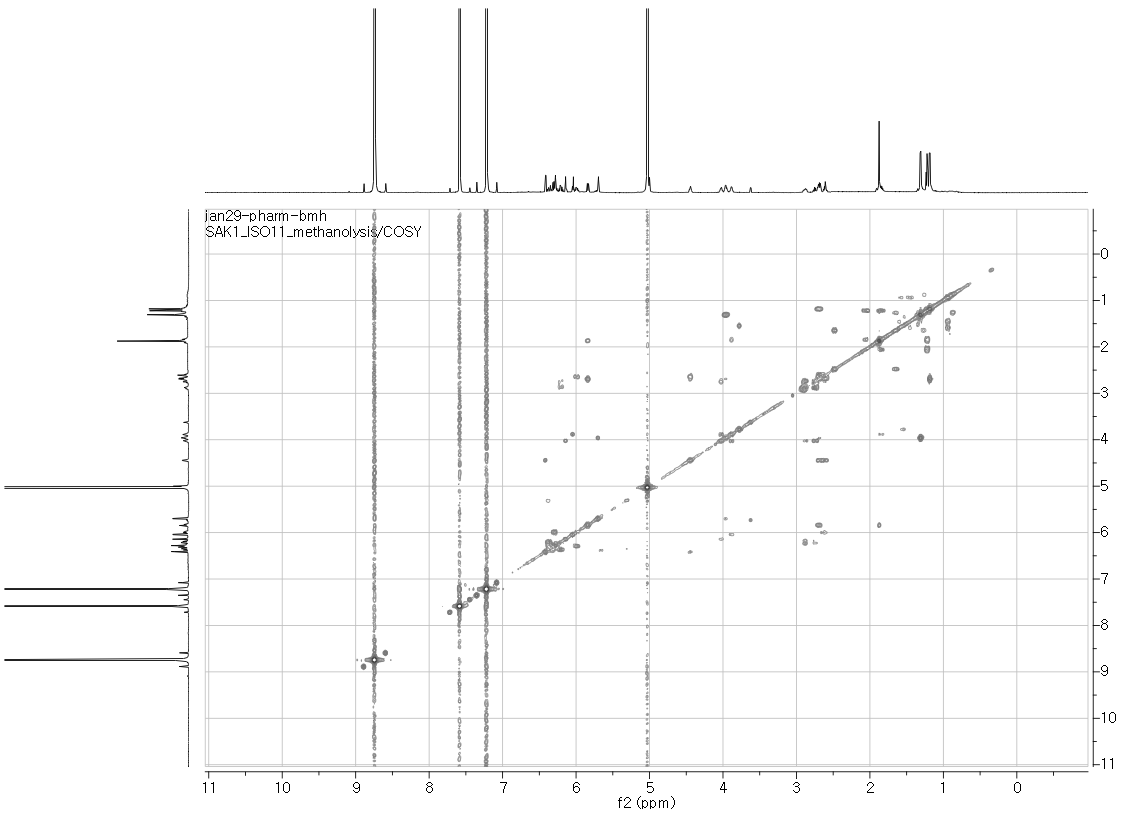


**Figure S26.** COSY NMR spectrum (600 MHz) of methanolysis product of succinilene B (**6**) in pyridine-*d*_5_.


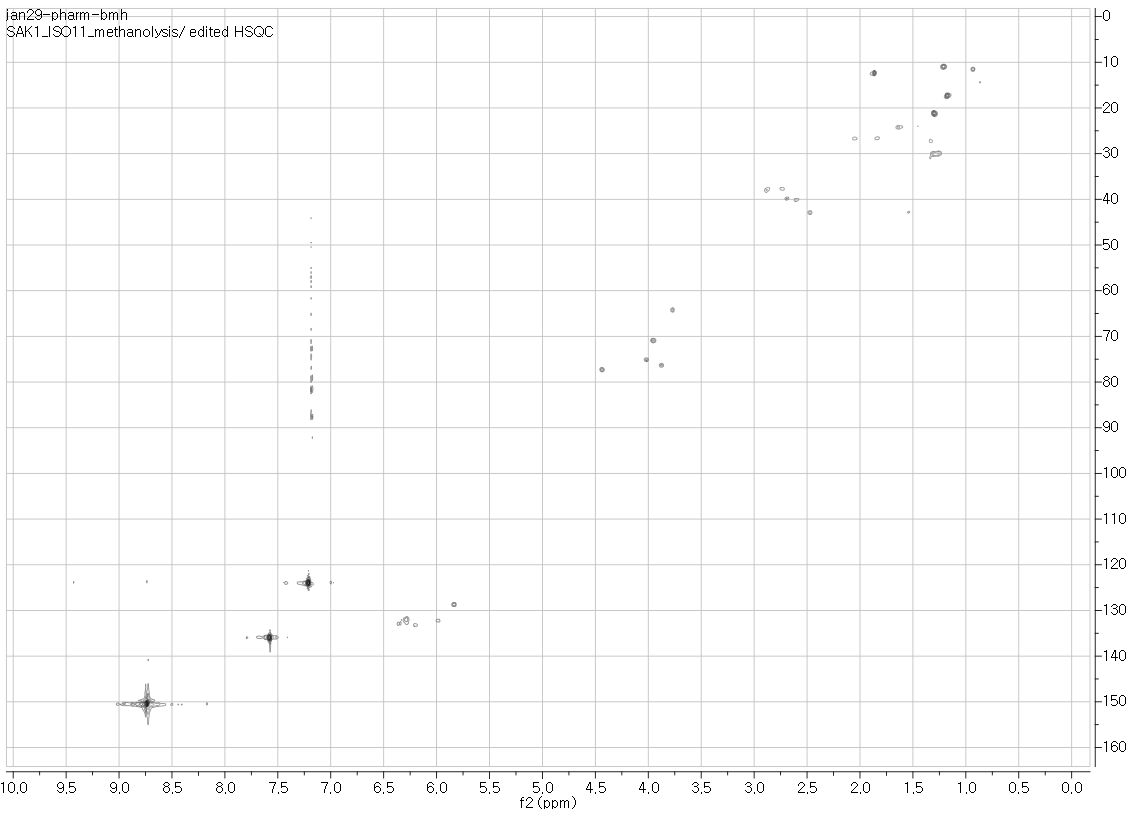


**Figure S27.** HSQC NMR spectrum (600 MHz) of methanolysis product of succinilene B (**6**) in pyridine-*d*_5_.


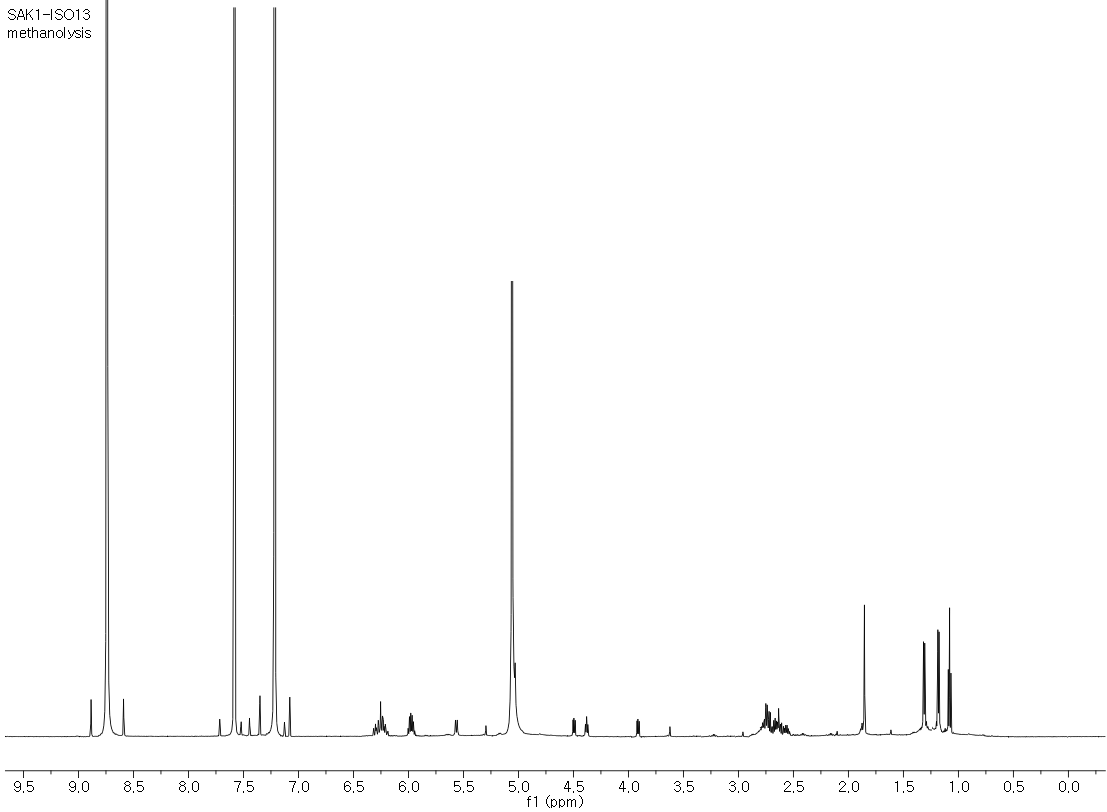


**Figure S28.** ^1^H NMR spectrum (600 MHz) of methanolysis product of succinilene C (**7**) in pyridine-*d*_5_.


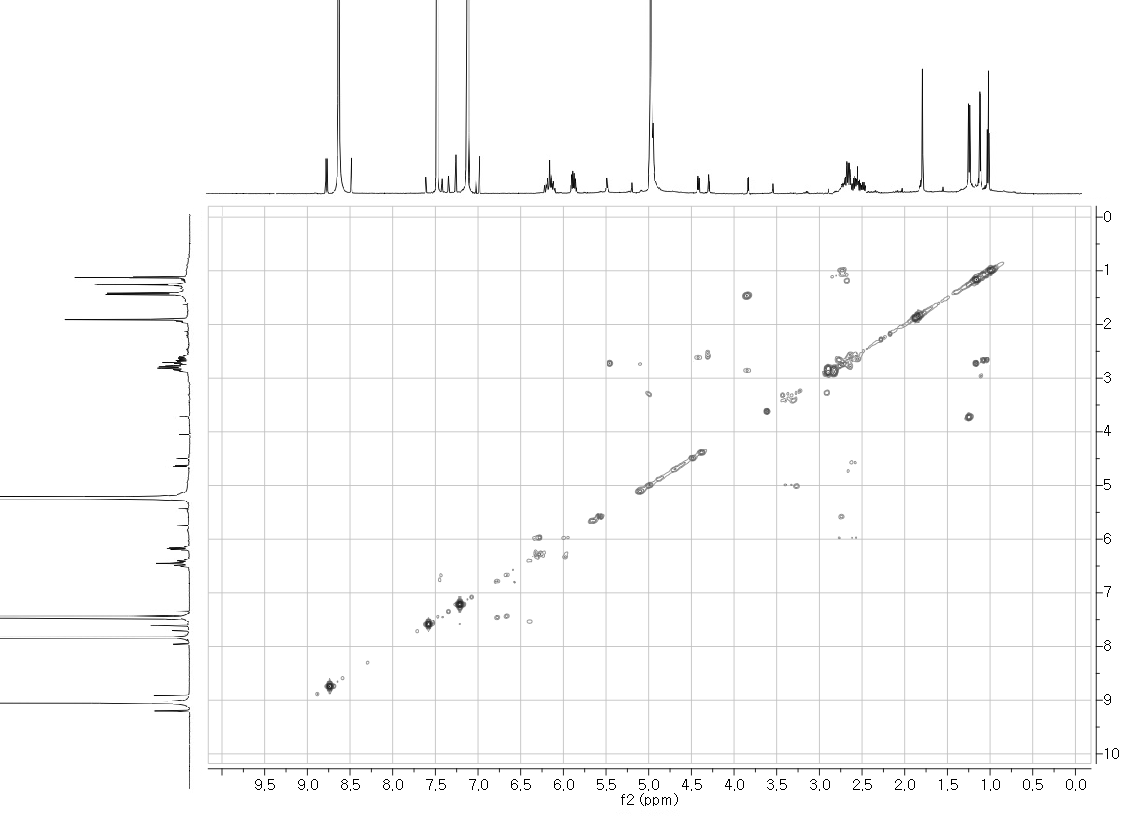


**Figure S29.** COSY NMR spectrum (600 MHz) of methanolysis product of succinilene C (**7**) in pyridine-*d*_5_.


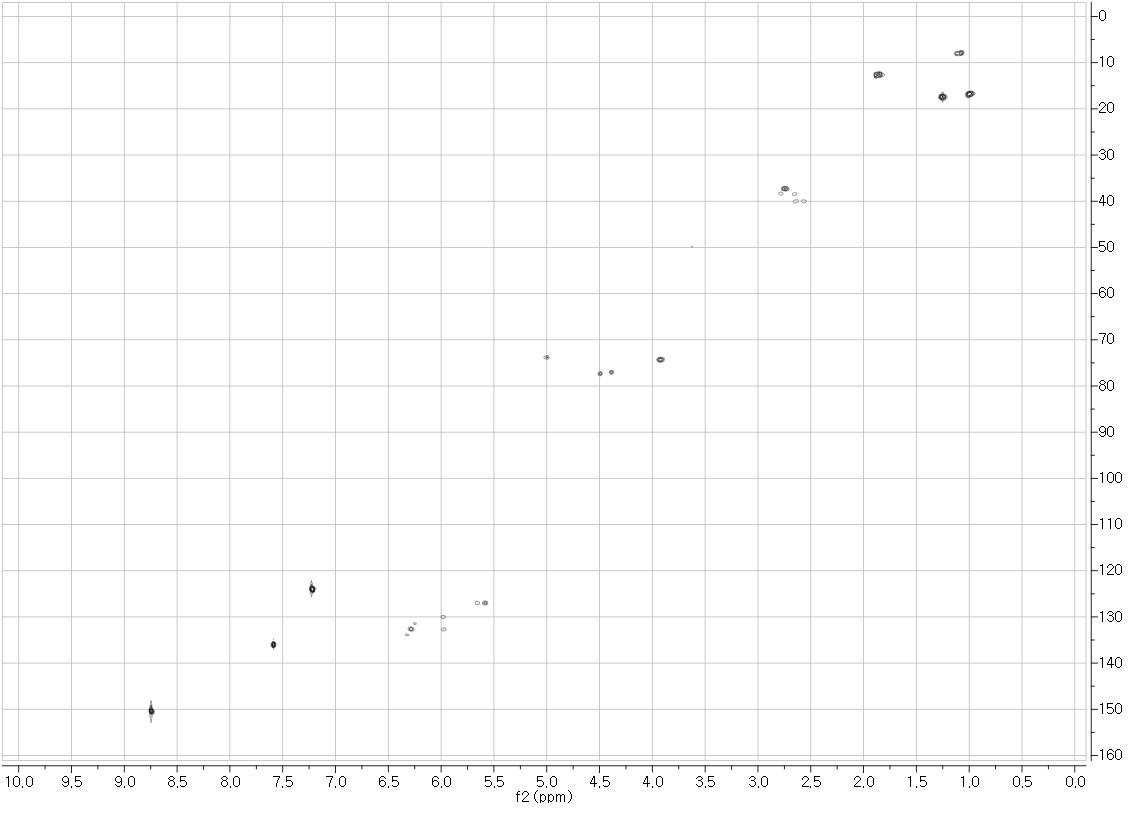


**Figure S30.** HSQC NMR spectrum (600 MHz) of methanolysis product of succinilene C (**7**) in pyridine-*d*_5_.


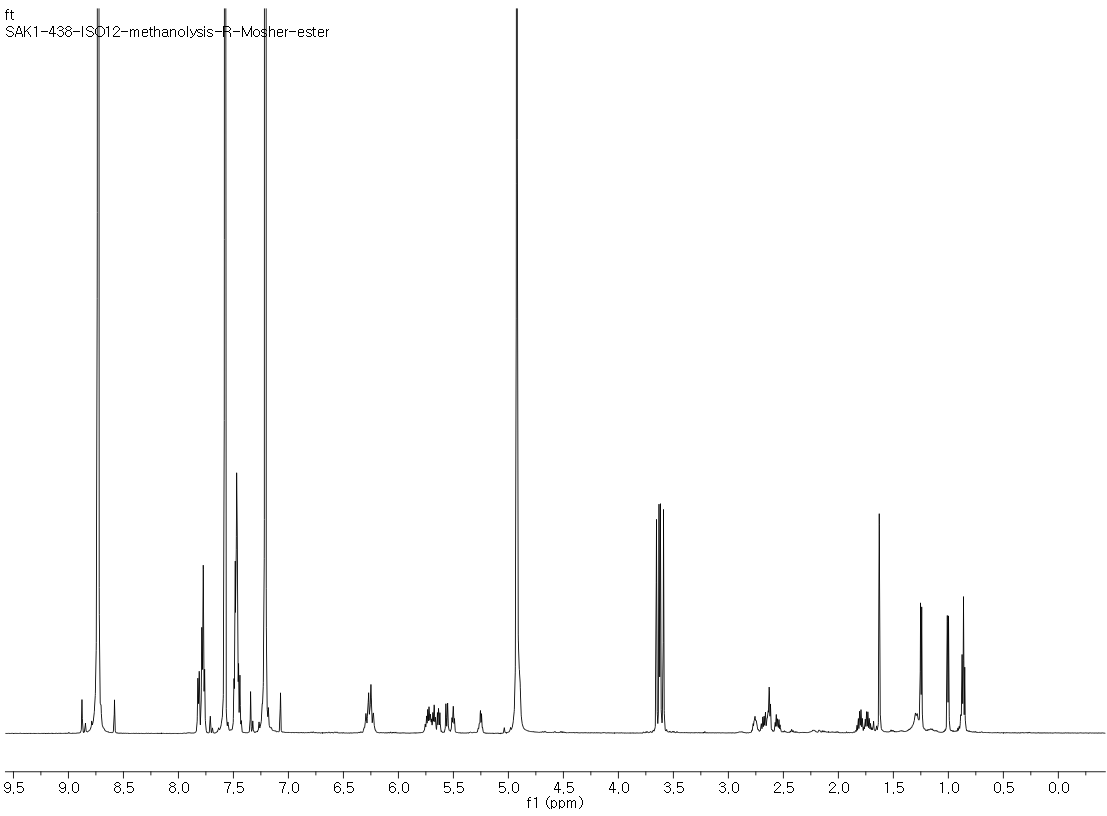


**Figure S31.** ^1^H NMR spectrum (600 MHz) of *S*-MTPA ester (**8**) of **5** in pyridine-*d*_5_.


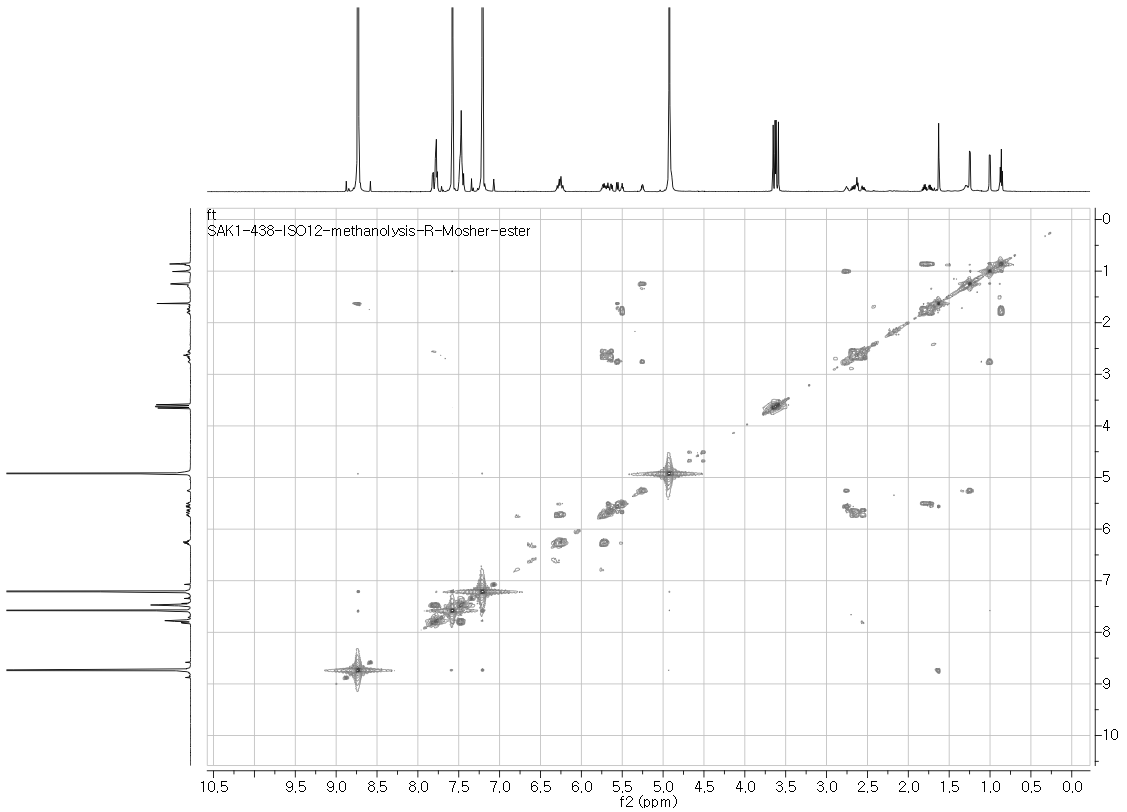


**Figure S32.** COSY NMR spectrum (600 MHz) of *S*-MTPA ester (**8**) of **5** in pyridine-*d*_5_.


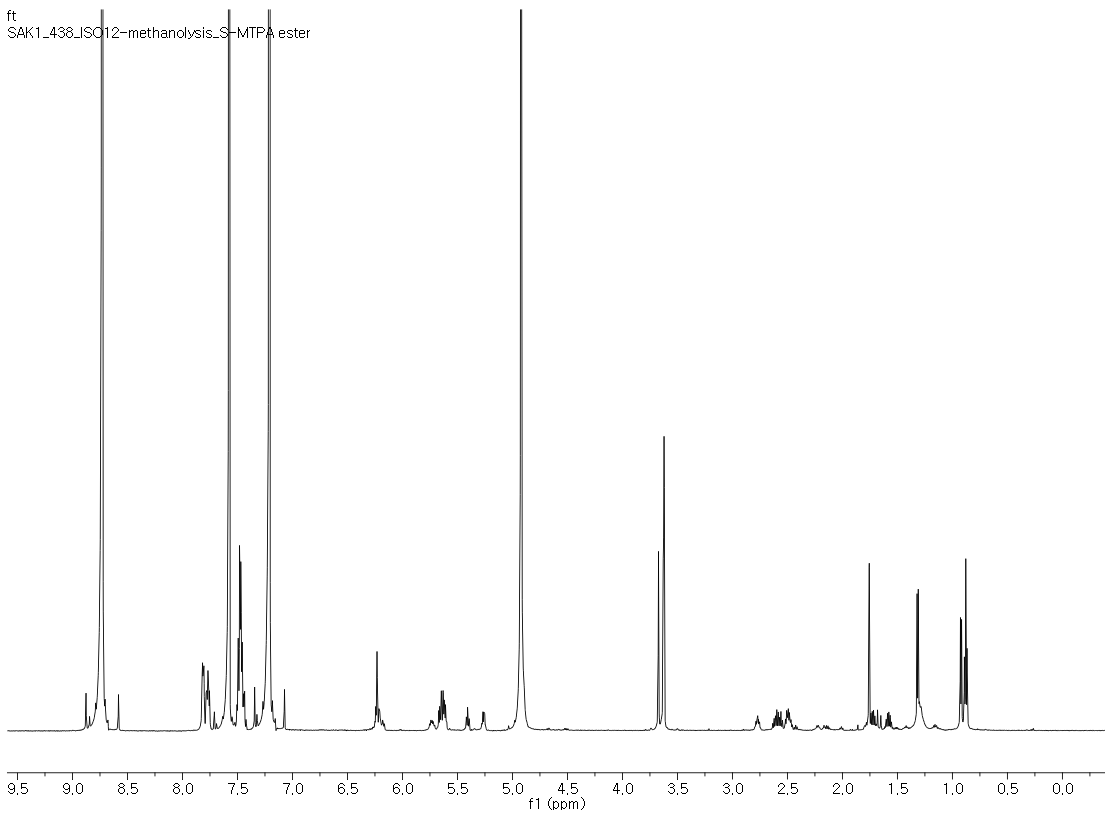


**Figure S33.** ^1^H NMR spectrum (600 MHz) of *R*-MTPA ester (**9**) of **5** in pyridine-*d*_5_.


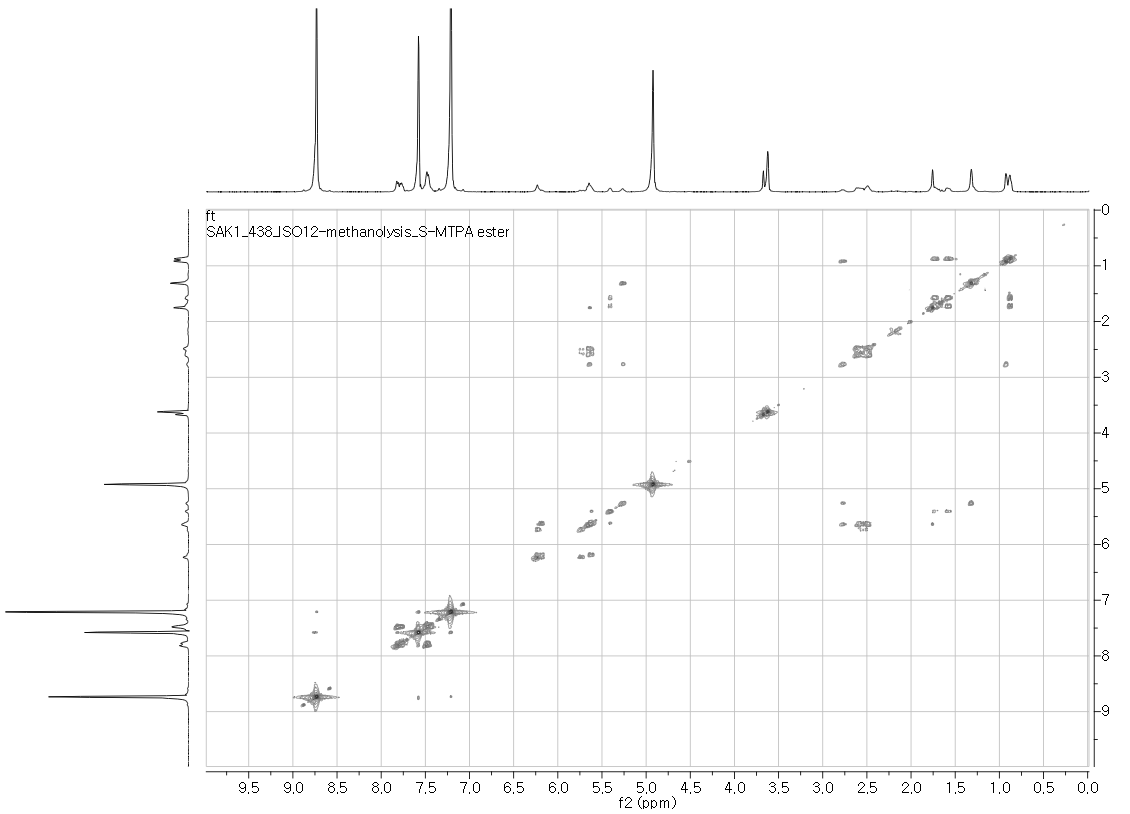


**Figure S34.** COSY NMR spectrum (600 MHz) of *R*-MTPA ester (**9**) of **5** in pyridine-*d*_5_.


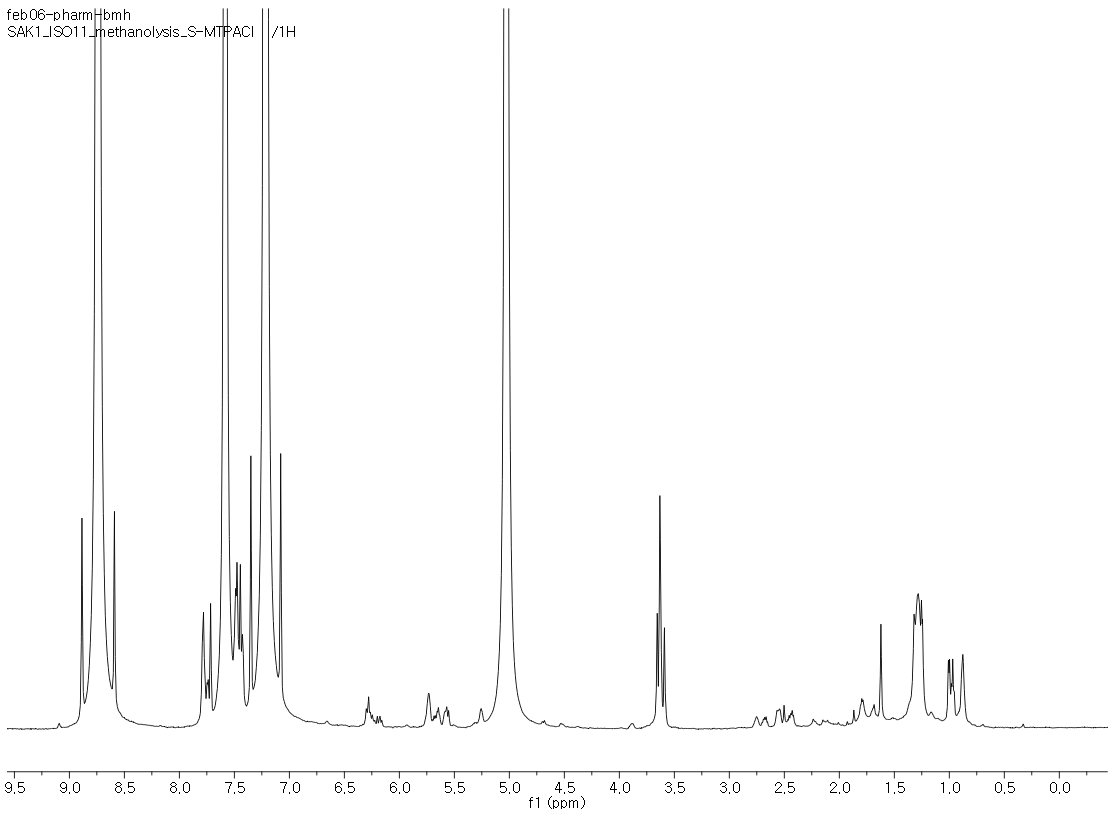


**Figure S35.** ^1^H NMR spectrum (600 MHz) of *S*-MTPA ester (**10**) of **6** in pyridine-*d*_5_.


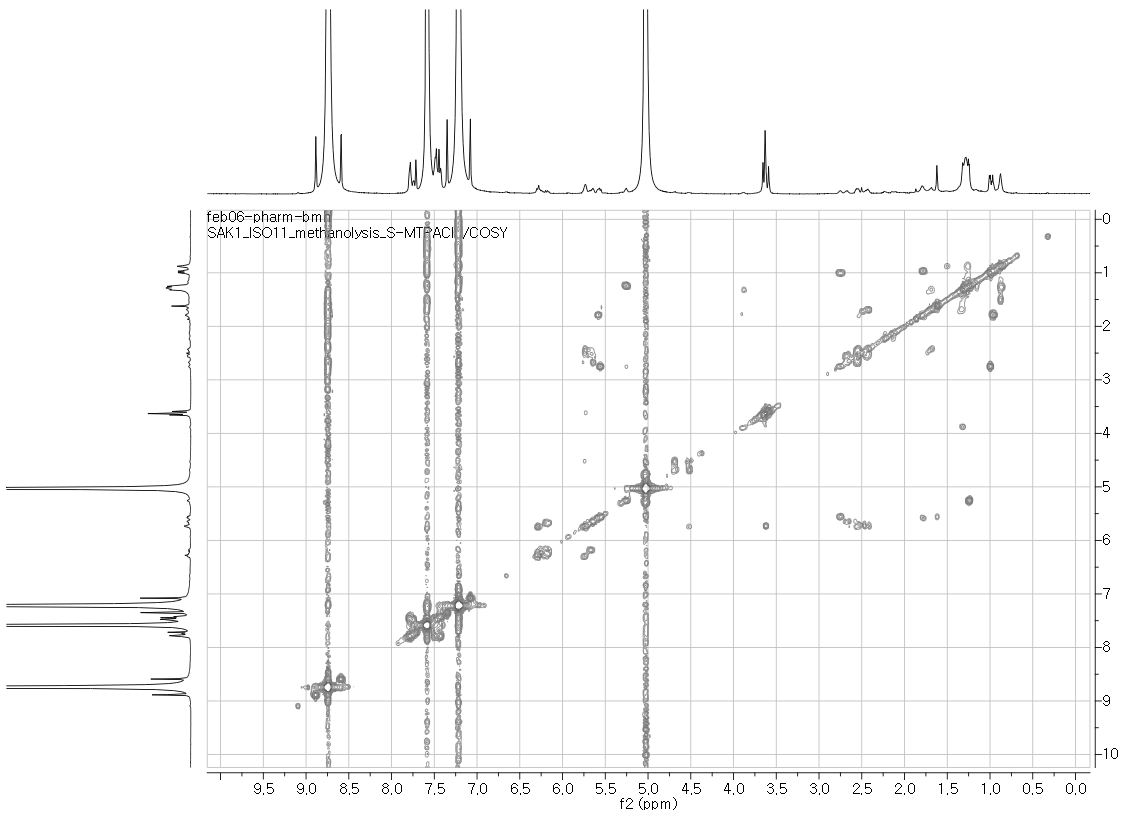


**Figure S36.** COSY NMR spectrum (600 MHz) of *S*-MTPA ester (**10**) of **6** in pyridine-*d*_5_.


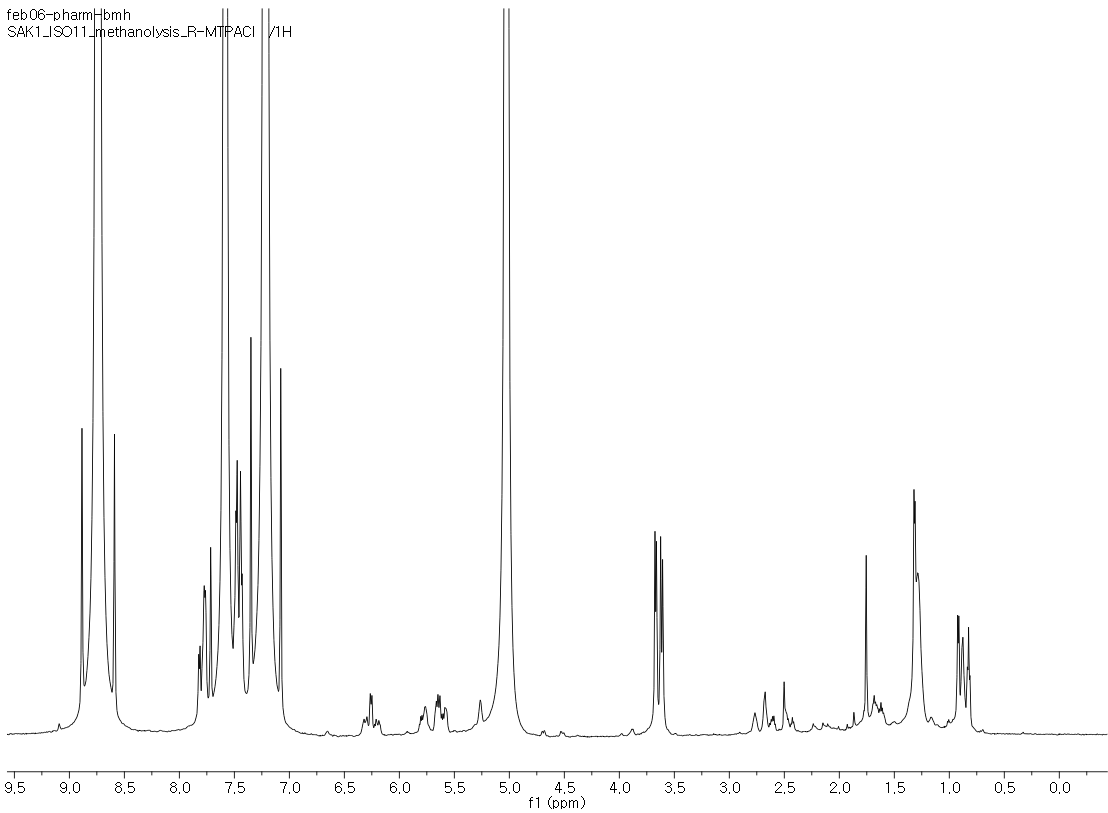


**Figure S37.** ^1^H NMR spectrum (600 MHz) of *R*-MTPA ester (**11**) of **6** in pyridine-*d*_5_.


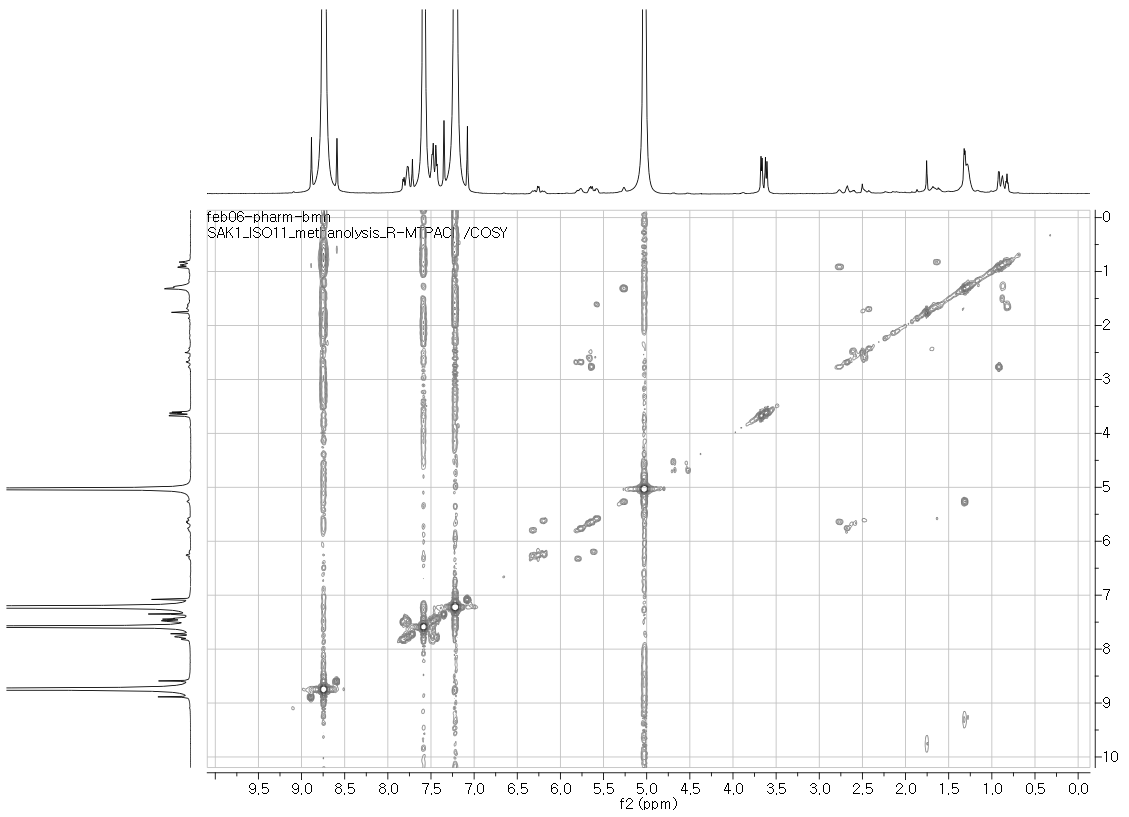


**Figure S38.** COSY NMR spectrum (600 MHz) of *R*-MTPA ester (**11**) of **6** in pyridine-*d*_5_.


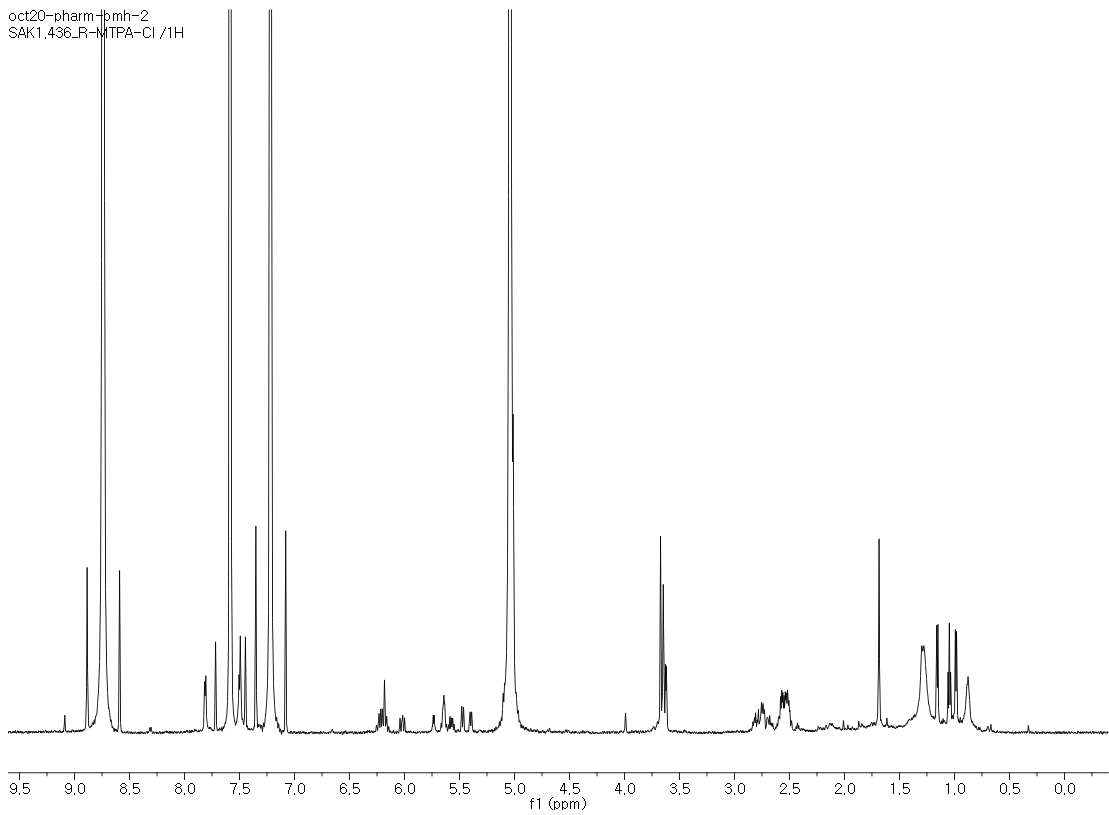


**Figure S39.** ^1^H NMR spectrum (600 MHz) of *S*-MTPA ester (**12**) of **7** in pyridine-*d*_5_.


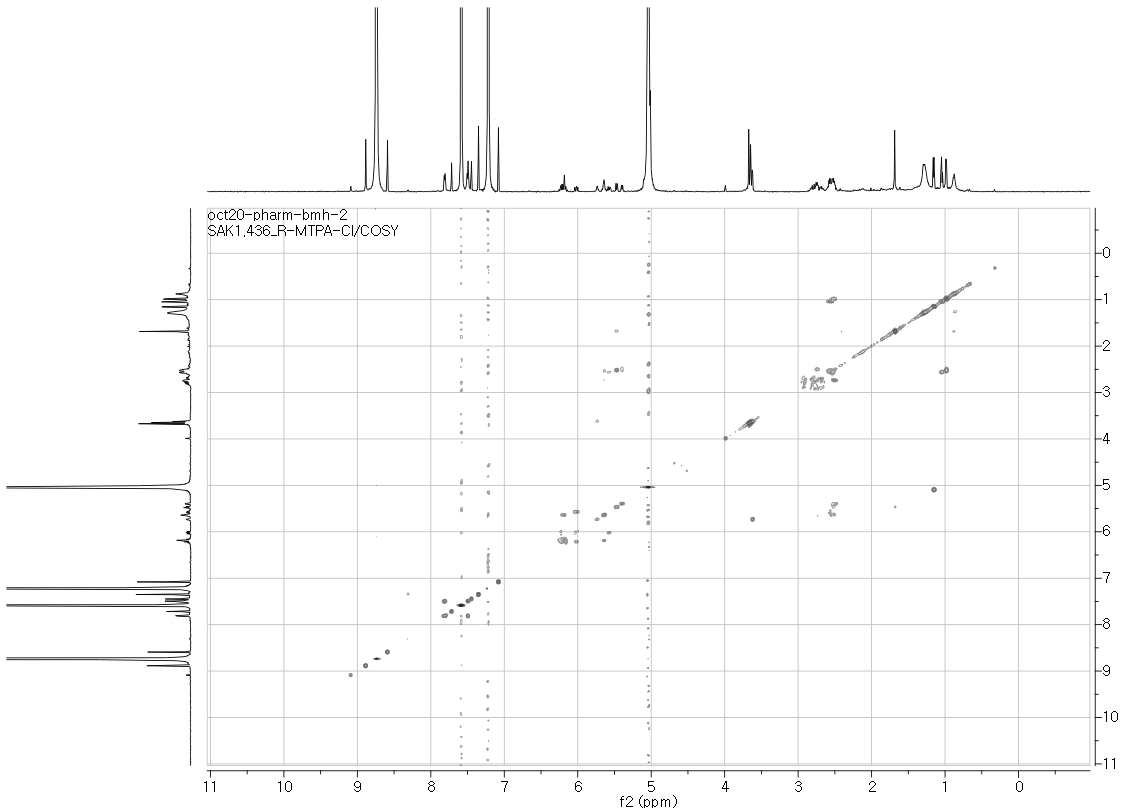


**Figure S40.** COSY NMR spectrum (600 MHz) of *S*-MTPA ester (**12**) of **7** in pyridine-*d*_5_.


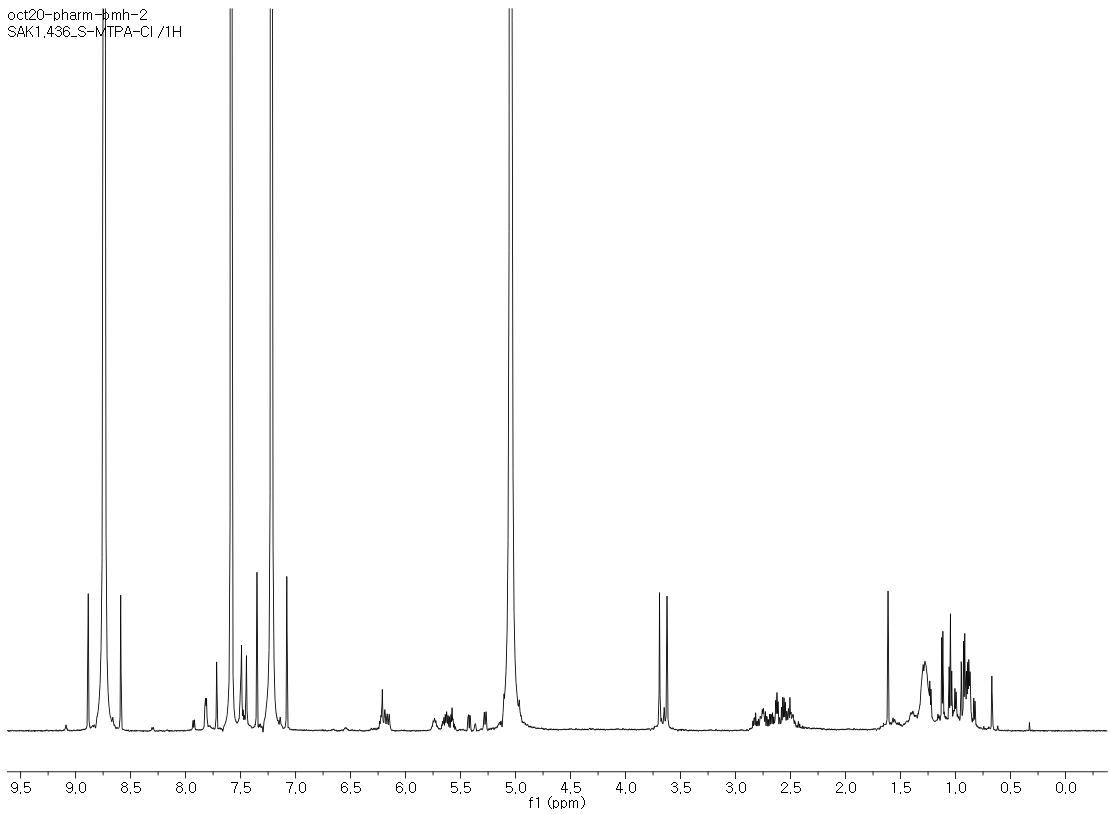


**Figure S41.** ^1^H NMR spectrum (600 MHz) of *R*-MTPA ester (**13**) of **7** in pyridine-*d*_5_.


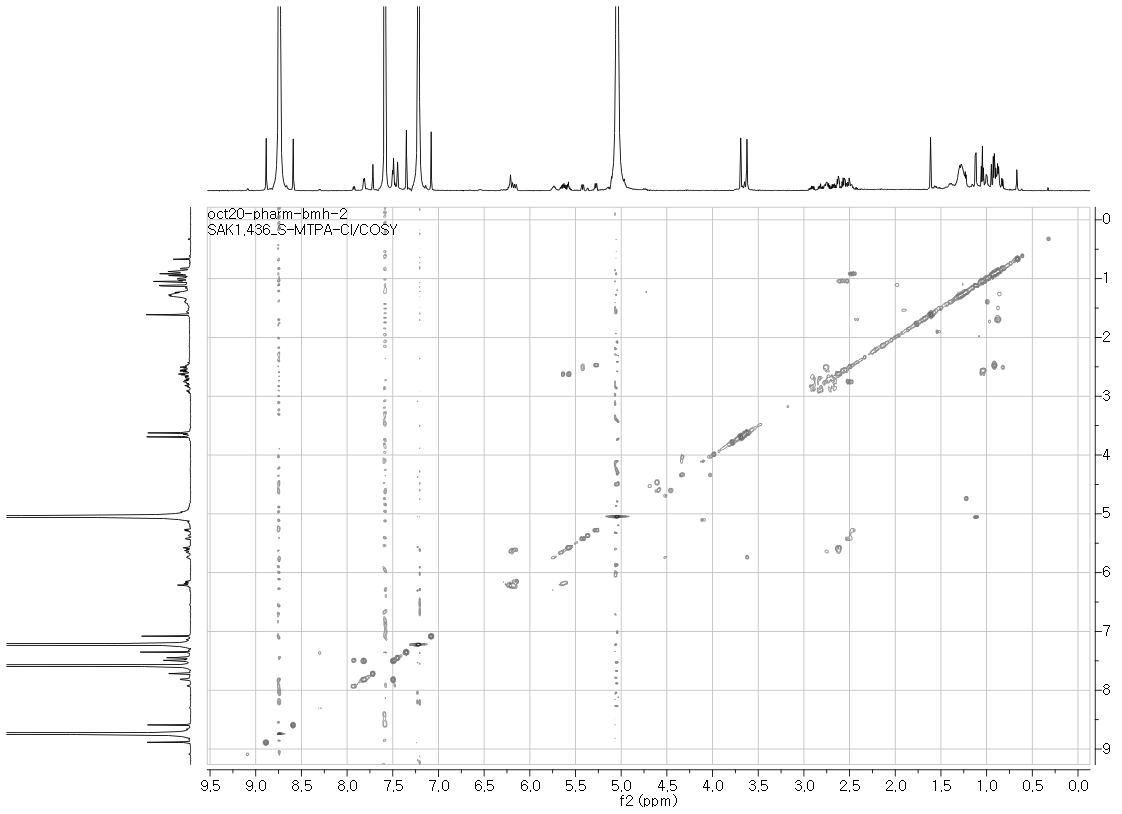


**Figure S42.** COSY NMR spectrum (600 MHz) of *R*-MTPA ester (**13**) of **7** in pyridine-*d*_5_.

**Figure S43.** ECD spectra of **5**, **6**, and succinilene D (**4**).

ACTIVE SPECIES:1H

CHEMICAL SHIFTS(PPM):

PROTON 2*SPIN= 1 SPECIES=1H POPULATION(Y)= 1.00000

H1 / 1 3.917649 1*1*1 STAT=Y PRED= 3.938 RANGE= 0.389 WIDTH(Y)= 1.511 RESP(Y)= 0.8199 HSQC= C1

H2A / 1 2.674519 1*1*1 STAT=Y PRED= 2.446 RANGE= 0.310 WIDTH(Y)= 2.508 RESP(Y)= 0.8800 HSQC= C2

H2B / 1 2.725337 1*1*1 STAT=Y PRED= 2.354 RANGE= 0.380 WIDTH(Y)= 2.896 RESP(Y)= 0.9623 HSQC= C2

H3 / 1 6.127999 1*1*1 STAT=Y PRED= 5.858 RANGE= 0.738 WIDTH(Y)= 1.743 RESP(Y)= 0.8306 HSQC= C3

H4 / 1 6.366418 1*1*1 STAT=Y PRED= 5.899 RANGE= 0.437 WIDTH(Y)= 2.622 RESP(Y)= 0.8715 HSQC= C4

H5 / 1 6.315759 1*1*1 STAT=Y PRED= 6.204 RANGE= 0.771 WIDTH(Y)= 2.171 RESP(Y)= 0.8625 HSQC= C5

H6 / 1 6.292894 1*1*1 STAT=Y PRED= 6.132 RANGE= 0.781 WIDTH(Y)= 0.916 RESP(Y)= 0.8286 HSQC= C6

H7 / 1 6.288472 1*1*1 STAT=Y PRED= 5.853 RANGE= 0.420 WIDTH(Y)= 2.515 RESP(Y)= 0.9004 HSQC= C7

H8 / 1 5.950620 1*1*1 STAT=Y PRED= 5.342 RANGE= 0.841 WIDTH(Y)= 2.981 RESP(Y)= 0.8624 HSQC= C8

H9A / 1 2.651859 1*1*1 STAT=Y PRED= 2.546 RANGE= 0.360 WIDTH(Y)= 2.679 RESP(Y)= 0.9459 HSQC= C9

H9B / 1 2.563074 1*1*1 STAT=Y PRED= 2.419 RANGE= 0.369 WIDTH(Y)= 2.537 RESP(Y)= 0.8639 HSQC= C9

H10 / 1 3.757877 1*1*1 STAT=Y PRED= 3.419 RANGE= 0.350 WIDTH(Y)= 1.487 RESP(Y)= 0.8194 HSQC= C10

H12A/ 1 1.857836 1*1*1 STAT=Y PRED= 1.526 RANGE= 0.310 WIDTH(Y)= 1.186 RESP(Y)= 0.7444 HSQC= C12

H12B/ 1 1.810485 1*1*1 STAT=Y PRED= 1.477 RANGE= 0.310 WIDTH(Y)= 1.671 RESP(Y)= 0.8193 HSQC= C12

H14 / 1 1.161893 1*1*3 STAT=Y PRED= 0.938 RANGE= 0.168 WIDTH(Y)= 1.579 RESP(Y)= 0.9405 HSQC= C14

H15 / 1 4.374450 1*1*1 STAT=Y PRED= 4.170 RANGE= 0.390 WIDTH(Y)= 2.618 RESP(Y)= 0.9421 HSQC= C15

H18 / 1 5.542620 1*1*1 STAT=Y PRED= 5.128 RANGE= 0.811 WIDTH(Y)= 3.922 RESP(Y)= 0.9392 HSQC= C18

H19 / 1 1.861640 1*1*3 STAT=Y PRED= 1.735 RANGE= 0.260 WIDTH(Y)= 2.745 RESP(Y)= 0.9556 HSQC= C19

H20 / 1 2.739650 1*1*1 STAT=Y PRED= 2.452 RANGE= 0.427 WIDTH(Y)= 2.042 RESP(Y)= 0.9075 HSQC= C20

H21 / 1 5.089846 1*1*1 STAT=Y PRED= 4.990 RANGE= 0.409 WIDTH(Y)= 2.610 RESP(Y)= 1.0000 HSQC= C21

H24A/ 1 2.851613 1*1*1 STAT=Y PRED= 2.606 RANGE= 0.310 WIDTH(Y)= 3.132 RESP(Y)= 0.9294 HSQC= C24

H24B/ 1 2.843561 1*1*1 STAT=Y PRED= 3.032 RANGE= 0.370 WIDTH(Y)= 2.921 RESP(Y)= 0.9378 HSQC= C24

H25A/ 1 2.904633 1*1*1 STAT=Y PRED= 2.591 RANGE= 0.390 WIDTH(Y)= 3.127 RESP(Y)= 0.9508 HSQC= C25

H25B/ 1 2.895392 1*1*1 STAT=Y PRED= 2.574 RANGE= 0.318 WIDTH(Y)= 2.899 RESP(Y)= 0.8987 HSQC= C25

H28 / 1 1.190717 1*1*3 STAT=Y PRED= 1.117 RANGE= 0.270 WIDTH(Y)= 1.975 RESP(Y)= 0.9109 HSQC= C28

H29 / 1 0.997341 1*1*3 STAT=Y PRED= 0.979 RANGE= 0.310 WIDTH(Y)= 2.006 RESP(Y)= 0.8517 HSQC= C29

COUPLING CONSTANTS(HZ):

J32_33 8.0240 J H1 H2A STAT=Y PRED= 5.581 RANGE= 3.680

J32_34 4.6632 J H1 H2B STAT=Y PRED= 5.007 RANGE= 3.780

J32_43 4.3273 J H1 H10 STAT=Y PRED= 4.599 RANGE= 3.430

J33_34 -14.3641 J H2A H2B STAT=Y PRED= -13.852 RANGE= 1.520

J33_35 7.1199 J H2A H3 STAT=Y PRED= 6.994 RANGE= 2.000

J33_36 -0.0583 J H2A H4 STAT=Y PRED= -1.429 RANGE= 1.000

J34_35 7.4988 J H2B H3 STAT=Y PRED= 7.222 RANGE= 2.000

J34_36 -0.2114 J H2B H4 STAT=Y PRED= -1.360 RANGE= 1.000

J35_36 15.1946 J H3 H4 STAT=Y PRED= 16.224 RANGE= 1.260

J36_37 10.4909 J H4 H5 STAT=Y PRED= 10.529 RANGE= 1.920

J37_38 14.0289 J H5 H6 STAT=Y PRED= 15.374 RANGE= 1.010

J38_39 10.7744 J H6 H7 STAT=Y PRED= 10.529 RANGE= 1.920

J39_40 14.6476 J H7 H8 STAT=Y PRED= 16.234 RANGE= 1.270

J39_41 -0.0041 J H7 H9A STAT=Y PRED= -1.397 RANGE= 1.000

J39_42 -0.0465 J H7 H9B STAT=Y PRED= -1.359 RANGE= 1.000

J40_41 7.3003 J H8 H9A STAT=Y PRED= 7.038 RANGE= 2.000

J40_42 7.5023 J H8 H9B STAT=Y PRED= 7.232 RANGE= 2.000

J41_42 -13.9982 J H9A H9B STAT=Y PRED= -13.883 RANGE= 1.520

J41_51 7.0395 J H9A H15 STAT=Y PRED= 7.150 RANGE= 2.000

J42_51 6.0359 J H9B H15 STAT=Y PRED= 5.820 RANGE= 2.000

J43_45 3.8789 J H10 H12A STAT=Y PRED= 7.027 RANGE= 2.000

J43_46 8.6772 J H10 H12B STAT=Y PRED= 5.630 RANGE= 2.000

J45_46 -13.8703 J H12A H12B STAT=Y PRED= -14.112 RANGE= 1.000

J45_48 7.3787 J H12A H14 STAT=Y PRED= 7.464 RANGE= 1.000

J46_48 7.4866 J H12B H14 STAT=Y PRED= 7.464 RANGE= 1.000

J53_57 9.7356 J H18 H20 STAT=Y PRED= 7.657 RANGE= 2.000

J57_58 5.4998 J H20 H21 STAT=Y PRED= 5.701 RANGE= 3.310

J57_67 6.8180 J H20 H29 STAT=Y PRED= 6.885 RANGE= 1.000

J58_64 6.3545 J H21 H28 STAT=Y PRED= 6.253 RANGE= 1.000

J59_60 -11.4842 J H24A H24B STAT=Y PRED= -16.347 RANGE= 1.260

J59_61 7.2925 J H24A H25A STAT=Y PRED= 6.166 RANGE= 2.000

J59_62 6.0436 J H24A H25B STAT=Y PRED= 7.536 RANGE= 2.000

J60_61 5.8164 J H24B H25A STAT=Y PRED= 6.248 RANGE= 2.000

J60_62 7.5376 J H24B H25B STAT=Y PRED= 6.166 RANGE= 2.000

J61_62 -16.3866 J H25A H25B STAT=Y PRED= -16.340 RANGE= 1.270

CONTROL PARAMETERS:

Solvent = none (def. 99% enriched)

1.000 = Concentration (vol%, def=1.0%)

0.00100000 = Minimum line-intensity

0.00100000 = Diagonalization criterium (not in use)

900.23440000 = FIELD(1H,MHz), used to transform shifts to ppms

10.44039683 = Left frequency (ppm)

-0.53593486 = Right frequency (ppm)

0.000 = Acquisition time (s, for QMTLS)

0.929 = Line-width (for modes D, P & T, 0=use defaults)

0.150778548 = Data-point resolution (Hz)

44.617 = GAUSSIAN (%, 0=use default from INF)

-1.396 = Dispersion contribution (%, 0=use default from INF)

0.00000000 = Decoupling frequency (for DORES)

END of FILE

**Figure S44.** The ^1^H NMR fingerprint of **1** (PERCH .pms file format).

ACTIVE SPECIES:1H

CHEMICAL SHIFTS(PPM):

PROTON 2*SPIN= 1 SPECIES=1H POPULATION(Y)= 1.00000

H1 / 1 4.032398 1*1*1 STAT=Y PRED= 3.936 RANGE= 0.385 WIDTH(Y)= 4.623 RESP(Y)= 0.8075 HSQC= C1

H2A / 1 2.748141 1*1*1 STAT=Y PRED= 2.349 RANGE= 0.375 WIDTH(Y)= 4.512 RESP(Y)= 0.8313 HSQC= C2

H2B / 1 2.882943 1*1*1 STAT=Y PRED= 2.521 RANGE= 0.380 WIDTH(Y)= 5.015 RESP(Y)= 0.9482 HSQC= C2

H3 / 1 6.214475 1*1*1 STAT=Y PRED= 5.883 RANGE= 0.770 WIDTH(Y)= 3.758 RESP(Y)= 0.8350 HSQC= C3

H4 / 1 6.391306 1*1*1 STAT=Y PRED= 5.931 RANGE= 0.477 WIDTH(Y)= 3.958 RESP(Y)= 0.7977 HSQC= C4

H5 / 1 6.317896 1*1*1 STAT=Y PRED= 6.203 RANGE= 0.800 WIDTH(Y)= 3.637 RESP(Y)= 0.8261 HSQC= C5

H6 / 1 6.277262 1*1*1 STAT=Y PRED= 6.118 RANGE= 0.785 WIDTH(Y)= 3.582 RESP(Y)= 0.8471 HSQC= C6

H7 / 1 6.281371 1*1*1 STAT=Y PRED= 5.977 RANGE= 0.530 WIDTH(Y)= 3.958 RESP(Y)= 0.8209 HSQC= C7

H8 / 1 5.939480 1*1*1 STAT=Y PRED= 5.441 RANGE= 0.755 WIDTH(Y)= 4.801 RESP(Y)= 0.8814 HSQC= C8

H9A / 1 2.558667 1*1*1 STAT=Y PRED= 2.516 RANGE= 0.367 WIDTH(Y)= 4.492 RESP(Y)= 0.8214 HSQC= C9

H9B / 1 2.649820 1*1*1 STAT=Y PRED= 2.511 RANGE= 0.322 WIDTH(Y)= 4.286 RESP(Y)= 0.8442 HSQC= C9

H10 / 1 3.887114 1*1*1 STAT=Y PRED= 3.528 RANGE= 0.380 WIDTH(Y)= 4.485 RESP(Y)= 0.8369 HSQC= C10

H12A/ 1 1.850433 1*1*1 STAT=Y PRED= 1.519 RANGE= 0.310 WIDTH(Y)= 3.442 RESP(Y)= 0.9886 HSQC= C12

H12B/ 1 2.054114 1*1*1 STAT=Y PRED= 1.595 RANGE= 0.317 WIDTH(Y)= 4.589 RESP(Y)= 0.8266 HSQC= C12

H14 / 1 1.223384 1*1*3 STAT=Y PRED= 0.961 RANGE= 0.202 WIDTH(Y)= 3.758 RESP(Y)= 0.9006 HSQC= C14

H15 / 1 4.372085 1*1*1 STAT=Y PRED= 4.207 RANGE= 0.387 WIDTH(Y)= 4.216 RESP(Y)= 0.8368 HSQC= C15

H18 / 1 5.535928 1*1*1 STAT=Y PRED= 5.371 RANGE= 0.855 WIDTH(Y)= 5.341 RESP(Y)= 0.9323 HSQC= C18

H19 / 1 1.861775 1*1*3 STAT=Y PRED= 1.707 RANGE= 0.210 WIDTH(Y)= 4.172 RESP(Y)= 0.8677 HSQC= C19

H20 / 1 2.735587 1*1*1 STAT=Y PRED= 2.592 RANGE= 0.370 WIDTH(Y)= 4.843 RESP(Y)= 0.9141 HSQC= C20

H21 / 1 5.088181 1*1*1 STAT=Y PRED= 4.893 RANGE= 0.402 WIDTH(Y)= 4.366 RESP(Y)= 1.0000 HSQC= C21

H24A/ 1 2.849615 1*1*1 STAT=Y PRED= 2.619 RANGE= 0.280 WIDTH(Y)= 4.477 RESP(Y)= 0.8525 HSQC= C24

H24B/ 1 2.849615 1*1*1 STAT=Y PRED= 2.506 RANGE= 0.300 WIDTH(Y)= 4.418 RESP(Y)= 0.8663 HSQC= C24

H25A/ 1 2.907764 1*1*1 STAT=Y PRED= 2.551 RANGE= 0.307 WIDTH(Y)= 4.789 RESP(Y)= 0.8417 HSQC= C25

H25B/ 1 2.902236 1*1*1 STAT=Y PRED= 2.699 RANGE= 0.322 WIDTH(Y)= 4.820 RESP(Y)= 0.8263 HSQC= C25

H28 / 1 1.186158 1*1*3 STAT=Y PRED= 1.254 RANGE= 0.297 WIDTH(Y)= 4.035 RESP(Y)= 0.9230 HSQC= C28

H29 / 1 0.991630 1*1*3 STAT=Y PRED= 0.867 RANGE= 0.237 WIDTH(Y)= 3.986 RESP(Y)= 0.8538 HSQC= C29

COUPLING CONSTANTS(HZ):

J32_33 8.1188 J H1 H2A STAT=Y PRED= 5.581 RANGE= 3.690

J32_34 3.3621 J H1 H2B STAT=Y PRED= 5.581 RANGE= 3.700

J32_43 5.8350 J H1 H10 STAT=Y PRED= 3.352 RANGE= 3.700

J33_34 -14.1297 J H2A H2B STAT=Y PRED= -13.869 RANGE= 1.520

J33_35 7.4481 J H2A H3 STAT=Y PRED= 7.232 RANGE= 2.000

J34_35 7.2353 J H2B H3 STAT=Y PRED= 7.052 RANGE= 2.000

J35_36 15.1874 J H3 H4 STAT=Y PRED= 16.224 RANGE= 1.260

J36_37 10.5338 J H4 H5 STAT=Y PRED= 10.529 RANGE= 1.920

J37_38 14.7864 J H5 H6 STAT=Y PRED= 15.374 RANGE= 1.010

J38_39 10.7279 J H6 H7 STAT=Y PRED= 10.529 RANGE= 1.920

J39_40 14.8073 J H7 H8 STAT=Y PRED= 16.234 RANGE= 1.270

J40_41 7.1001 J H8 H9A STAT=Y PRED= 7.223 RANGE= 2.000

J40_42 7.4722 J H8 H9B STAT=Y PRED= 7.001 RANGE= 2.000

J41_42 -14.1071 J H9A H9B STAT=Y PRED= -13.895 RANGE= 1.520

J41_51 6.3390 J H9A H15 STAT=Y PRED= 7.150 RANGE= 2.000

J42_51 6.6941 J H9B H15 STAT=Y PRED= 5.536 RANGE= 2.000

J43_45 8.7157 J H10 H12A STAT=Y PRED= 5.792 RANGE= 2.000

J43_46 2.6947 J H10 H12B STAT=Y PRED= 7.024 RANGE= 2.000

J45_46 -13.6094 J H12A H12B STAT=Y PRED= -14.111 RANGE= 1.000

J45_48 7.4183 J H12A H14 STAT=Y PRED= 7.464 RANGE= 1.000

J46_48 7.3551 J H12B H14 STAT=Y PRED= 7.464 RANGE= 1.000

J53_57 9.6006 J H18 H20 STAT=Y PRED= 7.655 RANGE= 2.000

J57_58 5.7500 J H20 H21 STAT=Y PRED= 3.086 RANGE= 2.080

J57_67 6.7551 J H20 H29 STAT=Y PRED= 6.885 RANGE= 1.000

J58_64 6.3504 J H21 H28 STAT=Y PRED= 6.253 RANGE= 1.000

J59_60 -18.0972 J H24A H24B STAT=Y PRED= -16.354 RANGE= 1.260

J59_61 8.5647 J H24A H25A STAT=Y PRED= 6.489 RANGE= 2.000

J59_62 4.8343 J H24A H25B STAT=Y PRED= 7.527 RANGE= 2.000

J60_61 7.1287 J H24B H25A STAT=Y PRED= 6.259 RANGE= 2.000

J60_62 6.3302 J H24B H25B STAT=Y PRED= 6.461 RANGE= 2.000

J61_62 -15.3842 J H25A H25B STAT=Y PRED= -16.333 RANGE= 1.270

CONTROL PARAMETERS:

Solvent = none (def. 99% enriched)

1.000 = Concentration (vol%, def=1.0%)

0.00100000 = Minimum line-intensity

0.00100000 = Diagonalization criterium (not in use)

600.13000000 = FIELD(1H,MHz), used to transform shifts to ppms

15.53463340 = Left frequency (ppm)

-4.49276749 = Right frequency (ppm)

0.000 = Acquisition time (s, for QMTLS)

3.687 = Line-width (for modes D, P & T, 0=use defaults)

0.183398907 = Data-point resolution (Hz)

69.352 = GAUSSIAN (%, 0=use default from INF)

-5.051 = Dispersion contribution (%, 0=use default from INF)

0.00000000 = Decoupling frequency (for DORES)

END of FILE

**Figure S45.** The ^1^H NMR fingerprint of **2** (PERCH .pms file format).

ACTIVE SPECIES:1H

CHEMICAL SHIFTS(PPM):

PROTON 2*SPIN= 1 SPECIES=1H POPULATION(Y)= 0.01725

H1 / 1 4.492234 1*1*1 STAT=Y PRED= 4.308 RANGE= 0.447 WIDTH(Y)= 1.866 RESP(Y)= 1.0000 HSQC= C1

H2A / 1 2.659062 1*1*1 STAT=Y PRED= 2.577 RANGE= 0.397 WIDTH(Y)= 4.008 RESP(Y)= 1.0000 HSQC= C2

H2B / 1 2.780307 1*1*1 STAT=Y PRED= 2.542 RANGE= 0.405 WIDTH(Y)= 3.385 RESP(Y)= 1.0000 HSQC= C2

H3 / 1 5.977844 1*1*1 STAT=Y PRED= 6.016 RANGE= 0.795 WIDTH(Y)= 1.903 RESP(Y)= 1.0000 HSQC= C3

H4 / 1 6.325516 1*1*1 STAT=Y PRED= 6.096 RANGE= 0.815 WIDTH(Y)= 2.617 RESP(Y)= 1.0000 HSQC= C4

H5 / 1 6.241401 1*1*1 STAT=Y PRED= 6.111 RANGE= 0.785 WIDTH(Y)= 4.116 RESP(Y)= 1.0000 HSQC= C5

H6 / 1 6.290194 1*1*1 STAT=Y PRED= 6.169 RANGE= 0.805 WIDTH(Y)= 1.368 RESP(Y)= 1.0000 HSQC= C6

H7 / 1 6.270833 1*1*1 STAT=Y PRED= 5.853 RANGE= 0.515 WIDTH(Y)= 2.855 RESP(Y)= 1.0000 HSQC= C7

H8 / 1 5.965224 1*1*1 STAT=Y PRED= 5.469 RANGE= 0.875 WIDTH(Y)= 2.702 RESP(Y)= 1.0000 HSQC= C8

H9A / 1 2.560573 1*1*1 STAT=Y PRED= 2.368 RANGE= 0.387 WIDTH(Y)= 3.595 RESP(Y)= 1.0000 HSQC= C9

H9B / 1 2.644330 1*1*1 STAT=Y PRED= 2.408 RANGE= 0.370 WIDTH(Y)= 3.944 RESP(Y)= 1.0000 HSQC= C9

H12A / 1 2.720606 1*1*1 STAT=Y PRED= 2.070 RANGE= 0.362 WIDTH(Y)= 2.612 RESP(Y)= 1.0000 HSQC= C12

H12B / 1 2.754929 1*1*1 STAT=Y PRED= 2.599 RANGE= 0.302 WIDTH(Y)= 1.434 RESP(Y)= 1.0000 HSQC= C12

H13 / 1 1.089044 1*1*3 STAT=Y PRED= 0.945 RANGE= 0.272 WIDTH(Y)= 1.463 RESP(Y)= 1.0000 HSQC= C13

H14 / 1 4.376674 1*1*1 STAT=Y PRED= 4.164 RANGE= 0.397 WIDTH(Y)= 2.744 RESP(Y)= 1.0000 HSQC= C14

H17 / 1 5.558017 1*1*1 STAT=Y PRED= 6.398 RANGE= 1.020 WIDTH(Y)= 3.968 RESP(Y)= 1.0000 HSQC= C17

H18 / 1 1.861561 1*1*3 STAT=Y PRED= 1.570 RANGE= 0.285 WIDTH(Y)= 2.537 RESP(Y)= 1.0000 HSQC= C18

H19 / 1 2.740261 1*1*1 STAT=Y PRED= 3.562 RANGE= 0.460 WIDTH(Y)= 3.645 RESP(Y)= 1.0000 HSQC= C19

H20 / 1 5.104106 1*1*1 STAT=Y PRED= 4.159 RANGE= 0.437 WIDTH(Y)= 2.321 RESP(Y)= 1.0000 HSQC= C20

H23A / 1 2.852530 1*1*1 STAT=Y PRED= 2.894 RANGE= 0.410 WIDTH(Y)= 2.893 RESP(Y)= 1.0000 HSQC= C23

H23B / 1 2.852530 1*1*1 STAT=Y PRED= 2.450 RANGE= 0.320 WIDTH(Y)= 2.694 RESP(Y)= 1.0000 HSQC= C23

H24A / 1 2.906049 1*1*1 STAT=Y PRED= 2.571 RANGE= 0.282 WIDTH(Y)= 3.566 RESP(Y)= 1.0000 HSQC= C24

H24B / 1 2.917212 1*1*1 STAT=Y PRED= 2.506 RANGE= 0.377 WIDTH(Y)= 3.621 RESP(Y)= 1.0000 HSQC= C24

H27 / 1 1.194425 1*1*3 STAT=Y PRED= 1.202 RANGE= 0.302 WIDTH(Y)= 2.039 RESP(Y)= 1.0000 HSQC= C27

H28 / 1 1.006914 1*1*3 STAT=Y PRED= 1.117 RANGE= 0.242 WIDTH(Y)= 2.236 RESP(Y)= 1.0000 HSQC= C28

OH 2*SPIN= 1 SPECIES=1H POPULATION(N)= 0.98275

water/ 2 4.944046 1*1*1 STAT=Y PRED= 6.092 RANGE= 0.760 WIDTH(Y)= 80.000 RESP(Y)= 1.0000 HSQC= C1

COUPLING CONSTANTS(HZ):

J32_33 4.8774 J H1 H2A STAT=Y PRED= 5.130 RANGE= 2.000

J32_34 7.2651 J H1 H2B STAT=Y PRED= 5.802 RANGE= 2.630

J33_34 -13.4908 J H2A H2B STAT=Y PRED= -13.814 RANGE= 1.530

J33_35 7.4102 J H2A H3 STAT=Y PRED= 7.093 RANGE= 2.000

J34_35 7.0004 J H2B H3 STAT=Y PRED= 7.233 RANGE= 2.000

J35_36 15.1413 J H3 H4 STAT=Y PRED= 16.256 RANGE= 1.280

J36_37 10.7186 J H4 H5 STAT=Y PRED= 10.529 RANGE= 1.920

J37_38 15.0480 J H5 H6 STAT=Y PRED= 15.374 RANGE= 1.010

J38_39 10.6290 J H6 H7 STAT=Y PRED= 10.529 RANGE= 1.920

J39_40 14.6618 J H7 H8 STAT=Y PRED= 16.234 RANGE= 1.270

J40_41 7.1155 J H8 H9A STAT=Y PRED= 7.290 RANGE= 2.000

J40_42 6.9321 J H8 H9B STAT=Y PRED= 7.227 RANGE= 2.000

J41_42 -13.7859 J H9A H9B STAT=Y PRED= -13.900 RANGE= 1.530

J41_49 7.0982 J H9A H14 STAT=Y PRED= 7.149 RANGE= 2.000

J42_49 6.0646 J H9B H14 STAT=Y PRED= 5.540 RANGE= 2.000

J44_45 -14.8884 J H12A H12B STAT=Y PRED= -15.489 RANGE= 2.140

J44_46 7.4494 J H12A H13 STAT=Y PRED= 7.202 RANGE= 1.000

J45_46 7.0502 J H12B H13 STAT=Y PRED= 7.202 RANGE= 1.000

J51_55 9.9671 J H17 H19 STAT=Y PRED= 7.419 RANGE= 2.000

J55_56 5.2685 J H19 H20 STAT=Y PRED= 3.219 RANGE= 2.260

J55_65 6.8604 J H19 H28 STAT=Y PRED= 6.885 RANGE= 1.000

J56_62 6.3287 J H20 H27 STAT=Y PRED= 6.253 RANGE= 1.000

J57_58 -16.8793 J H23A H23B STAT=Y PRED= -16.380 RANGE= 1.260

J57_59 4.4709 J H23A H24A STAT=Y PRED= 6.348 RANGE= 2.000

J57_60 8.7875 J H23A H24B STAT=Y PRED= 7.248 RANGE= 2.000

J58_59 6.2584 J H23B H24A STAT=Y PRED= 7.256 RANGE= 2.000

J58_60 7.0131 J H23B H24B STAT=Y PRED= 6.348 RANGE= 2.000

J59_60 -16.8889 J H24A H24B STAT=Y PRED= -16.337 RANGE= 1.270

CONTROL PARAMETERS:

Solvent = none (def. 99% enriched)

1.000 = Concentration (vol%, def=1.0%)

0.00100000 = Minimum line-intensity

0.00100000 = Diagonalization criterium (not in use)

600.17220000 = FIELD(1H,MHz), used to transform shifts to ppms

15.09382229 = Left frequency (ppm)

-9.93733403 = Right frequency (ppm)

0.000 = Acquisition time (s, for QMTLS)

1.713 = Line-width (for modes D, P & T, 0=use defaults)

0.458479777 = Data-point resolution (Hz)

9.964 = GAUSSIAN (%, 0=use default from INF)

2.417 = Dispersion contribution (%, 0=use default from INF)

0.00000000 = Decoupling frequency (for DORES)

END of FILE

**Figure S46.** The ^1^H NMR fingerprint of **3** (PERCH .pms file format).

ACTIVE SPECIES:1H

CHEMICAL SHIFTS(PPM):

PROTON 2*SPIN= 1 SPECIES=1H POPULATION(Y)= 1.00000

H1 / 1 4.028200 1*1*1 STAT=Y PRED= 3.608 RANGE= 0.400 WIDTH(Y)= 2.876 RESP(Y)= 0.7271 HSQC= C1

H2A / 1 2.746044 1*1*1 STAT=Y PRED= 2.267 RANGE= 0.365 WIDTH(Y)= 3.015 RESP(Y)= 0.6485 HSQC= C2

H2B / 1 2.886990 1*1*1 STAT=Y PRED= 2.200 RANGE= 0.365 WIDTH(Y)= 3.174 RESP(Y)= 0.6927 HSQC= C2

H3 / 1 6.209067 1*1*1 STAT=Y PRED= 5.765 RANGE= 0.855 WIDTH(Y)= 2.735 RESP(Y)= 0.7638 HSQC= C3

H4 / 1 6.370580 1*1*1 STAT=Y PRED= 6.221 RANGE= 0.567 WIDTH(Y)= 3.727 RESP(Y)= 0.7826 HSQC= C4

H5 / 1 6.318625 1*1*1 STAT=Y PRED= 6.382 RANGE= 0.557 WIDTH(Y)= 3.008 RESP(Y)= 0.8249 HSQC= C5

H6 / 1 6.273819 1*1*1 STAT=Y PRED= 6.348 RANGE= 0.875 WIDTH(Y)= 2.618 RESP(Y)= 0.7940 HSQC= C6

H7 / 1 6.297358 1*1*1 STAT=Y PRED= 6.018 RANGE= 0.550 WIDTH(Y)= 2.908 RESP(Y)= 0.7089 HSQC= C7

H8 / 1 5.994802 1*1*1 STAT=Y PRED= 5.480 RANGE= 0.875 WIDTH(Y)= 2.588 RESP(Y)= 0.7289 HSQC= C8

H9A / 1 2.689003 1*1*1 STAT=Y PRED= 2.458 RANGE= 0.375 WIDTH(Y)= 3.701 RESP(Y)= 0.7289 HSQC= C9

H9B / 1 2.612961 1*1*1 STAT=Y PRED= 2.304 RANGE= 0.367 WIDTH(Y)= 3.045 RESP(Y)= 0.9553 HSQC= C9

H10 / 1 3.887246 1*1*1 STAT=Y PRED= 3.449 RANGE= 0.312 WIDTH(Y)= 2.957 RESP(Y)= 0.7894 HSQC= C10

H11 / 1 6.140779 1*1*1 STAT=Y PRED= 4.294 RANGE= 2.993 WIDTH(Y)= 2.237 RESP(Y)= 0.7782

H12A/ 1 1.853299 1*1*1 STAT=Y PRED= 1.309 RANGE= 0.307 WIDTH(Y)= 3.368 RESP(Y)= 1.0000 HSQC= C12

H12B/ 1 2.061987 1*1*1 STAT=Y PRED= 1.407 RANGE= 0.312 WIDTH(Y)= 2.574 RESP(Y)= 0.7022 HSQC= C12

H13 / 1 6.044781 1*1*1 STAT=Y PRED= 4.626 RANGE= 2.313 WIDTH(Y)= 2.054 RESP(Y)= 0.7214

H14 / 1 1.226552 1*1*3 STAT=Y PRED= 0.796 RANGE= 0.195 WIDTH(Y)= 3.015 RESP(Y)= 0.8277 HSQC= C14

H15 / 1 4.448205 1*1*1 STAT=Y PRED= 4.183 RANGE= 0.390 WIDTH(Y)= 3.209 RESP(Y)= 0.7476 HSQC= C15

H17 / 1 6.415858 1*1*1 STAT=Y PRED= 4.889 RANGE= 2.140 WIDTH(Y)= 1.901 RESP(Y)= 0.7157

H18 / 1 5.842163 1*1*1 STAT=Y PRED= 5.026 RANGE= 0.750 WIDTH(Y)= 4.289 RESP(Y)= 0.8245 HSQC= C18

H19 / 1 1.878159 1*1*3 STAT=Y PRED= 1.565 RANGE= 0.247 WIDTH(Y)= 3.377 RESP(Y)= 0.8211 HSQC= C19

H20 / 1 2.698752 1*1*1 STAT=Y PRED= 2.370 RANGE= 0.372 WIDTH(Y)= 3.466 RESP(Y)= 0.9539 HSQC= C20

H21 / 1 3.966550 1*1*1 STAT=Y PRED= 3.679 RANGE= 0.410 WIDTH(Y)= 3.708 RESP(Y)= 0.9902 HSQC= C21

H22 / 1 5.702118 1*1*1 STAT=Y PRED= 3.707 RANGE= 2.563 WIDTH(Y)= 2.056 RESP(Y)= 0.7346

H23 / 1 1.315823 1*1*3 STAT=Y PRED= 0.836 RANGE= 0.277 WIDTH(Y)= 3.289 RESP(Y)= 0.9221 HSQC= C23

H24 / 1 1.190459 1*1*3 STAT=Y PRED= 0.849 RANGE= 0.222 WIDTH(Y)= 3.231 RESP(Y)= 0.8820 HSQC= C24

COUPLING CONSTANTS(HZ):

J25_26 8.4639 J H1 H2A STAT=Y PRED= 5.062 RANGE= 2.000

J25_27 3.4450 J H1 H2B STAT=Y PRED= 6.316 RANGE= 3.310

J25_36 5.8469 J H1 H10 STAT=Y PRED= 5.442 RANGE= 2.230

J25_37 5.7429 J H1 H11 STAT=Y PRED= 4.323 RANGE= 2.000

J26_27 -14.3182 J H2A H2B STAT=Y PRED= -14.323 RANGE= 1.120

J26_28 7.1644 J H2A H3 STAT=Y PRED= 7.227 RANGE= 2.000

J27_28 7.3774 J H2B H3 STAT=Y PRED= 7.126 RANGE= 2.000

J28_29 15.2087 J H3 H4 STAT=Y PRED= 16.239 RANGE= 1.320

J29_30 10.5514 J H4 H5 STAT=Y PRED= 9.726 RANGE= 2.210

J30_31 14.8570 J H5 H6 STAT=Y PRED= 15.392 RANGE= 1.070

J31_32 10.6152 J H6 H7 STAT=Y PRED= 10.489 RANGE= 2.040

J32_33 14.8009 J H7 H8 STAT=Y PRED= 16.246 RANGE= 1.320

J33_34 7.0036 J H8 H9A STAT=Y PRED= 7.015 RANGE= 2.000

J33_35 7.9903 J H8 H9B STAT=Y PRED= 7.222 RANGE= 2.000

J34_35 -13.6751 J H9A H9B STAT=Y PRED= -14.889 RANGE= 1.790

J34_44 7.0684 J H9A H15 STAT=Y PRED= 7.370 RANGE= 2.000

J35_44 5.5078 J H9B H15 STAT=Y PRED= 5.338 RANGE= 3.940

J36_38 8.8702 J H10 H12A STAT=Y PRED= 6.985 RANGE= 2.000

J36_39 3.0989 J H10 H12B STAT=Y PRED= 5.662 RANGE= 2.000

J36_40 5.8951 J H10 H13 STAT=Y PRED= 3.854 RANGE= 2.000

J38_39 -13.5580 J H12A H12B STAT=Y PRED= -14.353 RANGE= 1.120

J38_41 7.3696 J H12A H14 STAT=Y PRED= 7.379 RANGE= 1.000

J39_41 7.3469 J H12B H14 STAT=Y PRED= 7.379 RANGE= 1.000

J44_45 3.8332 J H15 H17 STAT=Y PRED= 3.845 RANGE= 2.630

J46_50 9.6182 J H18 H20 STAT=Y PRED= 7.657 RANGE= 2.000

J50_51 5.0017 J H20 H21 STAT=Y PRED= 3.149 RANGE= 2.210

J50_56 6.8471 J H20 H24 STAT=Y PRED= 6.723 RANGE= 1.000

J51_52 4.6041 J H21 H22 STAT=Y PRED= 3.867 RANGE= 3.320

J51_53 6.2301 J H21 H23 STAT=Y PRED= 6.370 RANGE= 1.000

CONTROL PARAMETERS:

Solvent = none (def. 99% enriched)

1.000 = Concentration (vol%, def=1.0%)

0.00100000 = Minimum line-intensity

0.00100000 = Diagonalization criterium (not in use)

600.13000000 = FIELD(1H,MHz), used to transform shifts to ppms

15.04902013 = Left frequency (ppm)

-4.97838440 = Right frequency (ppm)

0.000 = Acquisition time (s, for QMTLS)

1.970 = Line-width (for modes D, P & T, 0=use defaults)

0.183398907 = Data-point resolution (Hz)

66.333 = GAUSSIAN (%, 0=use default from INF)

-8.840 = Dispersion contribution (%, 0=use default from INF)

0.00000000 = Decoupling frequency (for DORES)

END of FILE

**Figure S47.** The ^1^H NMR fingerprint of **4** (PERCH .pms file format).
